# Pathogen-specific immune responses might underlie divergent outcomes of coronavirus and influenza infection in the natural porcine host

**DOI:** 10.1038/s42003-026-10400-y

**Published:** 2026-05-30

**Authors:** Ehsan Sedaghat-Rostami, Liu Yang, Ashutosh Vats, Basudev Paudyal, Emily Briggs, Brigid Veronica Carr, Graham Freimanis, Ines Ruedas-Torres, Tim Downing, Christine Rollier, Katherine Marougka, Cornelis A. M. De Haan, Jai Mehat, Roberto La Ragione, Andrew Muir, Arianne C. Richard, Kristien Van Reeth, Francisco J. Salguero, Wilhelm Gerner, Elma Tchilian

**Affiliations:** 1 Host Response, The Pirbright Institute, Pirbright, UK; 2 School of Biosciences, Faculty of Health and Medical Sciences, University of Surrey, Guildford, UK; 3 United Kingdom Health Security Agency, UKHSA-Porton Down, Salisbury, UK; 4 Department of Biomolecular Health Sciences, Faculty of Veterinary Medicine, Utrecht University, Utrecht, The Netherlands; 5 School of Veterinary Medicine, Faculty of Health and Medical Sciences, University of Surrey, Guildford, UK; 6 Immunology Programme, Babraham Institute, Cambridge, UK; 7 Department of Translational Physiology, Infectiology and Public Health, Faculty of Veterinary Medicine, Ghent University, Ghent, Belgium

**Keywords:** Viral infection, Mucosal immunology

## Abstract

Coronaviruses and influenza A viruses are major respiratory pathogens with pandemic potential. Using pigs as a translational large-animal model, we compare the virulence, pathogenesis, and immune responses to porcine respiratory coronavirus (PRCV) and pandemic H1N1 2009 influenza virus (pH1N1). Here we show that PRCV induces higher viral load and prolonged viral shedding, stronger systemic and mucosal T cell activation, expansion of memory B cells, and distinct nasal microbiome changes. In contrast, pH1N1 results in rapid neutralising antibody production, robust Tfh and germinal centre B cell responses, and broader early nasal microbial diversity. Transcriptional responses to PRCV and pH1N1 infection start with the activation of shared interferon-stimulated genes but later diverge as pathways involving stromal-immune interactions and vascular integrity shapes lung pathology and subsequent immune responses. These findings demonstrate fundamental differences in coronavirus and influenza virus-host interactions and establish the pig as a powerful comparative model for studying respiratory virus pathogenesis and immunity.

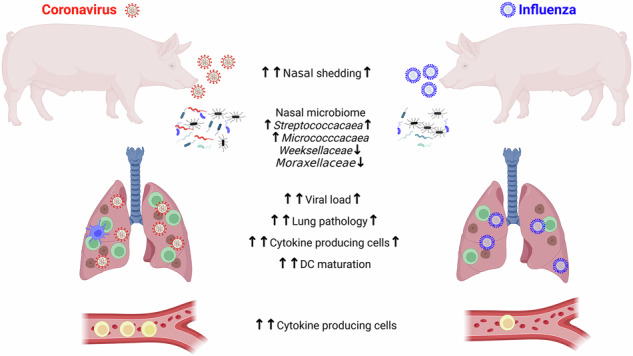

## Abstract (web summary)

Pig natural host model comparing coronavirus and influenza infection across lung pathology, viral load, T and B cell responses, nasal microbiome shifts, and single cell RNA defined lung pathways.

## Introduction

Influenza viruses and coronaviruses (CoVs) are major global health threats for both animals and humans, responsible for seasonal epidemics and occasional pandemics^1,2^. These pathogens produce a broad spectrum of disease in humans, ranging from asymptomatic or mild infections to severe acute respiratory distress syndrome and death, particularly among immunocompromised individuals, the elderly, and those with underlying health conditions^3,4^. Viruses from both families that infect humans originate in animal reservoirs and have repeatedly crossed species barriers, as illustrated by avian and swine influenza viruses, Middle Eastern respiratory syndrome coronavirus (MERS-CoV), severe acute respiratory syndrome coronavirus (SARS-CoV), and the recent SARS-CoV-2 pandemic^5–8^.

The extent and severity of virus-induced lung injury are determined by complex interactions between viral and host factors. While the host immune response is essential for viral clearance, excessive or dysregulated inflammation can exacerbate tissue injury, leading to acute respiratory distress and chronic lung damage. Understanding the mechanisms that balance protective immunity, immune-mediated pathology, and tissue repair is critical for the development of targeted therapeutics and effective immunisation strategies. A range of animal models, mice, ferrets, hamsters, and non-human primates, have been developed to study the mechanisms of viral pathogenesis of influenza and respiratory CoV infections^9–11^. Each animal model offers specific advantages, but also limitations, such as differences in receptor distribution, immune responses, and pulmonary physiology. Furthermore, the fact that most laboratory animals are not natural hosts for these viruses means that findings from these models may not always directly translate to human infection.

The pig represents a highly relevant large-animal model for studying respiratory virus infections in humans. The porcine respiratory tract closely resembles that of humans in anatomy, airway architecture, and lung surface area^12,13^. Recent advances in xenotransplantation suggest that pigs might be a possible future source of lungs for humans^14^. Both species have similar distributions of sialic acid receptors, and pigs are naturally susceptible to influenza viruses that are antigenically and genetically related to human strains resulting in clinical signs, lung pathology and immune responses closely mirroring those in humans^13,15–17^. Although pigs are not susceptible to SARS-CoV-2 infection, they are natural hosts for porcine respiratory coronaviruses (PRCV) inducing lung pathology comparable to that caused by SARS-CoV and SARS-CoV-2 infections^18,19^. These parallels make pigs an excellent translational model for investigating host-pathogen interactions and evaluating immune responses to infection or the efficacy of vaccines, and therapeutics. Recent studies employing advanced immunological and transcriptomic approaches, have further demonstrated the power of the porcine model to dissect antiviral immunity^20–23^. Such analyses showed immune cell networks and gene expression signatures associated with severe lung pathology, providing mechanistic insights relevant for animal and human health.

Respiratory viral infections can also reshape the microbial communities of the upper and lower airways, and these changes may, in turn, influence disease severity, transmission, and the risk of secondary bacterial infections. Studies in humans have shown that influenza and SARS-CoV-2 infections are associated with characteristic alterations in nasal and airway microbiota composition, including expansion of opportunistic pathogens and loss of commensal taxa that may contribute to mucosal homeostasis^24,25^. A porcine model provides an opportunity to interrogate how distinct respiratory RNA viruses modulate the nasal microbial ecosystem, and to relate these microbial shifts to viral load, lung pathology, and host immune responses.

As both influenza viruses and CoV are respiratory pathogens, with overlapping clinical and pathological manifestations, a detailed comparative study may reveal differences that shed light on the mechanisms of protective immunity, inflammation and tissue repair. Comparative studies in humans are confounded by heterogeneity in genetic background, lifestyle, and prior exposure history. Here, we used the robust porcine natural host model to compare infection kinetics, lung pathology, nasal microbiome and immune responses following influenza and PRCV infection, uncovering shared and divergent mechanisms of respiratory virus pathogenesis and immune control. Our findings further support the use of the pig as an appropriate large natural host model for studying immunity to influenza and coronavirus infections.

## Results

### Viral load, respiratory pathology and immune cell infiltration following PRCV and pH1N1 infection

**Fig. 1:**
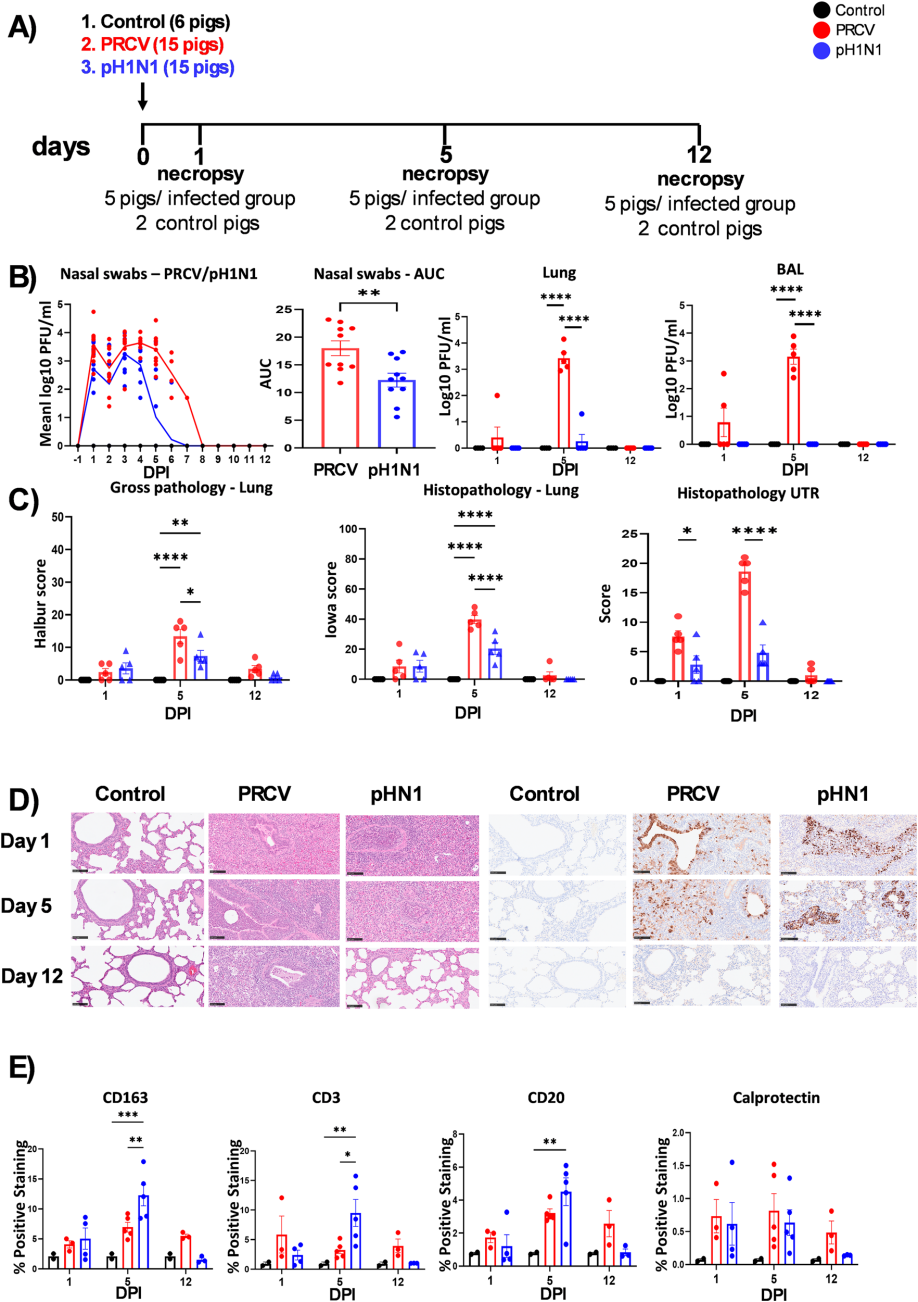
Experimental design, viral load, lung pathology, and immune cell infiltration. **A** 36 pigs were divided into 3 groups. The first group (*n* = 15) was infected intranasally with 8 × 10⁶ PFU per animal PRCV (red), the second group (*n* = 15) with 8 × 10⁶ PFU pH1N1 (blue), and the third group (*n* = 6) received PBS as a control (Black). At 1-, 5-, and 12-days post-infection (DPI), 5 pigs from each infected group and 2 from the control group were euthanized. **B** Viral load was quantified using plaque assays from daily nasal swabs and nasal shedding (area under the curve, AUC), lung tissue, and bronchoalveolar lavage (BAL) fluid titers are shown. **C** Gross pathology and immunohistochemistry were used to assess lung pathology, including detection of PRCV nucleocapsid (N) and pH1N1 nucleoprotein (NP) in the lungs, as well as histopathology of the upper respiratory tract. **D** Representative histopathology (H&E) and viral antigen detection (IHC) from 1-, 5-, and 12-DPI. PRCV nucleocapsid (N) and pH1N1 nucleoprotein (NP) antigens were stained in lung tissue. H&E and IHC were performed in serial sections. Bar = 100 µm. **E** Immunohistochemistry was performed on lung lesion sites to quantify CD163⁺ (macrophages), CD3⁺ (T cells), CD20⁺ (B cells), and Calprotectin⁺ (neutrophils) within the lesions. Each point represents an individual animal; bars indicate the mean ± SEM. Statistical analysis was performed using two-way ANOVA with a Bonferroni multiple comparison correction. Asterisks indicate significance (*p < 0.05, **p < 0.01, ***p < 0.001, ****p < 0.0001).

Fifteen pigs were infected intranasally either with PRCV or pH1N1 at an identical dose of 8×10^6^ PFU, while six received PBS as controls. At 1-, 5-, and 12- days post infection (DPI), 5 animals per infected group and 2 controls were culled to assess early (1 DPI) and late events (12 DPI) following infection (Fig. 1A). The five day time point was chosen as there is still significant viral load in the lungs and BAL and lung pathology is well developed^18,23,26^. Viral shedding was quantified by plaque assay of nasal swabs. Both viruses showed a transient drop in titres at 2 DPI, a pattern previously observed and possibly linked to early innate responses^27^. pH1N1 titres persisted until day 4 and cleared by day 7 (Fig. 1B and Supplementary Fig. 1A). PRCV was shed for 6 days and cleared by day 8. Plaque assays of lung tissue and BAL fluid confirmed higher PRCV viral load than pH1N1 at 5 DPI. No virus was detected in lung tissue or BAL fluid in either group at 12 DPI (Fig. 1B).

Lung pathology was assessed by gross and histological scoring as previously described^28^, including immunohistochemistry (IHC) for detection of nucleocapsid (N) for PRCV and nucleoprotein (NP) for pH1N1. PRCV induced more severe lung pathology at 5 DPI, with higher histopathology scores and broader involvement of the upper respiratory tract compared to pH1N1 (Fig. 1C, D and Supplementary Fig. 1B, C). In pH1N1-infected lungs, NP was mainly detected within the inflammatory infiltrates in airways and parenchyma showing thickened alveolar walls, while in PRCV-infected lungs, N was mainly detected in bronchial/bronchiolar epithelium, but also in inflammatory cells within the areas of broncho-interstitial pneumonia, especially at 5 DPI. No influenza NP or PRCV N was detected by IHC at 12 DPI. In both PRCV and pH1N1 infected lungs, necrosis and attenuation of the airway epithelia were evident at 1 and 5 DPI with a reduction in pan-cytokeratin expression by IHC. However, the number of pan-cytokeratin-stained cells was similar to control lungs at 12 DPC, indicating recovery of the epithelial lining of the lower respiratory tract (Supplementary Fig. 2A). The inflammatory cell infiltration in the lungs of both PRCV and pH1N1 showed predominantly mononuclear cells, mainly macrophages, but also lymphoplasmacytic infiltration at 1 and 5 DPI (Fig. 1E and Supplementary Fig. 2B).

In control lungs, the main immune cells were macrophages (CD163+), followed by B (CD20+) and T (CD3+) cells, with fewer neutrophils (MAC387+) (Fig. 1E and Supplementary Fig. 2B.) In infected animals, immune cell numbers increased at all time points (1-, 5-, 12- DPI), especially within the areas of broncho-interstitial pneumonia (Fig. 1E). Comparison of areas with lesions showed increased CD163⁺ cells, CD3⁺ cell infiltration in pH1N1-infected lungs compared to PRCV and more CD20^+^ cells compared to controls (Fig. 1E).

In summary, PRCV infection resulted in higher and more prolonged viral shedding and greater viral load in the lungs and BAL. PRCV infected animals also exhibited increased severe respiratory pathology, with reduced mononuclear, T and B cell infiltration within the areas with lesions at the early stages of the disease. In contrast the lesioned areas of pH1N1 infected animals exhibited higher numbers of immune cells, which may be associated with reduced tissue damage.

### Antibody, B cell and Tfh responses following PRCV or pH1N1 infection

**Fig. 2:**
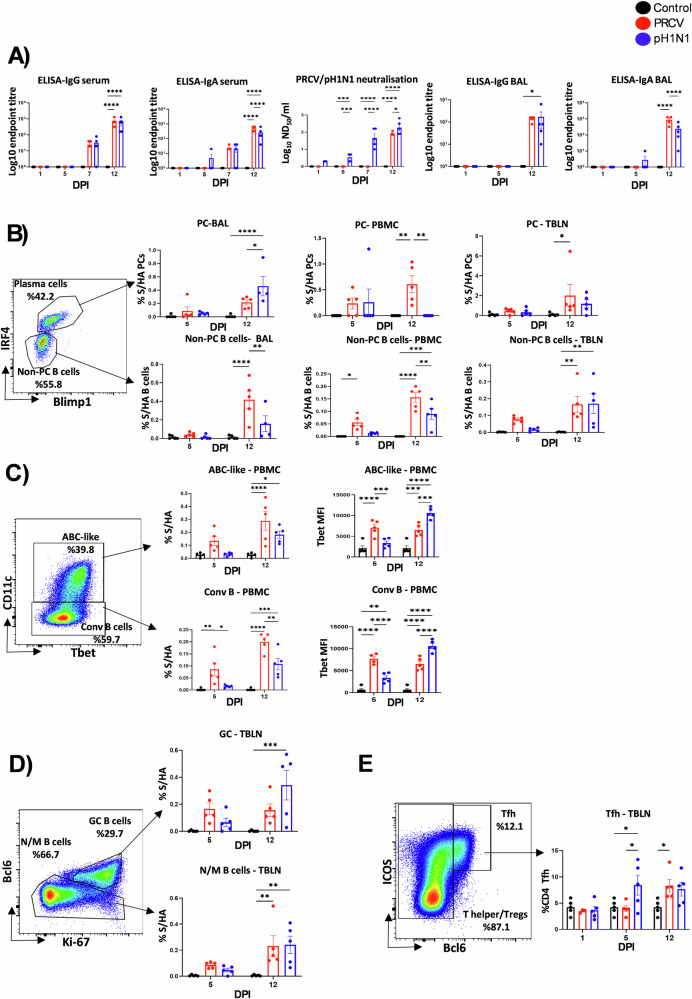
Antibody, B cell, and Tfh responses following PRCV or pH1N1 infection. **A** Spike (for PRCV) or hemagglutinin (HA for pH1N1)-specific IgG and IgA end-point titres were measured in serum and BAL fluid from control (black), PRCV (red), and pH1N1 (blue) infected pigs. 50% virus neutralization titres (ND50) were also determined in serum samples. Spike/HA-specific B cell responses were assessed in BAL, TBLN and PBMC using flow cytometry, including: **B** the proportion of spike/HA-specific plasma cells and B cells in BAL, PBMC, and TBLN; **C** the frequency of spike/HA-specific atypical-like and conventional B cells and the mean fluorescence intensity (MFI) of T-bet expression in PBMC and **D** the frequency of spike/HA-specific GC B cells and naïve/proliferating B cells in TBLN, following control (black), PRCV (red) or pH1N1 (blue) infections. **E** Frequency of T follicular helper (Tfh) cells in the TBLN of control (black), PRCV (red), and pH1N1 (blue) infected pigs. Each point represents an individual animal; bars indicate the mean ± SEM. Statistical analysis was performed using two-way ANOVA with a Bonferroni multiple comparison correction. Asterisks indicate significance (*p < 0.05, **p < 0.01, ***p < 0.001, ****p < 0.0001).

Antibody responses were assessed in serum and BAL after PRCV or pH1N1 infections by endpoint ELISA, measuring S or HA-specific IgG and IgA titers for PRCV and pH1N1, respectively. Both infections induced detectable IgG and IgA by 7 DPI in serum and by 12 DPI in BAL (Fig. 2A). One pH1N1-infected animal showed HA-specific IgA in serum at 5 DPI. In BAL, spike/HA-specific antibodies were detected at 12 DPI, except for one pH1N1-infected pig with HA-specific IgA at 5 DPI. Overall, spike-specific IgA titres were higher than HA-specific IgA in both serum and BAL (Fig. 2A). Virus neutralisation assays showed PRCV-neutralising antibodies in serum from 12 DPI, while pH1N1-neutralising antibodies were detectable at 5 DPI, with significantly higher titres than PRCV at 5, 7, and 12 DPI (Fig. 2A).

Flow cytometry was used to compare antigen-specific responses in different B cell subsets using methods established previously^29^. Total B cells (CD79α⁺PAX5⁺) were separated into PAX5⁺IRF4⁺ plasma cells and PAX5⁻IRF4⁻ non-plasma B cells (Supplementary Fig. 3A). In blood, within the IRF4⁻Blimp1⁻ population, both atypical B cell-like (ABC-like; CD11c⁺T-bet⁺/⁻) and conventional B cells (CD11c⁻T-bet⁺/^−^) were also assessed (Supplementary Fig. 3B). In TBLN, germinal centre (GC) B cells (Bcl-6⁺Ki-67⁺), and naïve/memory B cells (Bcl-6⁻Ki-67^−^^/+^) were identified (Supplementary Fig. 3C). Biotinylated spike and HA were used to detect antigen-specific B cells in these subsets.

At 12 DPI, pH1N1 infection induced a higher frequency of HA-specific plasma cells in BAL compared to PRCV and controls, whereas PRCV-infected animals had more spike-specific plasma cells in PBMC. PRCV also led to increased spike-specific non-plasma cells (PC) B cells in BAL and PBMC compared to pH1N1 and controls (Fig. 2B).

In PBMC, both PRCV and pH1N1 infection increased frequencies of spike/HA-specific ABC-like B cells, with T-bet MFI rising post-infection. T-bet expression was high in PRCV-induced ABC-like cells at 5 DPI and in pH1N1-infected cells at 12 DPI. Spike-specific conventional B cells also increased in PRCV at both 5 and 12 DPI, aligning with elevated T-bet MFI, while pH1N1 induced higher T-bet expression in conventional B cells at 12 DPI (Fig. 2C).

Analysis of GC and naïve/memory (N/M) B cells showed higher HA-specific GC B cells and increased spike/HA-specific N/M B cells in both infected groups at 12 DPI. Additionally, pH1N1 infection elicited more T follicular helper cells (CD4⁺ICOS⁺Bcl-6⁺) in TBLN at 5 DPI compared to PRCV and controls (Fig. 2D, E).

In summary, both PRCV and pH1N1 infections induced antibody, plasma cell, and B cell responses. However, pH1N1 triggered earlier and stronger neutralising antibody responses, associated with increased HA-specific plasma cells and Tfh cells. In contrast, PRCV infection, induced higher frequencies of spike-specific B cells in BAL and PBMC, suggesting a more robust local and systemic memory responses.

### T-cell cytokine responses in BAL and PBMC following PRCV and pH1N1 infection

**Fig. 3:**
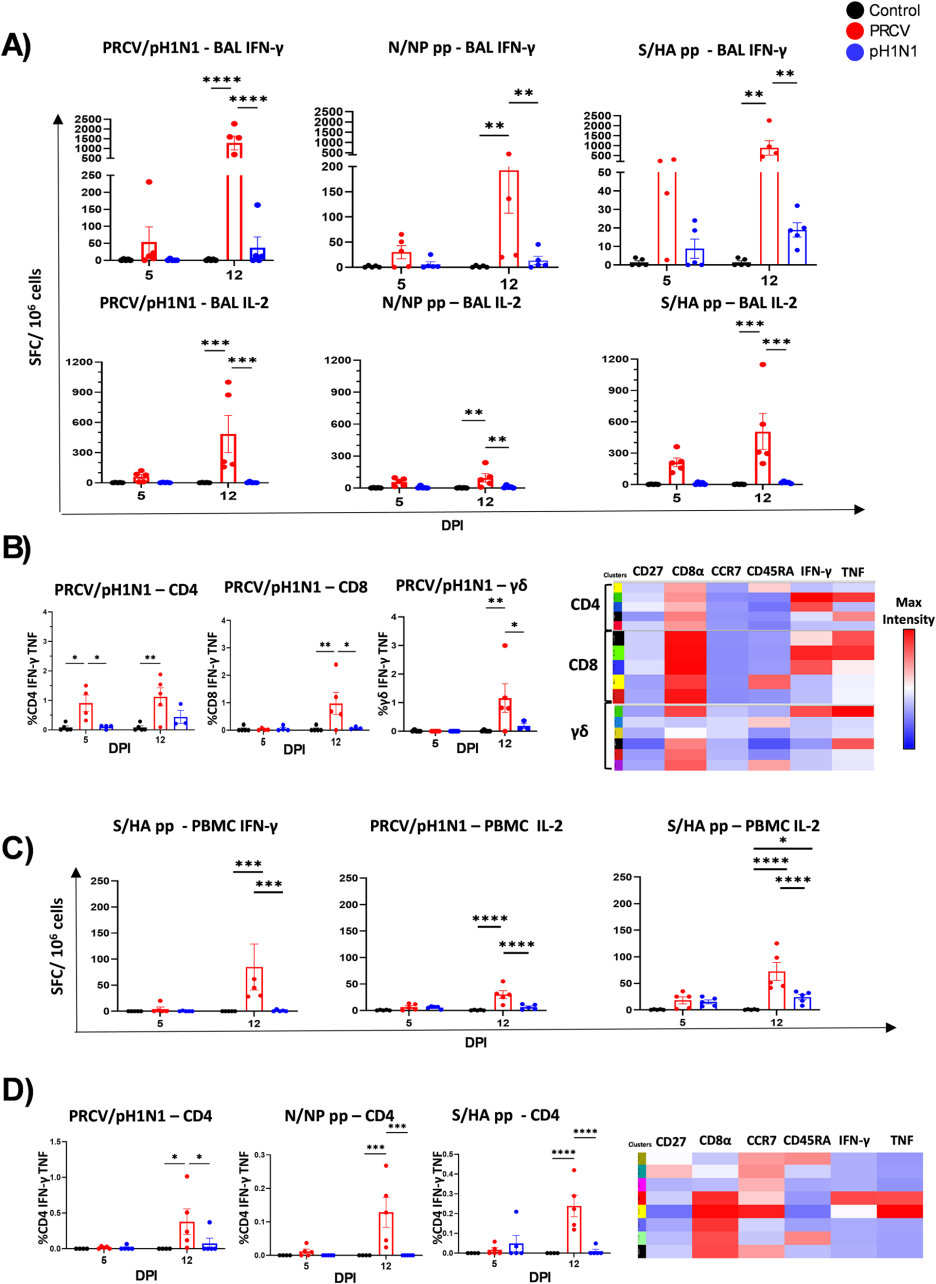
T cell cytokine responses in BAL and PBMC following PRCV and pH1N1 infections. **A**, **C** IFN-γ and IL-2 secreting spot-forming cells (SFC) were quantified by ELISpot following ex vivo stimulation with live PRCV/pH1N1 virus, PRCV nucleocapsid (N)/pH1N1 nucleoprotein (NP), or PRCV spike (S)/pH1N1 hemagglutinin (HA) peptide pools in BAL and PBMC from control (black), PRCV (red), pH1N1 (blue) infected animals**. B**, **D** FLOWSOM analysis heatmaps showing the phenotype and comparative proportions of IFN-γ- and TNF-secreting CD4⁺, CD8⁺, and γδ T cells in BAL and PBMC, as measured by flow cytometry in control (black), PRCV (red), pH1N1 (blue) pigs. Each point represents an individual animal; bars indicate the mean ± SEM. Statistical analysis was performed using two-way ANOVA with a Bonferroni multiple comparison correction. Asterisks indicate significance: (*p < 0.05, **p < 0.01, ***p < 0.001, ****p < 0.0001).

Cytokine-secreting cells in BAL and PBMC were analysed following ex vivo stimulation with either live PRCV and pH1N1 viruses or overlapping peptide pools covering the internal PRCV nucleocapsid (N pp) and pH1N1 nucleoprotein (NP pp), and the surface spike (S pp) and hemagglutinin (HA pp) proteins using ELISpot and intracellular cytokine staining (ICS) assays. At 12 DPI, the number of IFNγ and IL-2 producing cells responding to PRCV, N and S in BAL was significantly higher than in the pH1N1 and control groups, as measured by ELISpot assay (Fig. 3A).

ICS was used to identify the phenotype of cytokine-producing cells. We first looked at IFNγ and TNF co-production in the main T cell subsets, CD4+, CD8+, and γδ+, because this combination of cytokines is linked to potent antiviral T cell responses. The gating strategy and representative flow cytometry raw data for this are shown in Supplementary Fig. 4. Following stimulation with the respective live virus, there was an increased proportion of IFNγ⁺TNF⁺ CD4⁺ T cells at 5 and 12 DPI in PRCV-infected animals compared to pH1N1 and control groups (Fig. 3B). Similarly, the frequency of IFNγ⁺TNF⁺ CD8⁺ and γδ T cells was higher in the BAL of PRCV-infected animals at 12 DPI (Fig. 3B). For single-cytokine producing cells, there was a greater proportion of TNF-secreting CD4⁺ and γδ T cells following PRCV N stimulation at 5 and 12 DPI, respectively, and an increase in TNF⁺ CD8⁺ and γδ + T cells at 12 DPI following live PRCV stimulation, compared to pH1N1 and control groups (Supplementary Fig. 5A).

We also aimed to investigate the phenotype of cytokine-producing T cells in more detail. In pigs, antigen-experienced CD4⁺ T cells upregulate CD8α homodimers. Thus, the expression of CD8α was analysed with the differentiation-associated markers CCR7, CD45RA, and CD27; to define the phenotype of IFNγ⁺, TNF⁺, and IFNγ⁺TNF⁺ double-producing CD4⁺, CD8⁺, and γδ T cells (heatmap in Fig. 3B and Supplementary Fig. 5A). FlowSOM analysis showed that cytokine secreting CD4⁺ and γδ T cells displayed an effector memory phenotype (CD8α⁺CCR7⁻CD27⁻CD45RA⁻), which was also found in CD8⁺ T cells (CD45RA⁻CCR7⁻CD27⁻). We also performed ICS following PMA/ionomycin stimulation alongside peptide pool stimulation. No significant differences were observed in cytokine-secreting CD4⁺ and CD8⁺ T cells between control, PRCV-infected, and pH1N1-infected groups following PMA/ionomycin stimulation, indicating that the overall functional activity of T cells was not altered by PRCV or pH1N1 infection (Supplementary Fig. 6B).

In PBMCs, ELISpot assays showed increased IFNγ and IL-2 producing cells following stimulation with PRCV Spp at 12 DPI, compared to pH1N1 and control. There was also a higher frequency of IL-2 producing cells in response to live PRCV stimulation (Fig. 3C). ICS in blood revealed that at 12 DPI, the frequency of IFNγ⁺TNF⁺ double-producing CD4⁺ T cells was higher following stimulation with live PRCV, as well as PRCV N pp and S pp, compared to the same conditions in pH1N1 and control groups (Fig. 3D). However, at 5 DPI, TNF-secreting CD4⁺ T cells were more frequent following pH1N1 stimulation compared to PRCV and control (groups (Supplementary Fig. 7A). FlowSOM analysis of CD4 T cells in blood showed that IFNγ⁺TNF⁺ and TNF⁺ single producers both displayed a central memory phenotype (CD8α⁺ CCR7⁺CD27^-^CD45RA^-^) (heatmap, Fig. 3D and Supplementary Fig. 7B). ICS assays did not show cytokine-secreting for γδ and CD8 cells in PRCV and influenza infected pigs in blood (Supplementary Fig. 7C).

In summary, PRCV infection induced stronger cytokine responses in both BAL and PBMCs. A greater proportion of double IFNγ and TNF producing CD4⁺, CD8⁺, and γδ T cells were detected in the BAL, whereas in the blood, double-cytokine-producing cells were predominantly CD4⁺. In contrast, T cell responses following pH1N1 infection were largely confined to the respiratory tract, with fewer double-cytokine-producing cells detected.

### Dynamics of CD4⁺ T helper and regulatory T cells following PRCV and pH1N1 infections

**Fig. 4:**
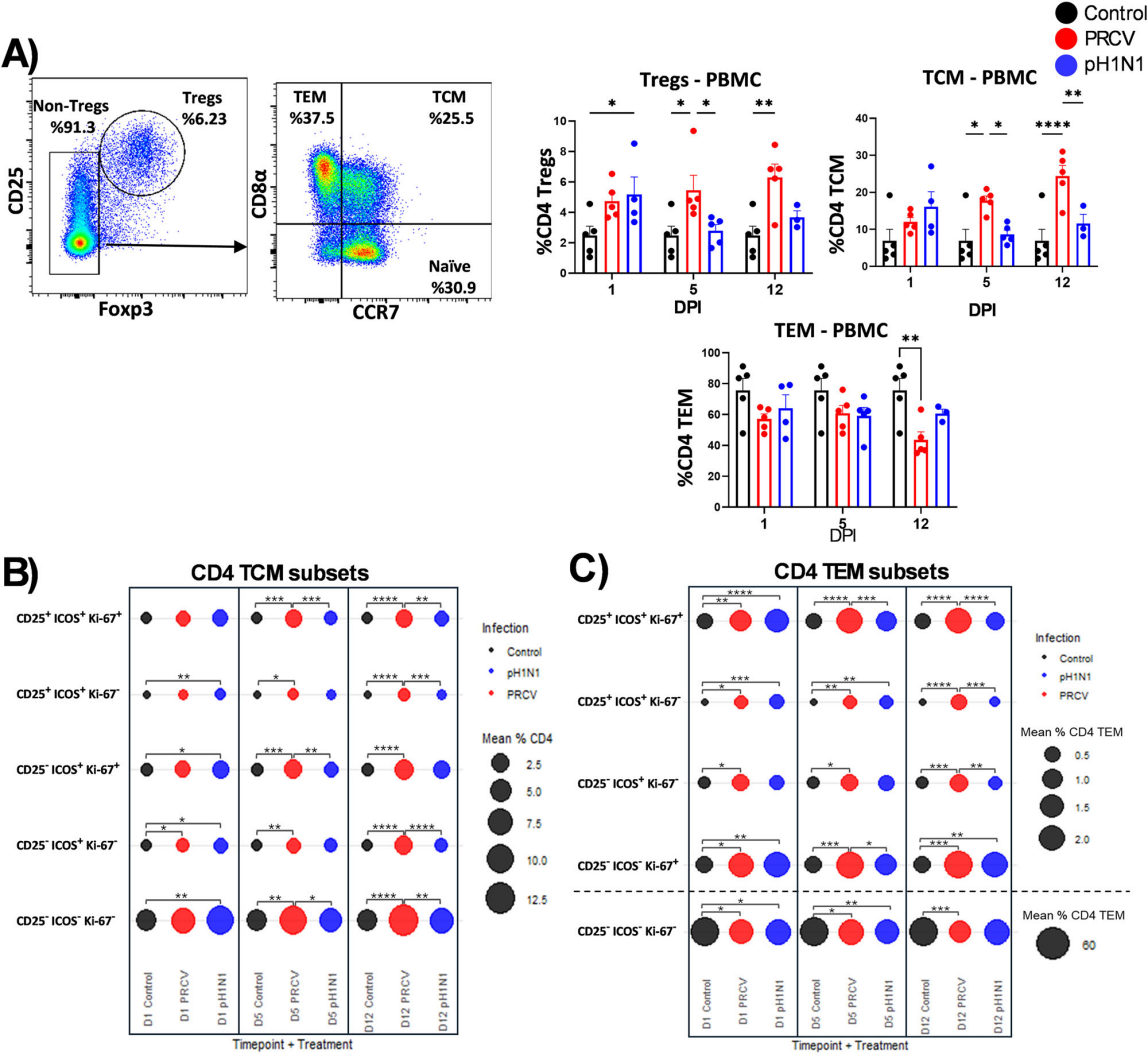
Comparative T cell phenotyping in PBMC following PRCV and pH1N1 infection. Flow cytometry was used to assess CD4⁺ T cell subset frequencies in peripheral blood mononuclear cells (PBMC) from control (black), PRCV (red), and pH1N1 (blue) pigs**. A** The frequencies of regulatory T cells (Tregs; FoxP3⁺CD25⁺), conventional CD4⁺ T helper cells (FoxP3⁻, Bcl6⁻), central memory T cells (CD4⁺CD8α⁺CCR7⁺), and effector memory T cells (CD4⁺CD8α⁺CCR7⁻) were quantified. **B**, **C** Bubble plots show the mean percentages of CD4⁺ central memory (TCM) and effector memory (TEM) T cells expressing activation or proliferation markers CD25, ICOS, or Ki-67. For **C**, two bubble size legends are shown because CD25^−^ICOS^−^Ki-67^−^ cells were substantially more abundant than the other four TEM subsets defined by CD25, ICOS, and Ki-67. Statistical analysis was performed using two-way ANOVA with a Bonferroni multiple comparison correction. Each point represents an individual animal; bars indicate the mean ± SEM. Asterisks indicate significance: (*p < 0.05, **p < 0.01, ***p < 0.001, ****p < 0.0001).

CD4⁺ T cells encompass several functional subsets, including regulatory T cells (Tregs) and follicular helper T cells (Tfh). In pigs, the co-expression of CCR7 and CD8α allows the classification of CD4⁺ T cells into naïve, central memory (TCM), and effector memory (TEM) subsets. Tregs can be identified by FOXP3 and CD25 co-expression. PRCV-infected animals exhibited a significant increase in Treg frequencies among PBMC at 5DPI compared to both pH1N1-infected and control animals (Fig. 4A). The frequency of CD8α^+^CCR7^+^ TCM significantly increased at 5 and 12 DPI compared to pH1N1 and control groups (Fig. 4A). Conversely, the frequency of CD8α^+^CCR7^-^TEM in PBMCs declined by 12 DPI.

To further characterise TCM and TEM CD4⁺ T cell subsets, we assessed the activation and proliferation markers CD25, ICOS, and Ki-67. t-SNE visualisation and by combinatorial gating on FOXP3⁻ BCL6⁻ CD4⁺ T cells were applied to BAL, TBLN, and PBMC (Supplementary Fig. 8A, B). Four major populations were identified: naïve (CCR7⁺CD8α⁻), TCM (CCR7⁺CD8α⁺), TEM (CCR7⁻CD8α⁺), and putative TEMRA (CCR7⁻CD8α⁻). All subsets were present in PBMCs and TBLNs, while BAL predominantly contained TEM and TEMRA cells, with minimal TCM and no detectable naïve cells (Supplementary Fig. 8B). For major CD25/ICOS/Ki-67 defined TCM and TEM subsets mean frequencies for each treatment group and time point were calculated and summarised in bubble plots (Supplementary Fig. 4B, C). Corresponding bar charts are displayed in Supplementary Fig. 8C. The analysis revealed an increased frequency of several activated TCM and TEM subsets in PRCV-infected animals, including CD25⁺ICOS⁺Ki-67⁺ triple-positive, CD25⁺ICOS⁺ double-positive, and Ki-67⁺ single-positive subsets. At the same time, non-activated (triple negative) TEM frequencies declined.

In summary, phenotypic analysis of CD4⁺ T cells in PBMCs revealed that PRCV infection induced a more prominent expansion of TCM and activated TEM T cells compared to pH1N1 and control groups. Additionally, the elevated frequency of Tregs suggested an increased presence of pro-resolving CD4⁺ subsets in response to PRCV-induced systemic inflammatory response.

### Single-cell RNA-seq identified multiple immune and non-immune cell subsets

We generated single-cell suspensions from lung tissue of control (*n* = 4), PRCV-infected (*n* = 4 at 1 DPI and *n* = 4 at 12 DPI), and pH1N1-inoculated (*n* = 3 at 1 DPI and *n* = 5 at 12 DPI) pigs, a total of 20 samples. Unsupervised clustering identified three broad cellular compartments: myeloid cells, lymphoid cells (T, B, and NKT-like cells), and non-immune cells (Supplementary Fig. 9A). Differences in the cellular compartment composition between the control, H1N1- and PRCV-infected samples (PERMANOVA *r*^2^ = 0.45 *p* = 0.048) were driven by a higher rate of myeloid cells in the infected samples at 1 DPI, which reverted to the baseline by 12 DPI (Supplementary Fig. 9B). Each of these three cellular compartments was further resolved into distinct cell clusters. For cluster annotation, we applied a curated panel of lineage-defining genes and assigned conventional cell-type identities across the 1 and 12 DPI datasets. 20 clusters were identified within myeloid cells, comprising monocytes and macrophages in different stages of differentiation, as well as dendritic cells (Supplementary Fig. 10). Sub-clustering of lymphoid cells resulted in nine clusters, including different T cell subsets, B cells and plasma cells (Supplementary Fig. 11). For non-immune cells, we identified 21 clusters, comprising a wide range of different cell types such as fibroblasts, endothelial cells, type 1 and 2 pneumocytes and smooth muscle cells (Supplementary Fig. 12).

### Early convergent transcriptional responses at 1 DPI

**Fig. 5:**
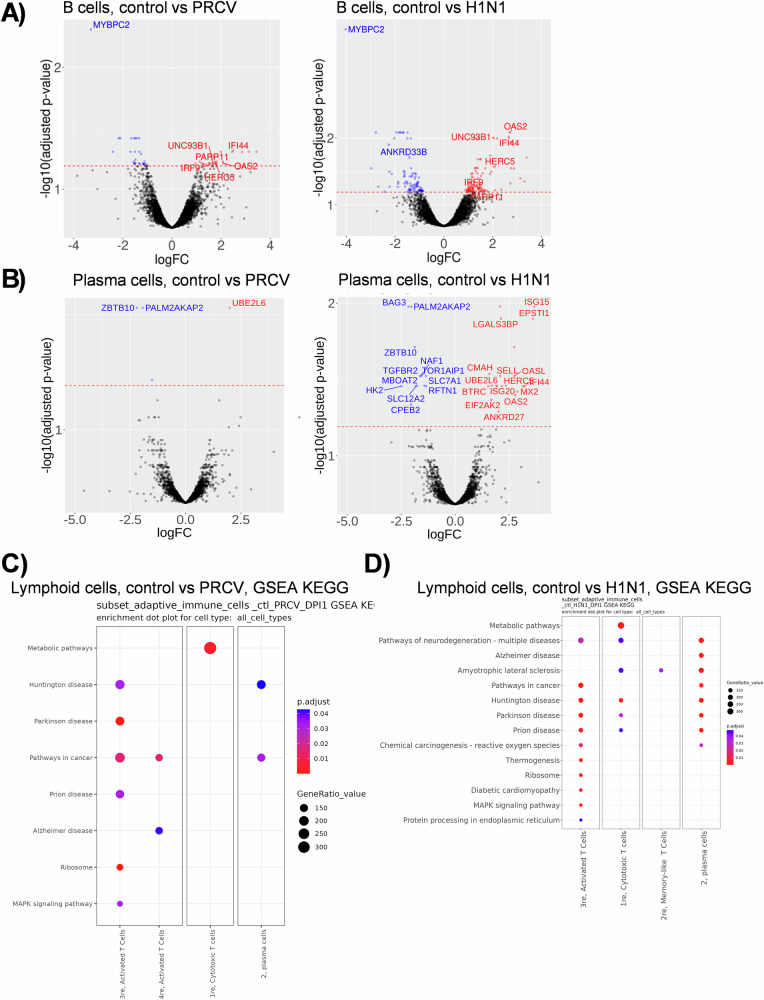
Adaptive immune cells respond at 1DPI to both infections. **A** Volcano plots of DE analysis for B cell cluster 10 showing PRCV vs control condition (left) and pH1N1 vs control (right). Red means the gene up regulated in H1N1/ PRCV when control is baseline. Blue means down-regulated. **B** As in (A) but for plasma cells (cluster 2) with PRCV vs control (left) and pH1N1 vs control (right). Bubble plots presenting enriched KEGG pathways across lymphoid cell subsets comparing **C** PRCV-infected or **D p**H1N1-infected pigs with control pigs, based on genes list sorted by logFC values. Bubble sizes represent the ratio of the number of DE genes associated with a specific KEGG pathway to the total number of DE genes identified in that cluster. Bubble colours represent the adjusted P-values.

To compare transcriptional changes during PRCV and pH1N1 infection, we performed differential expression (DE) analysis within each annotated cluster using edgeR. Few genes showed DE at 1 DPI in either infection (Supplementary Fig. 13B). However, comparing each infection group with the controls presented more extensive DE across multiple cell types (Supplementary Figs. 14 and S15, S16) and convergent transcriptional changes in response to the two viruses. For example, in a population of interstitial macrophages, both infections upregulated *DCTN4*, encoding a dynactin complex component involved in endosomal trafficking, and *SLC15A4*, encoding a lysosomal proton-coupled peptide transporter required for TLR7/9 signalling, suggesting enhanced vesicular transport and innate immune signalling (Supplementary Fig. 14C)^30^. While these changes in innate signalling in macrophages are to be expected, we also found that in B cells both infections upregulated an antiviral program. Within B cell cluster 10, induction of interferon-stimulated genes (ISGs) such as *IFI44*, *HERC5*, *IRF9*, *UNC93B1*, *OAS2* and *PARP11* was observed (Fig. 5A). In plasma cells (cluster 2) pH1N1 infection upregulated classical interferon-stimulated genes (ISGs) including *ISG15*, *ISG20*, *OASL*, *OAS2, MX2, HERC5*, and *EIF2AK2*, compared to controls. In both infections, there was an upregulation of UBE2L6, encoding an interferon-stimulated E2 enzyme that mediates ISG15 conjugation. In contrast, inhibitory regulators of B cell activation and antibody production, such as *TGFBR2* and *ZBTB10*, were downregulated (Fig. 5B). Following this, virus versus control DE results from all lymphoid compartment clusters were subjected to gene set enrichment analysis (GSEA) using gene sets from the Kyoto Encyclopaedia of Genes and Genomes (KEGG) database. This showed enrichment of several disease related pathways in one T cell cluster, annotated as activated T cells (cluster 3re), suggesting energy metabolism and stress-related signalling for both viruses compared to the control condition (Fig. 5C, D). Similarly, cytotoxic T cells (cluster 1re) showed enrichment of metabolic pathways in both infections (Fig. 5C, D). This highlighted that alongside myeloid cells, local cells of the adaptive immune system also respond as early as 1 DPI to these viral infections.

In the non-immune compartment, direct comparisons between PRCV and pH1N1 revealed no DE genes at 1 DPI. However, compared with controls, both infections induced robust interferon-driven and extracellular matrix (ECM) remodelling programmes in capillary endothelial cells (cluster 13), with upregulation of *MX1, RSAD2, PARP9, FOXP1, FOXS1, GATA2, COL8A1*, and *SULF1*, alongside downregulation of genes for angiogenic and barrier-stabilising factors such as *ROBO4, PLVAP*, the *VEGFR* gene *KDR, IL6, CCL19*, and *CCL2* (Supplementary Fig. 16C). This illustrated synergistic gene expression changes even in non-immune cells during both infections.

### Differing PRCV and pH1N1 infection immune signatures at 12 DPI

**Fig. 6:**
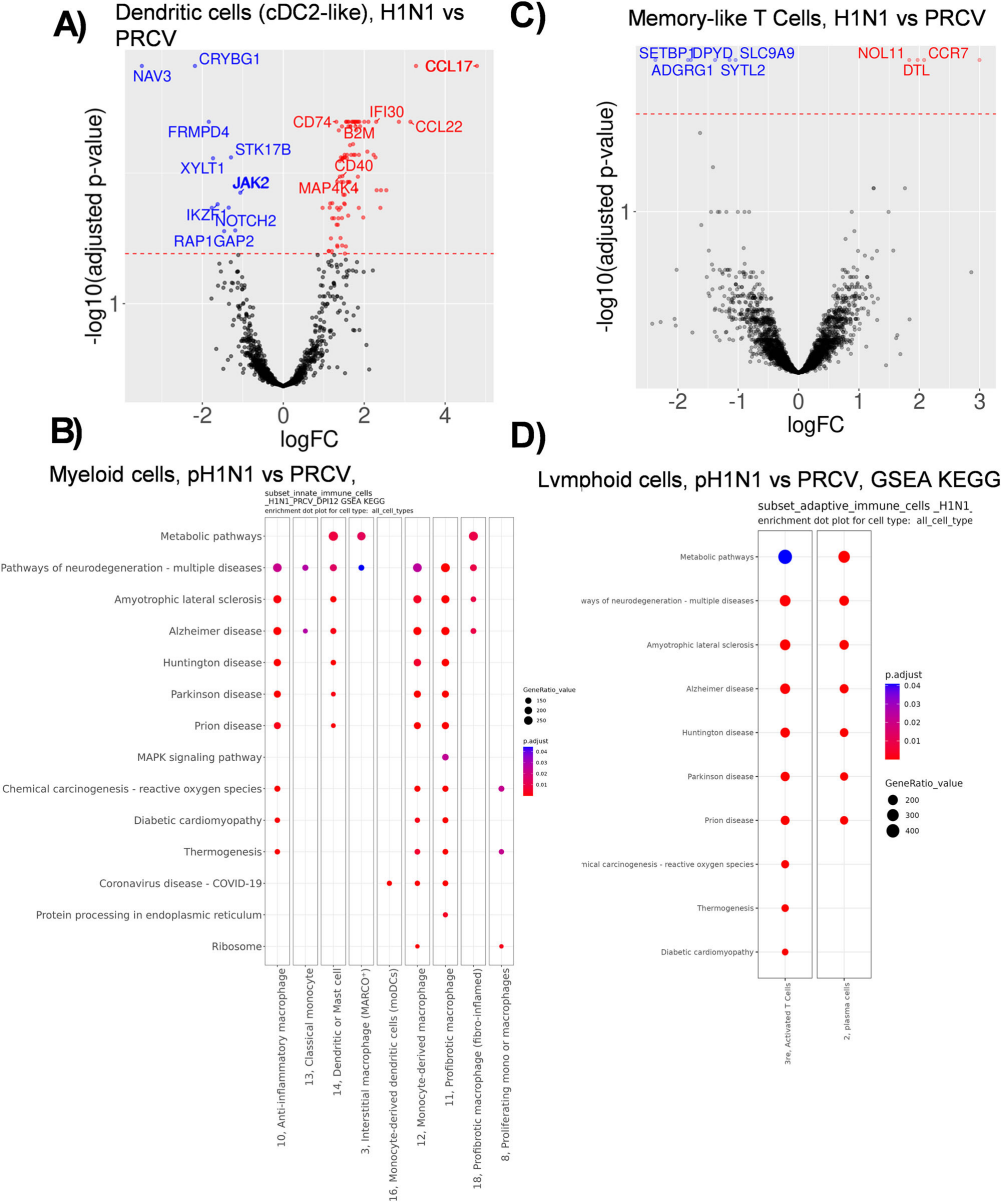
Myeloid and adaptive immune cells show heightened transcriptional changes after PRCV infection at 12 DPI. **A** A volcano plot of dendritic cell cluster 2 for PRCV vs pH1N1 at 12 DPI. **B** Bubble plot representing enriched KEGG pathways across myeloid cell subsets comparing PRCV with pH1N1 based on genes list sorted by logFC values. Bubble sizes represent the ratio of the number of DE genes associated with a specific KEGG pathway to the total number of DE genes identified in that cluster. Bubble colours represent the FDR adjusted *P*-values. **C** A volcano plot of memory-like T cell cluster 2re for PRCV vs pH1N1 at 12 DPI. **D** Bubble plot as in (**B**) but for lymphoid cells.

Transcriptional changes between the two infections became apparent by 12 DPI. These were primarily located within specific cell subsets such as dendritic cells (Supplementary Fig. 17A) and a T cell subcluster (cluster 2re, Supplementary Fig. 17B). PRCV-infected dendritic cells exhibited higher expression of genes associated with antigen presentation and immune activation relative to pH1N1-infected ones, including *CD74, B2M, IFI30*, and *CD40*, as well as the chemokines *CCL17* and *CCL22*, which mediate lymphocyte recruitment. Higher expression of ribosomal RPS and RPL genes pointed to enhanced protein synthesis, and greater expression of *MAP4K4* suggested activation of stress-responsive and pro-inflammatory signalling pathways (Fig. 6A). At the same time, lower expression of *JAK2* and *IKZF1* indicated altered cytokine responsiveness and a potential shift in dendritic cell maturation dynamics. KEGG GSEA across all myeloid subsets indicated enrichment of gene sets associated with heightened transcriptional activity and several disease categories, including COVID-19 (Fig. 6B).

Within the lymphoid compartment, PRCV-infected pigs displayed distinct remodelling of memory-like T cells (cluster 2re), including higher expression of DTL (encoding a regulator of DNA replication and cell cycle progression), *CCR7* and *NOL11* (encoding a nucleolar protein essential for ribosome biogenesis and proliferation). This suggested a shift toward a central memory-like or migratory phenotype. In contrast, *SETBP1, SLC9A9, ADGRG1/GPR56, SYTL2*, and *DPYD* were less expressed, pointing to a diminished proliferative, cytotoxic, and metabolic potential compared with pH1N1 (Fig. 6C). KEGG GSEA across lymphoid subsets showed enrichment of disease-related pathways in activated T cells (3re), indicating sustained metabolic and stress-response activity in PRCV-infected pigs (Fig. 6D). The same was found for cluster 2, representing plasma cells (Fig. 6D). In the non-immune compartment, direct comparisons between PRCV and pH1N1 revealed no obvious differences at 12 DPI (Supplementary Fig. 17C).

In summary, scRNA-seq analysis revealed that both PRCV and pH1N1 infections triggered early, broad activation of innate and adaptive immune pathways, in which macrophages, B cells, and T cells exhibited convergent antiviral and metabolic responses by 1 DPI. Transcriptional profiles diverged by 12 DPI, particularly in dendritic cells and differentiated T and B cells, indicating virus-specific patterns of immune modulation and recovery.

### Pig nasal microbiome profiling in response to viral infection

**Fig. 7:**
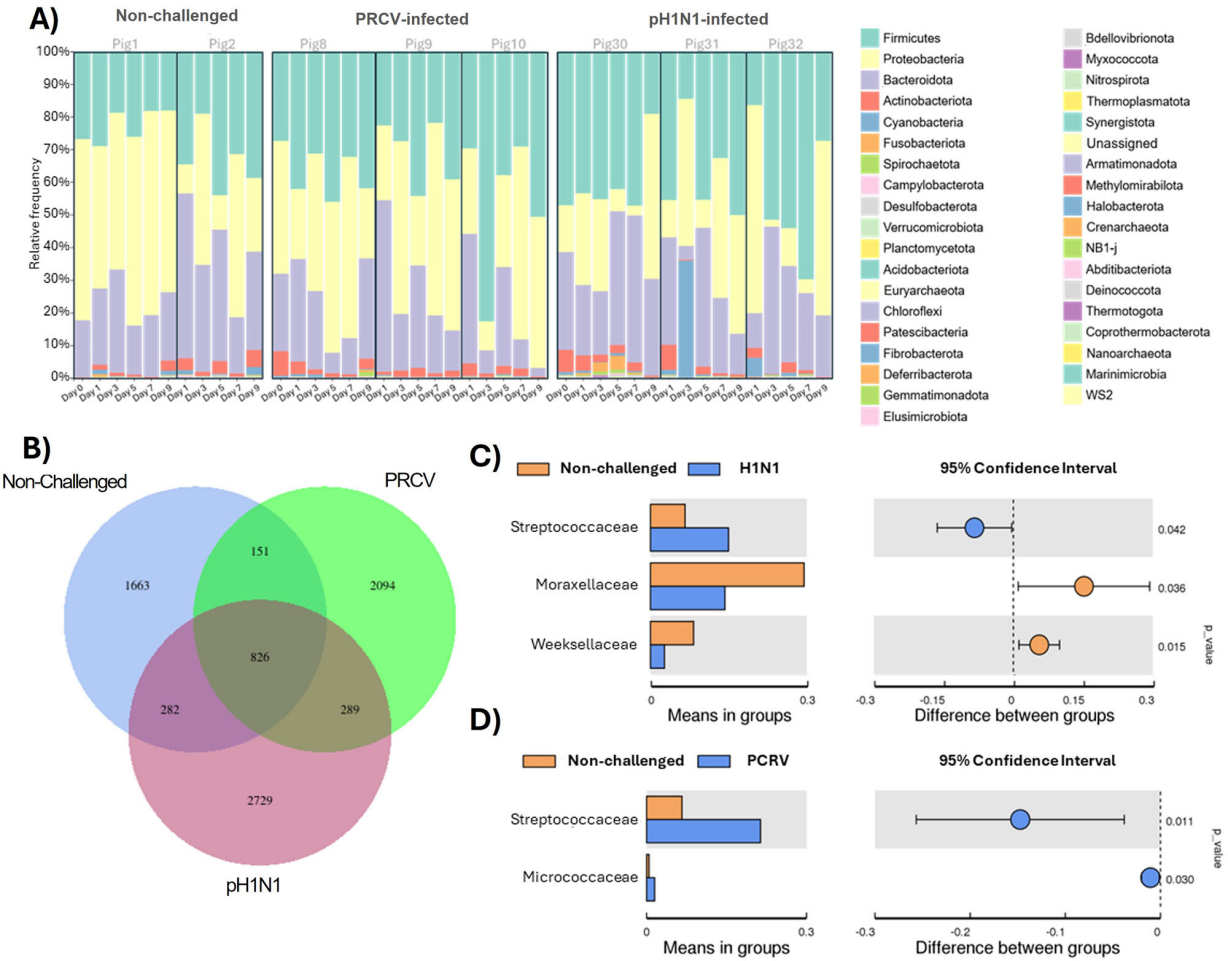
Nasal microbiome profiling of pigs in response to viral challenge. **A** Taxonomic bar plots showing the top phyla associated with nasal microbiota samples from non-challenged, PRCV-infected, and pH1N1-infected pigs. **B** Venn diagram depicting the number of unique and shared ASVs between non-challenged pigs, PRCV-infected pigs, and pH1N1-infected pigs **C** Taxonomic families that are significantly (*p*-value < 0.05) enriched or decreased in pH1N1-infected pigs (blue bars) relative to non-challenged pigs (orange bars). Each bar represents the mean value of the abundance in each group of the bacterial family, showing significant differences between groups. The left panel is the abundance of each bacterial family. The right panel is the confidential interval of between-group variations. **D** Taxonomic families that are significantly (*p*-value < 0.05) enriched or decreased in PRCV-infected pigs (blue bars) relative to non-challenged pigs (orange bars).

The composition of the nasal microbiota in pigs subjected to either PRCV or H1N1 infection was assessed over a nine-day period (Fig. 7A). Comparative analysis revealed 2094 amplicon sequence variants (ASVs) uniquely associated with PRCV-infected animals, and 2729 ASVs were specific to the H1N1 infected group. In contrast, 1663 ASVs were exclusive to non-challenged controls. A core nasal microbiome, comprising 826 ASVs, was shared across all experimental groups, and an additional 289 ASVs were found exclusively in the virally infected pigs (Fig. 7B).

Taxonomic profiling indicated a significant enrichment of *Streptococcaceae* in both pH1N1- (*p* < 0.05) and PRCV-infected pigs (*p* < 0.02). Conversely, *Moraxellaceae* and *Weeksellaceae* were notably reduced in the pH1N1 cohort, while *Micrococaceae* were significantly enriched in the PRCV-infected group (Fig. 7C, D and Supplementary Fig. 18). Longitudinal analysis further demonstrated an increase in alpha diversity in H1N1-infected pigs by 5 DPI, surpassing that observed in both the PRCV-infected and control groups. This elevated diversity correlates with the higher number of unique ASVs identified within the pH1N1 cohort.

In summary, microbiome profiling of porcine nasal swabs revealed both shared and virus-specific alterations in bacterial communities following PRCV and pH1N1 infections. PRCV and pH1N1 infections each led to distinct microbial community shifts, PRCV associated with *Micrococaceae* enrichment and pH1N1 with broader bacterial diversity changes. These results highlight the differential impact that PRCV and pH1N1 viruses have on the upper porcine airway microbiota.

## Discussion

Using the porcine model, which closely resembles the human respiratory and immune systems, this study provides a controlled comparison of PRCV and influenza pH1N1 infections to dissect virus-specific determinants of host response. PRCV infection induced prolonged nasal viral shedding and higher viral load in BAL and lung than pH1N1. This enhanced replication was associated with more severe lung pathology and broader involvement of the upper respiratory tract compared to pH1N1.

The higher viral load and prolonged shedding observed may reflect the greater replication efficiency of PRCV, which, like other coronaviruses, replicates exclusively in the cytoplasm. In contrast, influenza virus replication requires nuclear import and export of the viral ribonucleoprotein complex, a process that may impose additional constraints on replication kinetics (29,30). Human pH1N1 infection is also associated with a shedding duration of approximately 3–8 days, similar to that observed in pigs^31^. Comparisons between human SARS-CoV-2 and influenza virus infections further demonstrate that SARS-CoV-2 exhibits longer transmission windows and extended shedding durations than influenza viruses, mainly due to differences in tissue tropism, host–virus interactions, and immune evasion strategies^32^. Similarly, differences in viral tropism between PRCV and pH1N1 are likely influenced by the distribution of their respective entry receptors within the porcine respiratory tract. PRCV utilises aminopeptidase N (APN/CD13) as its cellular receptor^33^. APN gene and protein expression have been detected in epithelial and sub-epithelial regions of the porcine respiratory tract, including the trachea, bronchioles, and lung^19,34^. Consistent with this distribution, PRCV has been reported to infect bronchiolar epithelial cells, type I and type II pneumocytes, and septal macrophages^35^.

Influenza viruses bind to α2,3- and α2,6-linked s ialic acids (33), which display a broadly similar distribution in pigs and humans, with α2,6-linked sialic acids expressed in the upper respiratory tract, while the lower respiratory tract contains a mixture of α2,3- and α2,6-linked sialic acids^36–38^. However, α2,3-linked sialic acids have been reported at higher concentrations in the porcine nasal mucosa compared with humans. While receptor distribution is an important determinant of infection site and downstream immune signalling, detailed spatial quantification of receptor abundance at localised sites of infection was beyond the scope of the present study. Instead, we focused on characterising immune responses, recognising that differences in receptor usage and viral tropism are intrinsic features of these models and likely contribute to the distinct host responses observed.

Interestingly, despite the more extensive lung pathology observed in the PRCV infection, immunohistochemical analysis in this study revealed greater local infiltration of CD163⁺ macrophages and CD3⁺ T cells in pH1N1-infected lung lesions at the early stages of the infection. This pattern suggests more immune cell recruitment to discrete lesion sites during pH1N1 infection, which may promote faster viral clearance and tissue repair, whereas PRCV infection was associated with more severe widespread pathology and less focal immune infiltration.

While both influenza and PRCV induced serum and BAL antibody responses, pH1N1 elicited neutralising antibodies as early as 5 DPI, with significantly higher titres throughout infection. This rapid humoral response coincided with increased frequencies of HA-specific plasmablast/plasma cells in BAL and elevated Tfh responses in TBLN, consistent with earlier reports demonstrating the importance of Tfh cells in influenza immunity^39,40^. In contrast, PRCV infection was associated with more robust expansion of spike-specific B cells across systemic and mucosal compartments, suggesting greater priming of long-lived memory. These findings imply that PRCV may preferentially drive memory B cell differentiation rather than immediate antibody neutralisation, potentially contributing to prolonged viral persistence. These observations agree with studies of influenza and CoV infections, where primary influenza A virus infection has been shown to rapidly establish HA-specific germinal centre and memory B-cell pools within two weeks, accompanied by robust Tfh support and early plasmablast differentiation^41,42^. By contrast, CoV infections, including SARS-CoV-2, induce Spike-specific memory B cells more gradually, with airway and circulating pools typically emerging after 2-3 weeks and undergoing continued clonal evolution over months^43–45^.

Cytokine T cell analysis demonstrated that PRCV infection induced greater cytokine-producing T cell response than pH1N1 in BAL and PBMC. In the BAL, there were higher frequencies of IFNγ⁺TNF⁺ double-producing CD4⁺, CD8⁺, and γδ T cells, coupled with effector memory phenotypes indicative of recent activation. In PBMC, PRCV also induced strong IFNγ and TNF production after antigenic restimulation, with enrichment of central memory CD4⁺ T cells. Although IFNγ and TNF play critical roles in viral control, their excessive production can contribute to immunopathology, as reported for both human coronavirus and influenza infections^46–49^. Heightened T-cell activation and hypercytokinemia have been associated with severe COVID-19 and influenza disease^46,50,51^. Increased cytokine secreting CD4 T cells in PRCV infected PBMC were associated with increased proportions of TCM with elevated expression of activation markers compared to pH1N1 infection which the T cell response was mainly limited to BAL. Consistent with this observation, an increase in virus specific CD4 TCM in blood has been reported in humans following SARS-CoV-1 infection^52^. Whether the increased cytokine response during PRCV infection primarily contributes to lung pathology or reflects a protective mechanism to restrict viral replication remains to be determined.

The systemic activation of T cells during PRCV infection occurred in parallel with an expansion of Tregs, suggesting a balanced immune program that integrates effector and regulatory pathways to prevent uncontrolled inflammation. In contrast, the T cell response to pH1N1 was more compartmentalized within the respiratory tract, which may aid local viral clearance but limit systemic memory generation. These divergent patterns of T cell immunity between PRCV and pH1N1 may reflect differences in viral tropism, antigen load, or receptor usage, which ultimately shape the distribution and magnitude of T cell responses.

At the early time points (DPI 1), both PRCV and pH1N1 infection triggered a conserved interferon-driven program across immune and stromal cell compartments. This was reflected by induction of canonical ISGs, including *MX1/MX2, ISG15, OAS* family members, *DDX60*, and *HERC5*, which collectively restrict viral replication through capsid blockade, ISGylation, RNA sensing, and RNA decay mechanisms. This broad antiviral signature likely underlies the absence of transcriptional differences between PRCV and pH1N1 at 1 DPI, despite robust reprogramming across myeloid, B cell, and T cell compartments. Similar signatures were found in studies with human lung cells. For example, interferon type-I and type-III driven gene programmes were identified in lung and upper airway cell preparations of COVID-19 patients sampled in several countries^53,54^. Human scRNA-seq datasets further identify interferon-activated myeloid cell states that closely mirror the PRCV-induced clusters observed in pigs^23,55^. Similarly, human airway epithelial cells also responded by type-I and type-III interferon driven transcriptional signatures following exposure to pH1N1^56^. In myeloid cells (interstitial macrophages), the coordinated upregulation of vesicular trafficking and lysosomal signal, and *SLC15A4*, a proton-coupled peptide transporter required for optimal TLR7/9 signalling^57^ suggested sustained antigen processing and endosomal TLR licensing in PRCV-infected lungs, while pH1N1 infection skewed toward lymphoid expansion. Interestingly, similar pronounced myeloid cell reprogramming, with enhanced antigen presentation machinery (e.g., *CD74, B2M*) and IFN-γ–associated macrophage activation states, have also been reported in human SARS-CoV-2 infected lung tissue from Malawian COVID-19 patients^54^. In dendritic cells, PRCV induced CD40 and MHC-associated transcripts, together with *CCL17* and *CCL22*, consistent with enhanced T-cell priming potential, whereas downregulation of *JAK2* and *IKZF1* indicated altered maturation and cytokine responsiveness.

Endothelial remodelling was a shared feature of both infections, with downregulation of angiogenic and barrier-stabilizing factors such as *ROBO4*^58^ and upregulation of permeability-associated *PLVAP*^59^. In PRCV, additional induction of stress-adaptation genes (*HSP70, TXNIP, DNAJB1*) and connexins (*GJA5*) suggested heightened oxidative stress responses and altered endothelial activation^60^. While these effects were transient, suggesting reduced barrier stability in the very early phases of infection with the two viruses in our pig model, similar effects have been reported in human patients with severe COVID-19^61^, which were also associated with co-morbidities^62^. Together, these data support a model in which PRCV sustained antigen presentation and stress-adaptation circuits, accompanied by central memory-like T cell remodelling, while both infections altered transiently barrier integrity.

Comparative scRNA-seq analyses indicate that porcine and human lung immune landscapes are broadly conserved, with substantial overlap of lymphocyte subsets^12,63^. Although the porcine immunome shows strong conservation of innate and adaptive immune pathways, some differences, including an abundance of γδ T cells, expansions of selected innate immune gene families, and divergence in transcriptional programs, likely reflect evolutionary adaptation to distinct pathogen pressures and should be considered when translating porcine immune findings to humans^21^. Consistent with this, comparative analysis of conserved antiviral genes revealed broadly similar antiviral signatures across NK and cytotoxic T-cell populations in pigs, mice, and humans, despite higher expression of several antiviral genes in human cells^21^. Together, these findings support the relevance of the pig as a model for human pulmonary immunity while indicating species-specific features and technical factors that warrant careful interpretation and validation across additional datasets.

We included longitudinal nasal microbiome profiling in this study to determine whether the distinct patterns of viral replication, lung pathology, and immune activation seen with PRCV and pH1N1 were accompanied by virus-specific alterations in the upper airway microbial community, which is increasingly recognised as a modulator and potential biomarker of respiratory disease outcomes. Microbiome profiling of nasal swabs revealed both shared and specific alternations in bacterial communities following PRCV and pH1N1 infections. There was enrichment of *Streptococcaceae* in both PRCV and pH1N1 infection, a family that includes opportunistic pathogens both in human and swine, such as *Streptococcus suis* (in pigs) and *Streptococcus pneumonia* (in humans). In virus specific-microbial alteration, PRCV infection was associated with enrichment of *Micrococcaceae*, a family that is typically part of the skin and mucosal flora, but has been reported as a secondary infection during COVID-19 (e.g., *Staphylococcus aureus* co-infection)^64–67^; thus, elevated *Micrococcaceae* numbers in PRCV infection potentially reflected the greater epithelial disruption and lung pathology in this group. On the other hand, pH1N1 infection resulted in a reduction in *Moraxellaceae* and *Weeksellaceae*, families commonly associated with respiratory tract homeostasis, suggesting that influenza may alter the nasal microenvironment even without extensive lung pathology^68^. The enrichment of *Streptococcaceae* we observed in our porcine model study aligns with observations from human influenza and COVID-19 cohorts, in which expansion of bacterial pathobionts and disruption of commensal communities have been associated with more severe disease and an increased risk of bacterial superinfection^68,69^. Collectively, 289 ASVs were associated with virally infected pigs, representing potential infection biomarkers.

These findings highlight that while certain nasal microbiota shifts, such as *Streptococcaceae* expansion, are broadly associated with respiratory viral infection, others are pathogen-specific and may both influence and reflect the degree of mucosal damage, inflammation, and risk of secondary bacterial infections. Targeted follow-up studies are required to elucidate the functional basis of these associations and the impact on virus transmission.

Our data demonstrate that PRCV and pH1N1, despite both being respiratory RNA viruses infecting the same host, engage the immune system in different ways. PRCV induced higher viral load and prolonged viral shedding, stronger systemic and mucosal T cell activation, expansion of memory B cells, and distinct microbiome shifts. In contrast, pH1N1 resulted in rapid neutralising antibody production, robust Tfh and germinal centre B cell responses, and broader early airway microbial diversity changes. Transcriptional responses to PRCV and pH1N1 infection started with the activation of shared interferon-stimulated genes but later diverged as pathways involving stromal-immune interactions and vascular integrity started to shape lung pathology and subsequent immune responses. These findings highlight the importance of pathogen-specific immune signatures in shaping acute outcomes and long-term immunity and suggest that optimal vaccine or therapeutic strategies need to consider these differences.

## Materials and methods

### Viruses

In this study, the porcine respiratory coronavirus (PRCV/swine/Belgium/PS-071/2020 (PRCV) strain and influenza A virus (A/swine/Gent/53/2019 (pH1N1) strain were used^70^. PRCV and pH1N1pdm09 viruses were grown and quantified using swine testis (ST) cells and Madin-Darby Canine Kidney (MDCK, ATCC) cells, respectively.

### Ethics statement

Animal experiments were approved by the ethical review processes of The Pirbright Institute and the Animal according to the UK Animals (Scientific Procedures) Act 1986 under project licenses PP7764821 and PP2064443. The Pirbright Institute conforms to Animal Research: Reporting of Animal Experiments guidelines. We have complied with all relevant ethical regulations for animal use. Animals were acclimatized for at least 7 days and were randomized into different groups and pens using Excel by the animal services staff. Researchers processing the samples were only aware of the pig numbers, not the group assignments. The pathologists were blinded to the group allocation when assessing the samples for gross pathology during *post-mortem* examination and subsequently during the histopathological assessment. All conditions were kept the same between the groups and full environmental enrichment was provided to all animals.

### Animal study

A total of 36 outbred female pigs, 50% Landrace and 50% large white, 5–7 weeks of age, were sourced from Home-PIC UK. The pigs were Porcine Reproductive and Respiratory Syndrome virus and Mycoplasma hyopneumoniae negative. The pigs were screened by ELISA or HAI for the absence of serum antibodies against the spike of PRCV, HA of H1N1pdm09, H1N2, H3N2, and avian-like H1N1 and were randomly divided into 3 groups. The group size of five pigs was determined based on previous experience demonstrating reproducibility of virological, serological, immunological, and histopathological parameters^18,26,71,72^. The first group was infected with 8 × 10^6^ PFU of PRCV (*n* = 15) (1 ml per nostril) administered intranasally using a mucosal atomization device (MAD) following sedation with a 3 mg kg^−1^ Zoletil and 1.5 mg kg^−1^ Stresnil. The second group was inoculated with 8 × 10^6^ PFU of pH1N1 (*n* = 15) by the same route. The third group, serving as control animals (*n* = 6) received PBS intranasally (1 ml per nostril). Clinical parameters, including demeanour, appetite, respiratory signs (sneezing and coughing), nasal and ocular discharge, faecal consistency, and rectal temperature, were evaluated. No remarkable clinical signs were observed, apart from two pH1N1 infected pigs with diarrhoea for two days from 5 to 8 DPI. End points were specified in project licenses PP7764821 and PP2064443 based on pyrexia, behaviour, anorexia, and signs affecting the digestive and respiratory systems. Virus inoculum used for inoculation of the animals was backtitered to confirm that an equivalent viral load was delivered to each group of animals. We deliberately chose to use an identical inoculum at the same dose for both viruses rather than titrate doses to match lung pathology. Consistency and reproducibility at the point of inoculation reflect more accurately differences in viral behaviour and host response. This approach resulted in lung pathology similar to that in humans, further supporting the use this strategy.

Five pigs from PRCV and pH1N1 groups and two animals from the control group were humanely culled with an overdose of pentobarbital sodium anaesthetic, confirmed by the permanent cessation of circulation, on 1, 5, and 12 days post-infection (DPI) to assess lung pathology, viral load and immune responses. Daily nasal swabs were collected from all animals and placed in virus transport media (VTM) to quantitate virus shedding and for microbiome profiling. Following necropsy, bronchoalveolar lavage (BAL) fluid and peripheral blood mononuclear cells (PBMC) were collected for assessment of immune responses. BAL fluid and homogenised accessory lung lobes were used to assess viral load by plaque assay. No animals were excluded from the analyses.

### Respiratory pathology assessment

At necropsy, dorsal and ventral surfaces of animals’ lung were examined, and pulmonary lesions were scored as previously described^73^ by a qualified veterinary pathologist. Briefly, macroscopic lung lesions were given a score to estimate the percentage of the lung affected by pneumonia, with a maximum score of 100. Lung samples from the right cranial, medial and caudal lobes were fixed by immersion in 10% neutral buffered formalin (NBF) and processed routinely for histopathological analysis. Haematoxylin and eosin (H&E) staining and immunohistochemistry (IHC), targeting PRCV nucleocapsid (N) and pH1N1 nucleoprotein (NP), were performed on serial tissue sections as previously described^23,26,71^.

For immunohistochemical staining, A Leica BOND-RXm (Leica Microsystems) was used, applying a BOND Epitope Retrieval Solution 1 (ER1, pH 6.0) for 20 min at 95 °C as antigen retrieval. An anti-influenza NP monoclonal antibody (HB-65) as hybridoma culture supernatant was used at 1:3 dilution; or an anti-PRCV nucleoprotein (Clone DA3) diluted at 1:10,000 was incubated for 30 min. Leica polymer refine detection kit (Leica Microsystems) and DAB were used to visualize the staining. Finally, sections were counterstained with Harris’ haematoxylin and routinely dehydrated and mounted using the Dako mounting medium (Agilent, Santa Clara, USA). Negative controls, consisting of replacement of primary antibody by blocking solution (OMIT), were included in each run to detect potential non-specific binding. Positive control tissues were also included in each IHC run. H&E and IHC-stanied sections were scanned with a Hamamatsu S360 digital scanner and evaluated with ndp.view2 software (Hamamatsu, Tokyo, Japan). Two different scoring systems were used to evaluate and quantify the histopathological lesions on the lung, previously described by Morgan et al., in 2016 (“Morgan”)^71^ and by Gauger et al., in 2012 (“Iowa”)^28^. In the “Morgan” scoring system, histopathological changes were scored using 5 parameters: (1) airway epithelial necrosis, attenuation or disruption, (2) airway inflammation, (3) perivascular/bronchiolar lymphocytic cuffing, (4) alveolar cellular exudate/oedema and interlobular oedema and (5) alveolar septal inflammation. Each parameter was scored as follows: 0 (none), 1 (minimal), 2 (mild), 3 (moderate) and 4 (severe) For the “Iowa” scoring system, 4 histopathological parameters were evaluated: (1) necrotising bronchitis/bronchiolitis, (2) suppurative bronchitis/bronchiolitis, (3) peribronchiolar lymphocytic cuffing and (4) alveolar septal inflammation scored from 0 (none) to 3 (marked/severe). Additionally, in this scoring system, the presence of virus antigen (NP) by IHC was also evaluated semi quantitatively in (a) the airway epithelia and (b) the alveoli lumen/septum. A cumulative score for the three lung lobes was calculated for each scoring system and animal^28^.

### Multiplex (mIHC) and single-plex immunohistochemistry

A multiplex immunohistochemistry (mIHC) technique was developed to study the colocalisation of influenza virus NP or porcine respiratory coronavirus (PRCV) N within epithelial cells (Pan-cytokeratin+) or macrophages (Iba1+). For this purpose, 10 lung samples from each infected group with marked histopathological lesions and high expression of viral antigen were selected and sectioned at 4 μm. Three samples were selected from each group, euthanized at 1 DPI, four at 5 DPI and three at 12 DPI. Additionally, 2 lung samples from non-infected control animals euthanized at 5 DPI were included. Sections were stained using the Opal 3-Plex Detection Kit (formerly Opal 4-Color Automation IHC Kit) (Akoya Biosciences, MA, USA, cat No. NEL820001KT) in the Leica BOND-RX (Leica Microsystems, Milton Keynes, United Kingdom). Tissue sections were stained with a 4-plex protocol (PRCV N or Influenza NP, Pan-cytokeratin, Iba1 and DAPI). A summary of the mIHC methodology and the Opal fluorochrome assigned to each primary antibody is presented in Table 1. Stained slides were scanned using the multispectral imaging system of the PhenoImager® HT (Akoya Biosciences), and image acquisition was performed with Phenochart™ software (Akoya Biosciences).

**Table 1:** Summary of multiplex immunohistochemistry (mIHC) techniques: primary antibody and Opal tag details

Primary antibody	Type of antibody	Source	Dilution	Antigen retrieval	Opal	Opal dilution
**Anti-PRCV**	Monoclonal (clone DA-3)	In house	1:10000	ER2 20 min^a^	Opal 690 (red)	1:300
**Nucleoprotein**						
**Anti-influenza**	Polyclonal	Thermo Fisher Scientific	1:4000	ER2 20 min^a^	Opal 690 (red)	1:150
**Nucleoprotein**						
**Pan-cytokeratin**	Monoclonal (clone PCK-26)	Novus Biotechne, Oxford, UK	1:100	ER1 20 min^b^	Opal 620 (yellow)	1:100
**Iba1**	Polyclonal	FujiFilm Wako, Neuss, Germany	1:100	ER1 20 min^b^	Opal 520 (green)	1:300
***-***	-	-	-	ER2 20 min^a^	DAPI (blue)	1:10

PRCV, porcine respiratory coronavirus.

^a^ER2, BOND heat-induced epitope retrieval solution 2 (pH 9.0).

^b^BOND heat-induced epitope retrieval solution 1 (pH 6.0).

Additionally, sequential sections from these selected samples were stained by immunohistochemistry (IHC) using antibodies against CD163 (macrophages), CD3 (T cells), CD20 (B cells) and calprotectin (MAC387, expressed by neutrophils and some activated macrophages). The avidin-biotin-peroxidase complex technique (ABC Vector Elite, Vector Laboratories, Burlingame, CA, USA) was used as previously described^74^. Briefly, tissue sections were deparaffinised and dehydrated through a graded series of alcohol, followed by blocking of the endogenous peroxidase activity using 3% hydrogen peroxide in methanol for 30 min in darkness. Primary antibodies (monoclonal anti-human calprotectin Dako/Agilent 1:100; monoclonal anti-human CD163, Bio-Rad 1:300; polyclonal anti-human CD3 Dako/Agilent 1:100; polyclonal anti-human CD20 Epredia 1:100) were incubated overnight at 4 °C after epitope demasking technique (incubation in citrate pH 6.0 buffer for 30 min at 95 °C or protease enzymatic digestion). Biotinylated anti-mouse secondary antibody was applied for 30 min followed by the avidin-peroxidase compelx (Vector Laboratories) following the manufacturer’s instructions (Vector Laboratories). Labelling was visualized by NovaRED substrate kit (Vectore Laboratories). These single-plex immunostained sections were digitally scanned with the Hamamatsu S360 digital slide scanner (Hamamatsu Photonics K.K). In order to quantify the expression of each cell marker, digital images were evaluated with the Nikon NIS-Ar software (Nikon Instruments Inc., NY, USA) to calculate the percentage of positivity (percentage area positively stained) in the whole sections and using ROIs (“lesion”) selecting only areas showing broncho-interstitial pneumonia, if present^75^.

### Virological assays

The evaluation of infectious viral progeny was performed using BAL fluid in PBS and daily nasal swabs in VTM collected throughout the study. Samples from PRCV- and pH1N1-infected and control animals were subjected to plaque assays using ST (ATCC CRL-1746) and MDCK (ATCC CCL-34) cells, respectively, according to established protocols^18,71^. For PRCV plaque assays, ST cells were seeded into 12-well plates and allowed to reach confluence. Prior to infection, the cells were washed with PBS. Each well was then inoculated with 250 µL of virus, performed in duplicate or triplicate. After a 1-h incubation at 37 °C, the inoculum was removed and replaced with 2 mL of overlay medium containing N,N-bis(2-hydroxyethyl)-2-aminoethanesulfonic acid and 1% agar (cell culture grade; Sigma). Following 96 h of incubation, plaques were visualised by staining with 0.1% crystal violet (in water) after fixation with 3.3% formaldehyde in PBS. PFU per mL were then calculated. For pH1N1 plaque assays, viral titres in nasal swabs, lung homogenates and BAL were quantified using MDCK cells. Samples were serially diluted 10-fold, and 100 µL of each dilution was added to confluent MDCK monolayers in 12-well plates. After a 1-h adsorption period, the cells were washed and overlaid with 2 mL of a 2% agarose/medium mixture (1:3). Plates were incubated at 37 °C for 48 h, after which plaques were visualised using 0.1% crystal violet.

Lung tissue collected postmortem was homogenized in a 1:1 ratio with Dulbecco’s Modified Eagle’s Medium (DMEM, Gibco™) and centrifuged at low speed to remove debris. The resulting supernatants from PRCV- and pH1N1-infected lungs were then titrated using plaque assays on ST and MDCK cells, respectively.

### Serological assays

Maxisorp 96-well plates (ThermoScientific) were coated with 100 µl/well of 0.5 µg ml^−1^ recombinant PRCV spike protein from ISU-1 or PRCV (see below) or 2 µg ml^−1^ recombinant HA1 diluted in PBS overnight at 4 °C^17,18,23^. The next morning, coating buffer was washed off with wash buffer (PBS containing 0.05% Tween20) and blocked for 1 h at room temperature with block buffer (wash buffer containing 4% w/v Marvel milk powder). Samples were serially diluted 2-fold in block buffer starting at 1:5 (BAL) or 1:20 (serum) and 100 µl/well applied to the plate for 1 h at room temperature (serum) or 16 h at 4 °C. Sample was washed off the plate, then 100 µl/well of secondary antibody (anti-porcine IgG H&L-HRP or anti-porcine IgA-HRP, Bio-Rad) diluted 1:20,000 in block buffer added for 1 h at room temperature. The assay was developed using 50 µl/well of TMB high-sensitivity substrate solution (Biolegend), and the reaction terminated using equal volume of 1 M sulphuric acid. Optical density at 450 nm and 630 nm was measured, and endpoint titre determined.

### Generation of recombinant soluble (biotinylated) PRCV spike

Human codon-optimized cDNA encoding the ectodomain of PRCV/swine/Belgium/PS-071/2020 spike (GenScript) was cloned in frame with sequences encoding a GCN4-isoleucine-zipper trimerization motif^75^, a tandem repeat of a biotinylation acceptor peptide^76^ and a double Strep tag into a pCAGGS expression plasmid between the EcoRI-NotI restriction sites using the HiFi DNA Assembly kit (New England Biolabs). [Xd1.1]To stabilize the prefusion conformation of the spike protein two proline substitutions at positions 913 and 914 were introduced^77^. The plasmid (Genbank NCBI: PZ305746) was transiently transfected into Freestyle 293-F cells (Gibco) that were maintained in Freestyle 293 expression medium (37 °C, 130 rpm) using polyethylenimine (1 mg/mL, PolyScience). The transfected cultures were supplemented with 10% Peptone Primatone (Sigma) and 300 mM Valporic acid sodium salt (Sigma) 24 h post transfection. After 5 days, secreted proteins were purified from supernatants using strep-tactin sepharose beads (IBA Lifesciences) and Poly-Prep Chromatography columns (Bio-Rad Laboratories) following the manufacturer’s protocol. The purified proteins were eluted in the following buffer (pH8): 100 mM Tris-HCL, 150 mM NaCl and 2.5 mM D-Biotin (Sigma). The eluted proteins were quantified using quantitative densitometry of GelCode Blue (Thermo Fisher Scientific)-stained protein 10% gels, which also contained bovine serum albumin (BSA) standards, and the generated images were analyzed with an Odyssey imaging system (LI-COR). PRCV spike protein was biotinylated using an enzymatic protein biotinylating kit (Merck) according to the manufacturer’s instructions.

### Virus neutralisation assay

Neutralisation of PRCV and pH1N1 was performed in parallel using a 96well format. Serum samples were heat-inactivated at 56 °C for 30 min, then two-fold serial dilutions were prepared in the appropriate growth medium. For PRCV assays, each dilution was mixed with 1 × 10³ PFU of PRCV and incubated for 30 min at room temperature before overlaying onto confluent ST cells; after 48 h, cells were fixed in 4% formaldehyde, stained with 0.1% crystal violet, and scored for cytopathic effect (CPE), with neutralisation defined by absence of CPE. For pH1N1 assays, equal volumes of diluted serum and pH1N1 (pre-titrated to yield plateau infection) were incubated for 2 h, then MDCKSIAT1 cells (3 × 10⁵ cells ml^−1^; 100 µl per well) were added and incubated for 18 h. Post incubation, cells were fixed, permeabilised, and immuno stained for pH1N1 nucleoprotein (Bio-Rad, clone: AA5H) followed by HRP conjugated secondary antibody; TMB substrate was added, the reaction stopped with 1 M H₂SO₄, and absorbance read at 450/630 nm. 50% inhibitory titres (ID₅₀) for both viruses were calculated by Reed–Muench (PRCV) or by linear interpolation between control and infected wells (pH1N1).

### Enzyme-linked immunosorbent spot (ELISpot) assay

MultiScreen™-HA ELISPOT 96 wells plates (Millipore, Watford, UK) were coated with 0.5 µg ml^−1^ mouse anti-pig IFNγ (BD Biosciences, San Jose, CA) or 10 µg ml^−1^ mouse anti-pig IL-2 (Mabtech AB, Nacka Strand, Sweden) in carbonate buffer (0.1 M Na2CO3/NaHCO3, pH 9.6) overnight at 4 °C. Following three washes with PBS, 200 µl blocking buffer was added per well (RPMI1640, 10% FBS, supplemented with 100 U ml^−1^ penicillin and 100 µg ml^−1^ streptomycin), and plates were incubated at 37 °C for 2 h. After blocking buffer removal, 100 µl of thawed BAL and PBMC cell suspension (2 × 10^6^ cells/ml) were added to each well, topped up with 100 µl of stimuli in triplicates and incubated at 37 °C with 5% CO_2_ for 48 h.

Cells obtained from PRCV-infected animals were stimulated for 48 h with either live PRCV (MOI = 1) or peptide pools spanning the PRCV spike (S) or nucleocapsid (N) proteins (16-mer peptides with 12-residue overlap). Similarly, cells obtained from pH1N1-infected animals were stimulated with live pH1N1 (MOI = 1) or peptide pools spanning haemagglutinin (HA) or nucleoprotein (NP) (18-mer peptides with 14-residue overlap). Wells treated with medium alone (RPMI1640 supplemented with GlutaMAX™, 25 mM HEPES, penicillin, streptomycin, and 10% heat-inactivated FBS) served as negative controls, and 2.5 µg ml^−1^ Concanavalin A (Invitrogen) was included as a positive control. Following 48 h of stimulation, plates were washed with wash buffer, containing PBS and 0.05% Tween 20. Thereafter, 100 µl per well of biotinylated mouse anti-pig IFNγ (0.5 µg ml^−1^, BD Biosciences) or mouse anti-pig IL-2 (1 µg ml^−1^, Mabtech) antibodies were added and incubated at room temperature for 2 h. The plates were then washed five times, followed by incubation with a streptavidin alkaline phosphatase conjugate (1:1000, Invitrogen Ltd., 100 µl per well) at room temperature for 1 h. Following five washes, spots were developed using 100 µl per well of alkaline phosphatase substrate solution (Bio-Rad Laboratories, Hercules, CA) at room temperature for 20 min. The plates were washed with tap water and kept at room temperature in the dark overnight to dry. Spots were counted using a CTL Immunospot reader (CTL Europe GmbH, Rutesheim, Germany). The results were expressed as the number of IFNγ or IL-2-producing cells per 10^6^ cells, after subtracting the average number of spots in the medium control wells.

### Intracellular cytokine staining (ICS)

Cryopreserved BAL and PBMC were thawed, and 10^6^ cells per well were plated in duplicates in U-bottom 96-well plates. Cells were stimulated overnight with PRCV and pH1N1 live virus at a MOI of 1, or for 5 h with overlapping peptide pools covering the S/HA or N/NP proteins for PRCV/pH1N1-infected animals and 3 control animals for each group. One well per animal was incubated with media alone (RPMI1640 with GlutaMAX™, 100 IU ml^−1^ penicillin, 100 µg ml^−1^ streptomycin, and 10% FBS) as a negative control, and positive control wells were incubated with a 1/500 dilution of a phorbol 12-myristate 13-acetate (PMA) and ionomycin cocktail (BioLegend). A 1/1000 dilution of Brefeldin A (GolgiPlug™, BD Biosciences) was added to all wells 4 h before harvest. All incubations were carried out at 37 °C with 5% CO_2_.

Following harvest, cells were centrifuged at 500 × *g* for 4 min and washed twice with PBS. Unconjugated anti-swine TCRδ mAbs were added to all samples at 4 °C for 20 min in the dark. Then, cells were washed twice with PBS and incubated with rat anti-mouse IgG1-BUV395 for 20 min at 4 °C in the dark. Following two washes, a blocking step was performed using a 1/20 dilution of mouse sera for 20 min at 4°C in the dark. After blocking, a cocktail of surface antibodies was added (Table 2, **section A**). One hundred microliters of BD Cytofix/Cytoperm (BD Biosciences, Cat. no. 555028) were added to each well for fixation and permeabilization. Cells were then stained with antibodies against IFN-γ and TNF for 30 min at 4 °C.

**Table 2:** List of antibodies used for flow cytometry technique

A) Intracellular cytokine staining panel				
Marker	Clone	Provider	Dilution	Fluorochrome
CD4	74-12-4	BD Biosciences	1/400	PE-Cy7
CD8β	PPT23	Bio-Rad	1/20	PE
CD8α	MIL12	Bio-Rad	1/10	StarBright Violet 515
CD3ε	PPT3	Bio-Rad	1/80	StarBright Violet 570
CD27	b30c7	Bio-Rad	1/10	APC
CCR7	3D12	BD Biosciences	1/40	BV711
CD45RA	MIL13	Bio-Rad	1/100	FITC
IFN-γ	P2G10	BD Biosciences	1/400	PerCP-Cy 5.5
TNF	Mab11	Biolegend	1/100	BV421
TCRδ	PGBL22A	Cambridge Bioscience	1/400	Unconjugated
Anti-IgG1	A85-1	BD Biosciences	1/400	BUV395
**B) B cells and plasma cells staining panel**				
Marker	Clone	Provider	Dilution	Fluorochrome
CD79α	HM47	Biolegend	1/40	PerCP-Cy5.5
CD11c	3A8	Bio-Rad	1/20	FITC
Blimp1	3H2E8	Santa Cruz	1/10	AF647
IRF4	3E4	Fisher Scientific	1/5	PE
PAX5	1H9	BD Biosciences	1/10	BV421
BCL6	K112-91	BD Biosciences	1/20	BUV494
Ki67	B56	BD Biosciences	1/40	BUV395
Streptavidin		BD Biosciences	1/500	BUV615
Tbet	(eBio4B10 (4B10))	Fisher Scientific	1/20	PECy5
**C) T helper, Treg, Tfh staining panel**				
Marker	Clone	Provider	Dilution	Fluorochrome
CD4	74-12-4	BD Biosciences	1/100	PerCP-Cy 5.5
CD3ε	PPT3	Bio-Rad	1/80	StarBright Violet 570
CD8α	76-2-11	BD Biosciences	1/10	AF647
CD25	b30c7	Bio-Rad	1/20	FITC
ICOS	C398.4 A	Biolegend	1/100	BV605
BCL6	K112-91	BD Biosciences	1/20	BUV494
FOXP3	FJK-16s	Fisher Scientific	1/20	PerCP-Cy 5.5
KI67	B56	BD Biosciences	1/40	BUV395
CCR7	3D12	BD Biosciences	1/40	BV711

Data acquisition was performed using a Cytek Aurora full-spectrum flow cytometer (Cytek Biosciences, Fremont, CA, USA), which is equipped with five lasers (355 nm UV, 405 nm violet, 488 nm blue, 561 nm yellow-green, and 640 nm red) and 64 fluorescence detection channels distributed across the lasers (UV: 16 channels, violet: 16, blue: 14, yellow-green: 10, and red: 8). Spectral unmixing was performed by the SpectroFlo software (version 3.2.1, Cytek Biosciences). *t*-SNE followed by FlowSOM analysis was performed on concatenated samples from stimulated cells using FlowJo software version 10.10.0 to explore heatmap of marker expression. Conventional gating strategies were then applied, and results from the stimulated wells were subtracted from those of the medium-treated wells.

### Phenotypic analysis of B and T cells by flow cytometry

Thawed cell suspensions from BAL and PBMC were resuspended in 10 ml of complete RPMI1640 medium and centrifuged at 500 × *g* for 5 min. Subsequently, 2 × 10⁶ cells per sample were transferred into 96-well U-bottom plates and washed twice with PBS. A cocktail of primary antibodies specific for either B cells or T cells (listed in Table 2**, sections B and C)** was added to the wells and incubated for 20 min at 4 °C. Plates were then washed twice with PBS.

For intracellular staining, fixation and permeabilization were performed using the eBioscience Foxp3/Transcription Factor Fixation/Permeabilization concentrate and diluent (Invitrogen) according to manufacturer’s instructions. After two PBS washes, antibodies specific for intracellular markers (Table 2**, sections B and C**) were added and incubated for 30 min at 4 °C.

For the detection of antigen-specific B cells, biotinylated recombinant PRCV spike or pH1N1 HA proteins were added to the appropriate wells and incubated for 40 min at room temperature in the dark. Following two PBS washes, streptavidin conjugates were added to detect the biotinylated mAbs. Finally, cells were resuspended in PBS.

Samples were acquired on the Cytek Aurora flow cytometer introduced above, and data were analyzed using FlowJo software. For T helper cell phenotyping analysis, CD3⁺CD4⁺Foxp3⁻Bcl6⁻ cells were concatenated across all samples. t-SNE analysis wa performed followed by combinatorial gating using CCR7, CD8α, CD25, ICOS, and Ki-67 markers.

### Nasal microbiome profiling

Bacterial DNA was extracted from nasal swabs using the DNeasy PowerSoil kit (Qiagen, UK) following the manufacturer’s protocol. Forty-three samples from 10 pigs collected over the infection time course were analysed. The V4–V5 regions of the 16S rRNA gene were amplified with primers U515F (5′-GTGYCAGCMGCCGCGGTA-3′) and U927R (5′-CCCGYCAATTCMTTTRAGT-3′) containing barcodes. PCR reactions (15 μL) included 0.2 μM primers, ~10 ng DNA, and Phusion High-Fidelity Master Mix. Cycling conditions were 98 °C for 1 min; 30 cycles of 98 °C for 10 s, 50 °C for 30 s, 72 °C for 30 s; and 72 °C for 5 min. Products were purified with magnetic beads, quantified (Qubit, qPCR), and assessed for size (Bioanalyzer). Libraries were pooled and sequenced on an Illumina MiSeq (2 × 300 bp). Reads were merged with FLASH v1.2.11 and processed in QIIME2 (v2018.4). DADA2 was used for quality filtering and ASV generation. Multiple sequence alignment enabled phylogenetic analysis, and ASV counts were rarefied to the smallest library size for alpha and beta diversity calculations. Taxonomy was assigned using a SILVA-trained classifier. The top 10 families per sample were visualised as relative abundance histograms. Differences at the family level were tested using t-tests between control, PRCV-, and pH1N1-infected pigs. Longitudinal alpha diversity (Shannon index) was assessed with the qiime2-longitudinal plugin.

### Statistical analysis

Statistical analyses were performed using GraphPad Prism 9.2.0 (GraphPad Software, San Diego, CA, USA). The specific test applied is indicated in the corresponding figure legends.

### Single cell RNA-Seq library preparation and sequencing

Eighteen samples were prepared for single cell transcriptomic analysis using the 10×3’ Gene expression kit. Samples were adjusted to 10,000 cells and then partitioned using the Chromium iX Controller, the Next GEM Chip G, and the Single Cell 3′ Kit v3.1 library preparation kit (10x Genomics, Pleasanton, CA, USA). Sequencing library quality was determined using the Qubit DNA BR kit (Life Technologies, UK), Illumina library quantitation kit (NEB, US) and the Tapestation D5000 DNA BR kit (Agilent, US). Sequencing libraries were pooled randomly across 2 NextSeq 2000 P4 100 cycle kit (Illumina, San Diego, California, United States), each with 1% PhiX spikes, aiming for approximately 200 million reads per sample. Runs formats aligned with manufacturers recommendations.

### Single-cell RNA-Seq data pre-processing and quality control

Twenty samples were examined to explore their scRNA-seq profiles. Their BCL files were demultiplexed using the mkfastq function in Cell Ranger v7.1.0. The reads were mapped to the *Sus scrofa* reference genome (assembly 11.1, GCA_000003025.6) using STAR v2.7.2a in Cell Ranger. Unique molecular identifier (UMI) counting was implemented with Cell Ranger count. Subsequent data processing and analyses were performed in R v4.3.2.

To enhance data quality and mitigate technical noise, we applied stringent cell filtering criteria to identify and remove empty droplets, multiplets, low quality cells and dead or damaged cells. Firstly, empty droplets and barcode-swapped droplets were identified and removed using DropletUtils v1.22.0^76,77^. Secondly, cells with total UMI counts >50,000 or <1000 were excluded. Thirdly, cells with low gene detection (<500 genes), elevated mitochondrial gene expression (>20%), or predicted to be doublets by scds v1.18.0 were removed. Fourthly, genes with zero expression across all cells were omitted.

To account for cell library size differences, scaling normalisation was applied using computePooledFactors from the scuttle package (v1.12.0), followed by log2 transformation with a pseudo-count of 1^78^. The 20 samples came from three independent runs, which were integrated using the fastMNN algorithm implemented in the batchelor package v1.18.1 to correct for batch effects^79^.

### Clustering and annotation of scRNA-seq libraries

Shared nearest-neighbour (SNN) graph-based clustering was performed using clusterCells (bluster v1.12.0). The top 5000 highly variable genes (HVGs) were identified by getTopHVGs in scran (v1.30.2)^80^. Clustering was conducted using the Jaccard index to weight edges and the Louvain method for community detection, with the number of neighbours set to *k* = 25. This initial clustering yielded 20 distinct clusters. To assign biological meaning, clusters were annotated using canonical cell-type markers and grouped into three major subsets: myeloid cells, T/B/NK-like T (lymphoid) cells, and non-immune structural cells (Supplementary Data 1A). The immune cell proportions were examined using PERMANOVA (Permutational Multivariate Analysis of Variance) using vegan v2.7-2, where the data were scaled as centred log-ratios (CLRs) using compositions v2.0-9. This was visualised with dplyr v1.1.4, tidyr v1.3.1, ggplot2 v3.4.3 and rstatix v0.7.3.

Within each subset, cells were re-integrated and re-clustered using the same framework. For the myeloid subset, 20 refined clusters were identified and annotated. For the T/B/NK-like T cell subset, the first round of analysis produced 13 clusters. Clusters 11 (annotated as “unknown”) and 12 (expressing mast cell–associated markers which did not belong to T/B/NK-like T cell types) were removed from the UMAP visualization. We extracted seven clusters that were enriched for T cell markers, re-integrated them, and performed a second round of clustering to improve the resolution of T cell subtypes and remove noise. This resulted in 5 new clusters (labelled *1re–5re* to distinguish them from the initial clustering). In the non-immune subset, 22 clusters were identified. Cluster 22 (expressing immune cell–related markers) was excluded from the final UMAP representation because it did not belong to non-immune structural cell types.

Clusters across all three subsets were manually annotated with conventional cell-type names based on marker gene expression (Supplementary Data 1B, C; **heatmap in** Supplementary Figs. 10–12). The top differentially expressed genes for each cluster were identified using the scoreMarkers function (scran v1.30.2) to characterize transcriptional signatures (Supplementary Data 2).

### Cell type scRNA-seq differential abundance and expression analyses

We assessed changes in the differential abundance (DA) of subpopulations in the three cell subsets across experimental conditions (control, PRCV infection, H1N1 infection) at both time points (1 and 12 DPI). To achieve DA testing, we used edgeR (v4.6.3) in R (v4.5.0) twith a negative binomial generalized linear model (GLM) with empirical Bayes quasi-likelihood *F*-tests. For each time point, the model incorporated the three experimental conditions (control, PRCV, H1N1) and tested for pairwise differences between conditions, including the sequencing run as a covariate for potential batch effects. Differences in read depth were adjusted by using an offset of the counts of cells per cluster per sample, scaled by the total number of cells per sample. Trend estimation was disabled during both dispersion estimation and quasi-likelihood dispersion fitting to avoid over-fitting with few replicates in certain clusters. Multiple test correction was implemented using a Benjamini-Hochberg approach.

The pairwise comparisons examined for DE per cell type cluster were: at 1DPI: pH1N1(*n* = 3) vs control (*n* = 4); pH1N1 (*n* = 3) vs PRCV (*n* = 4), and at 12 DPI: pH1N1 (*n* = 5) vs control (*n* = 4); PRCV (*n* = 4) vs control (*n* = 4) and PRCV (*n* = 4) vs pH1N1 (*n* = 5). We employed a pseudo-bulk strategy to test for DE within each cluster: counts from all cells in the same cluster in each sample were aggregated, generating pseudo-bulk profiles for each cluster–sample pair. Cluster-sample pairs with < 10 cells were excluded. As above, a negative binomial GLM was fitted separately for each cluster, and DE was assessed across the group pairs (Supplementary Data 3) using empirical Bayes quasi-likelihood *F*-tests implemented in edgeR (v4.6.3) with the sequencing run as a covariate. Multiple test correction was implemented using a Benjamini-Hochberg approach. The full results of the DE analyses are reported in Supplementary Data 4.

### Gene function and pathway enrichment scRNA-seq analysis

The genes’ adjusted *p*-values, and log2FC values from the edgeR analyses were used for functional and pathway enrichment analyses using clusterProfiler (v4.15.1). This used reference annotation from the KEGG to apply GSEA^81,82^. GSEA used a permutation-based strategy to evaluate ranked gene lists, sorted by the logFC, thereby detecting gene sets in which many genes exhibit coordinated changes. The resulting *p*-values were Benjamini–Hochberg corrected. KEGG pathways with *q* < 0.05 were visualised in bubble plots. The full results of the GSEA are provided in Supplementary Data 5.

### Reporting summary

Further information on research design is available in the Nature Portfolio Reporting Summary linked to this article.

## Supplementary information

Clean Supplementary Figs.

Description of Additional Supplementary Files

Supplementary Data 1 - 6

Reporting summary

## Data Availability

The raw and processed scRNA-sequencing data (Fastq and h5 files) have been deposited to the GEO under accession number (GSE314861) https://www.ncbi.nlm.nih.gov/geo/query/acc.cgi?acc=GSE314861. The sequences of the PRCV Spike containing plasmid are submitted to GenBank NCBI: PZ305746. 16S rRNA amplicon sequencing reads (FASTQ and FASTA files) generated in this study have been deposited in a public dataset in Zenodo (https://zenodo.org/records/18851034) under 10.5281/zenodo.18851034^83^ and European Nucleotide Archive (ENA) under the accession number PRJEB113232. Numerical data for the graphs and charts in the figures are provided in Supplementary Data 6. All other data are available from the corresponding author on reasonable request.

## Code availability

All R scripts used for processing and analysis of the scRNA-seq data are available at: https://github.com/LynnGoodnight/bsp248, with a mirrored deposition to Zenodo under 10.5281/zenodo.19497339.
