# Supplementary material for: Pathogen-specific immune responses might underlie divergent outcomes of coronavirus and influenza infection in the natural porcine host
Source: Commun Biol. 2026 May 30;9:1043. doi: 10.1038/s42003-026-10400-y (PMC13444145; doi:10.1038/s42003-026-10400-y)
Supplement: Supplementary file 1 [file 42003_2026_10400_MOESM1_ESM.pdf]

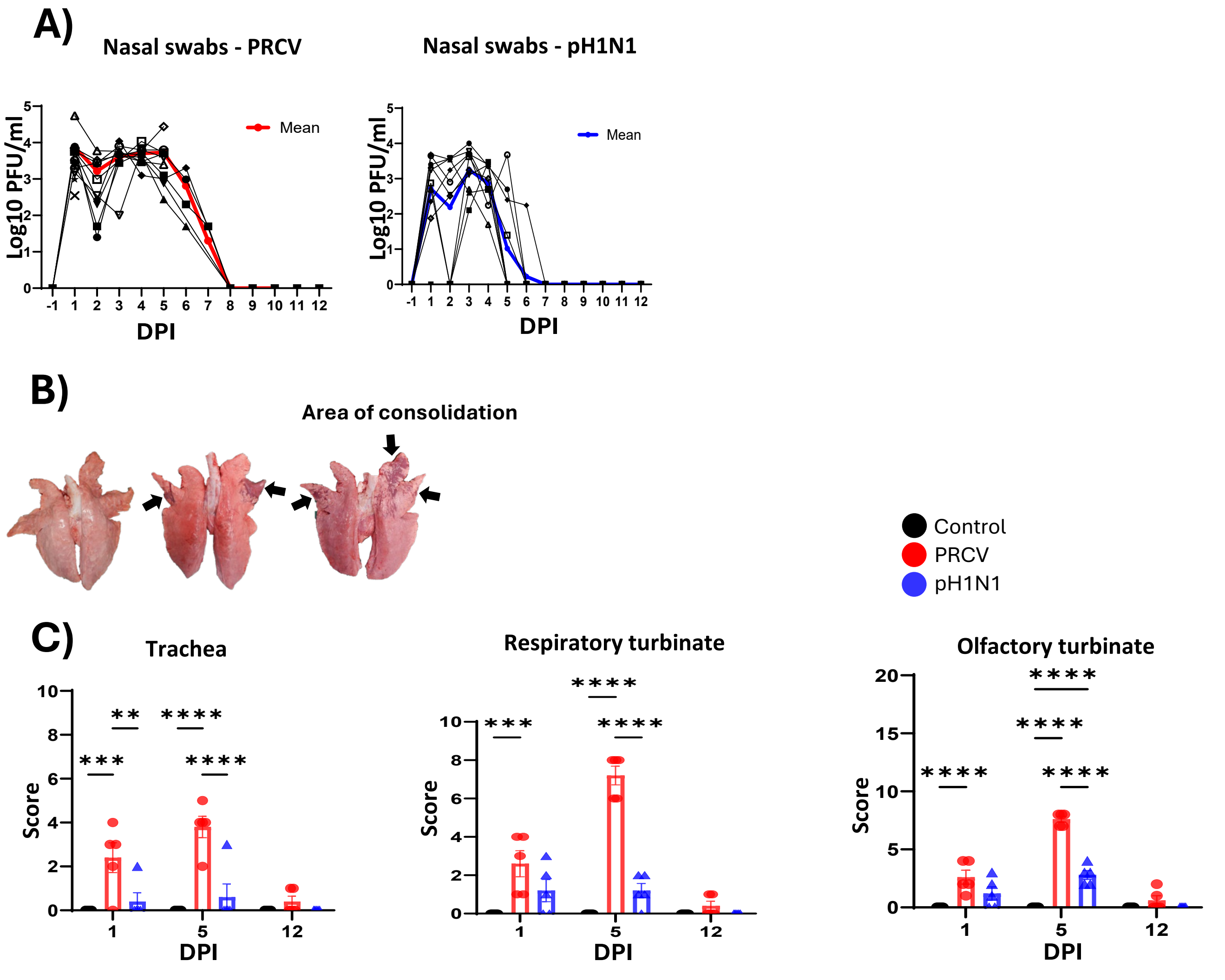

Supplementary Figure 1. Nasal swab viral load, lung pathology, and multiplex immunohistochemistry following PRCV or pH1N1 infection. A) Viral load in daily nasal swabs were quantified using plaque assays for PRCV (red) and pH1N1 (blue). **B)** Representative gross lung pathology images from control, PRCV-, and pH1N1-infected pigs at 5 DPI. **C)** Histopathology and immunohistochemistry scores of the trachea, respiratory turbinate, and olfactory turbinate in control (black), PRCV (red), and pH1N1 (blue) groups at 1, 5, and 12 DPI. Each point represents an individual animal; bars indicate the mean ± SEM. Statistical analysis was performed using two-way ANOVA with Bonferroni's multiple comparisons test. Asterisks indicate significance: *p < 0.05, **p < 0.01, ***p < 0.001, ****p < 0.0001.

A)

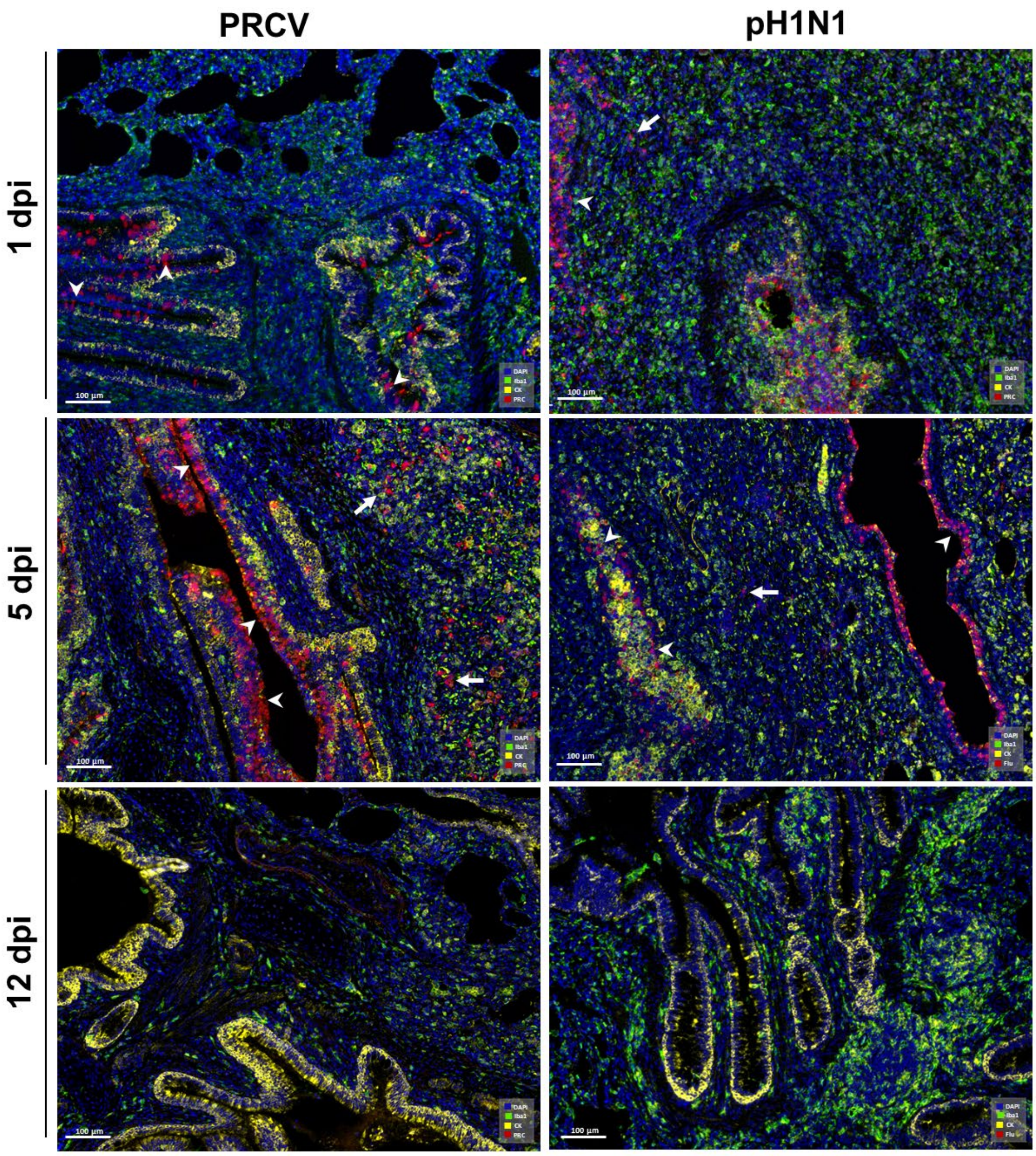
PRCV
pH1N1
1 dpi
5 dpi
12 dpi
DAPI
Iba1
CK
PRC
Flu
100 µm

B)

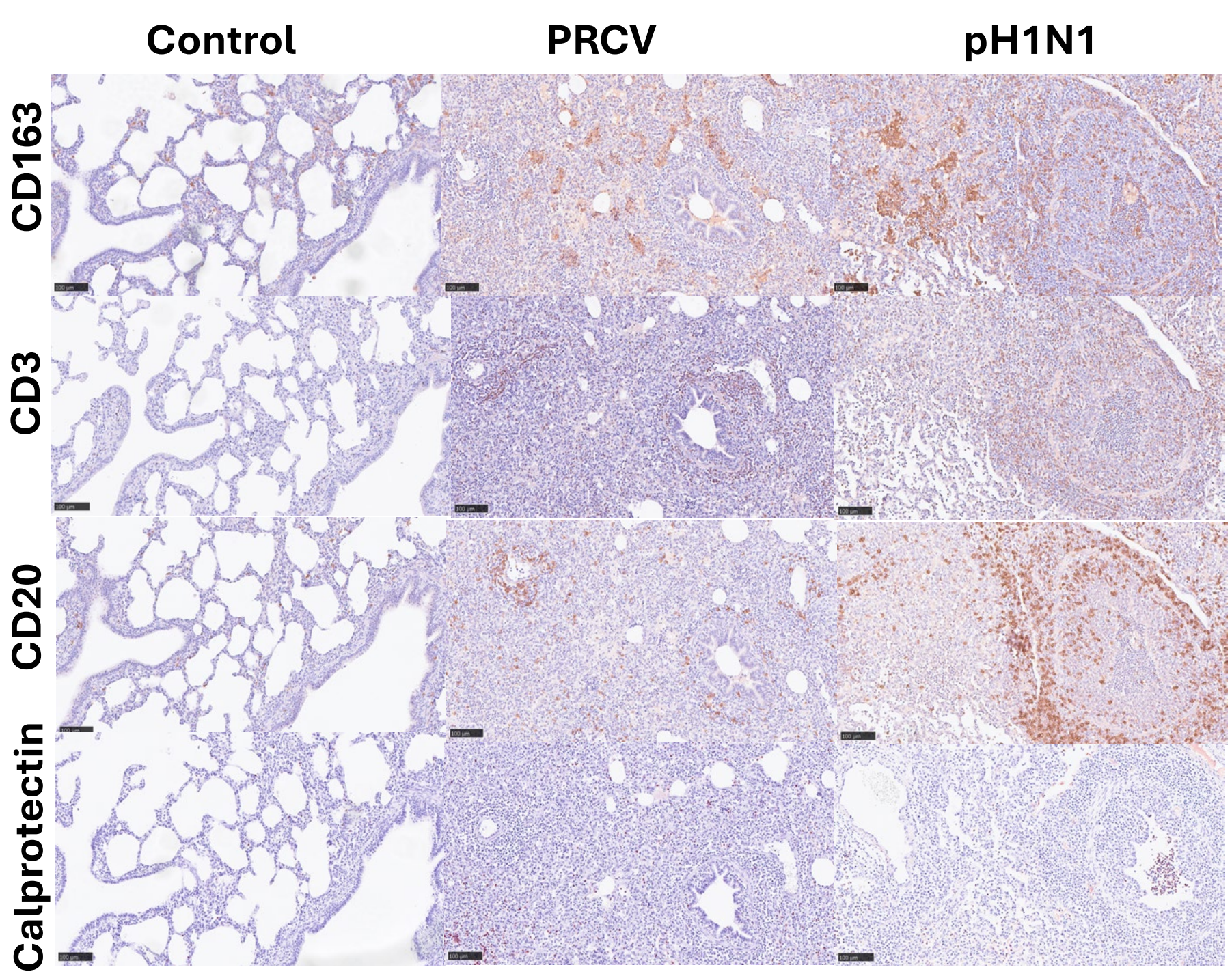
Control
PRCV
pH1N1
CD163
CD3
CD20
Calprotectin

Supplementary Figure 2. Multiplex immunohistochemistry (mIHC) of lungs from PRCV- and pH1N1-infected animals at 1, 5, and 12 days post-infection (DPI). A) Representative mIHC images show pH1N1 NP^+ or PRCV N^+ cells (red), $Iba1^+$ cells (green), pan-cytokeratin ($pan\text{-}CK^+$, yellow), and nuclear staining with DAPI (blue) in lung tissue sections following PRCV or pH1N1 infection. At 1 DPI, PRCV-infected lung shows N+ cells (red, arrowheads) located within the respiratory epithelium (yellow, CK+). Abundant Iba1+ cells (green) are also evident. At 1 DPI, pH1N1 infected animal shows high number of iba1+ cells (green) in an area of interstitial pneumonia. NP+ cells (red) are located within the intraluminal exudate within a bronchiole (arrowheads). There are few Pan-CK + cells (yellow) and several cells are sloughed off from the epithelium and present within the inflammatory infiltrate (arrow). At 5 DPI, PRCV infected lung show high numbers of N+ cells (red, arrowheads) located within the respiratory epithelium (yellow, PanCK+), and few scattered cells (arrows) in areas of interstitial pneumonia associated with abundant Iba1+ cells (green). pH1N1 infected lungs at 5 DPI shows abundant Iba1+ cells (green) in areas of interstitial pneumonia associated with few NP+ cells (red) (arrowheads). Sloughed Pan-CK+ cells from the respiratory epithelium can be observed within the inflammatory infiltrates (arrow). At 12 DPI, PRCV and pH1N1 infected lungs shows high numbers of pan-CK+ cells (yellow) within a normal/healed airway epithelium. Scale bar = 100µm. **B)** Representative images of immunohistochemistry performed on lung tissue sections to quantify $CD163^+$ (macrophages), $CD3^+$ (T cells), $CD20^+$ (B cells), and $Calprotectin^+$ (neutrophils) within the lesions.

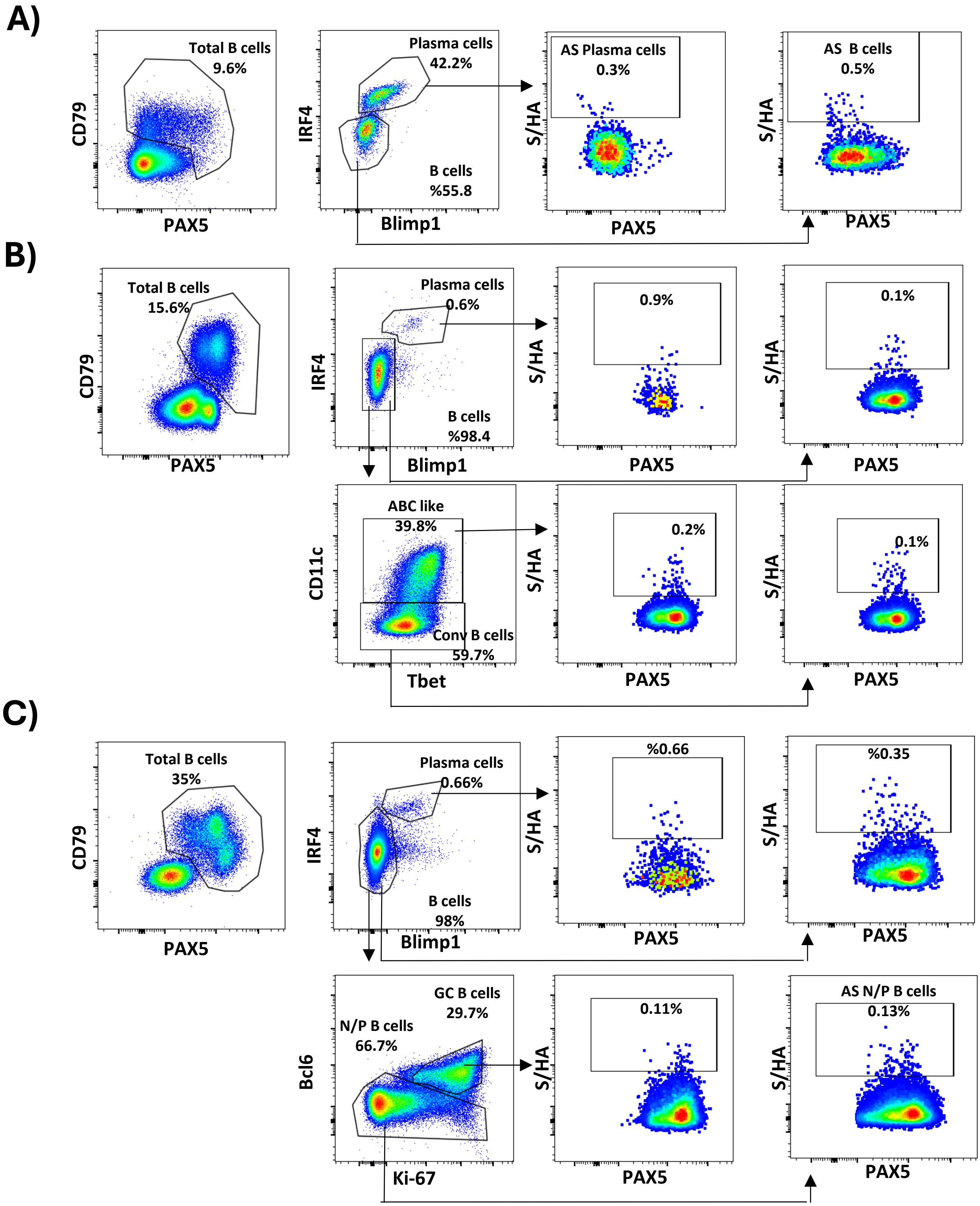

Supplementary Figure 3. Flow cytometry gating strategy for identification of PRCV spike- and pH1N1 HA-specific B cells. Following necropsy at 1, 5, and 12 days post-infection (DPI), single-cell suspensions were prepared from **A)** BAL, **B)** PBMC, and **C)** TBLN. Cells were stained to identify PRCV spike- or pH1N1 HA-specific B cells (Blimp-1^{-} IRF4^{+}/$^{-}$), and plasma cells (Blimp-1^{+} IRF4^{+}). In PBMC, antigen-specific atypical B cells (CD11c^{+} T-bet^{+}/$^{-}$) and conventional B cells (CD11c^{-} T-bet^{+}/$^{-}$) were also identified. In TBLN, spike/HA-specific GC B cells (Bcl-6^{+} Ki-67^{+}) and naïve/proliferating B cells (Bcl-6^{-} Ki-67^{+}/$^{-}$) were detected.

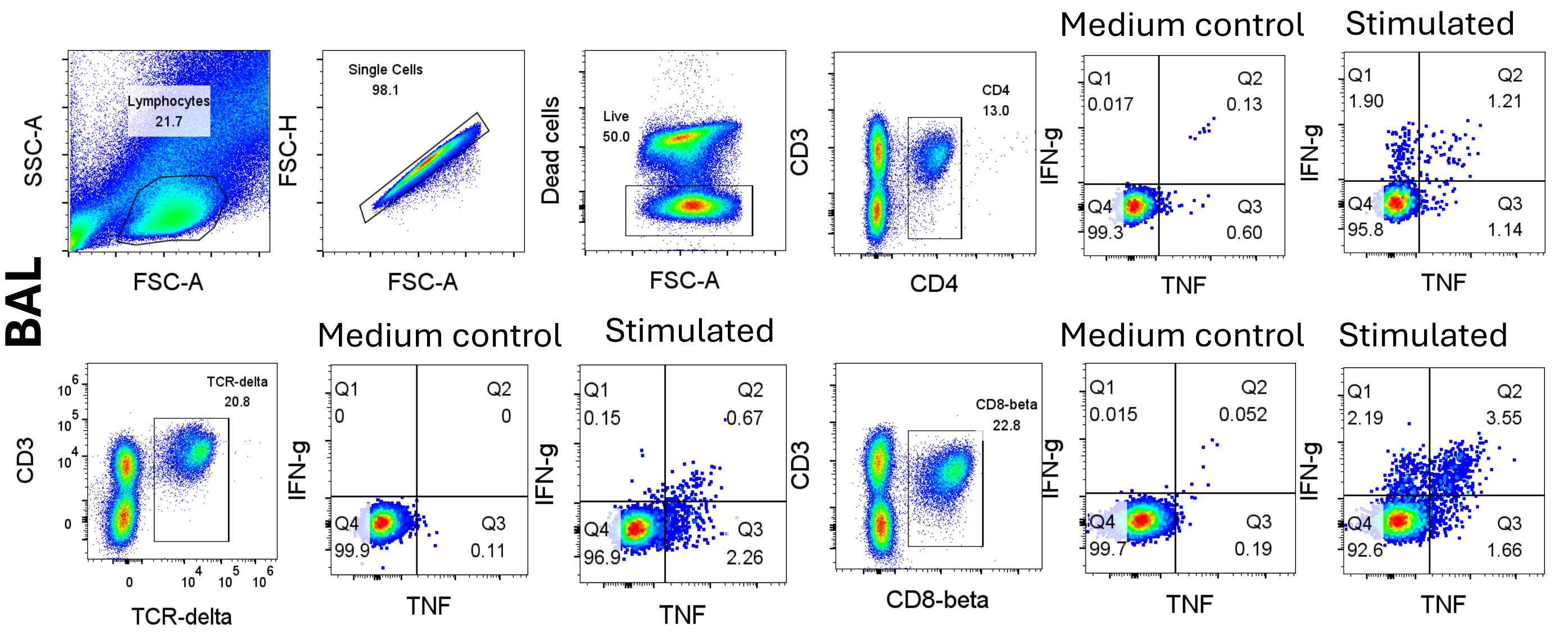

Supplementary Figure 4. Gating strategy for intracellular cytokine staining of CD4⁺, CD8⁺, and γδ⁺ T cells in BAL. Cryopreserved BAL samples from control, PRCV-, and pH1N1-infected animals were thawed and stained for intracellular cytokine detection by flow cytometry. The sequential gating strategy (from left to right) was used to identify IFNγ⁺, TNF⁺, and IFNγ⁺TNF⁺ double-producing CD4⁺, CD8⁺, and γδ⁺ T cells in BAL and PBMC samples.

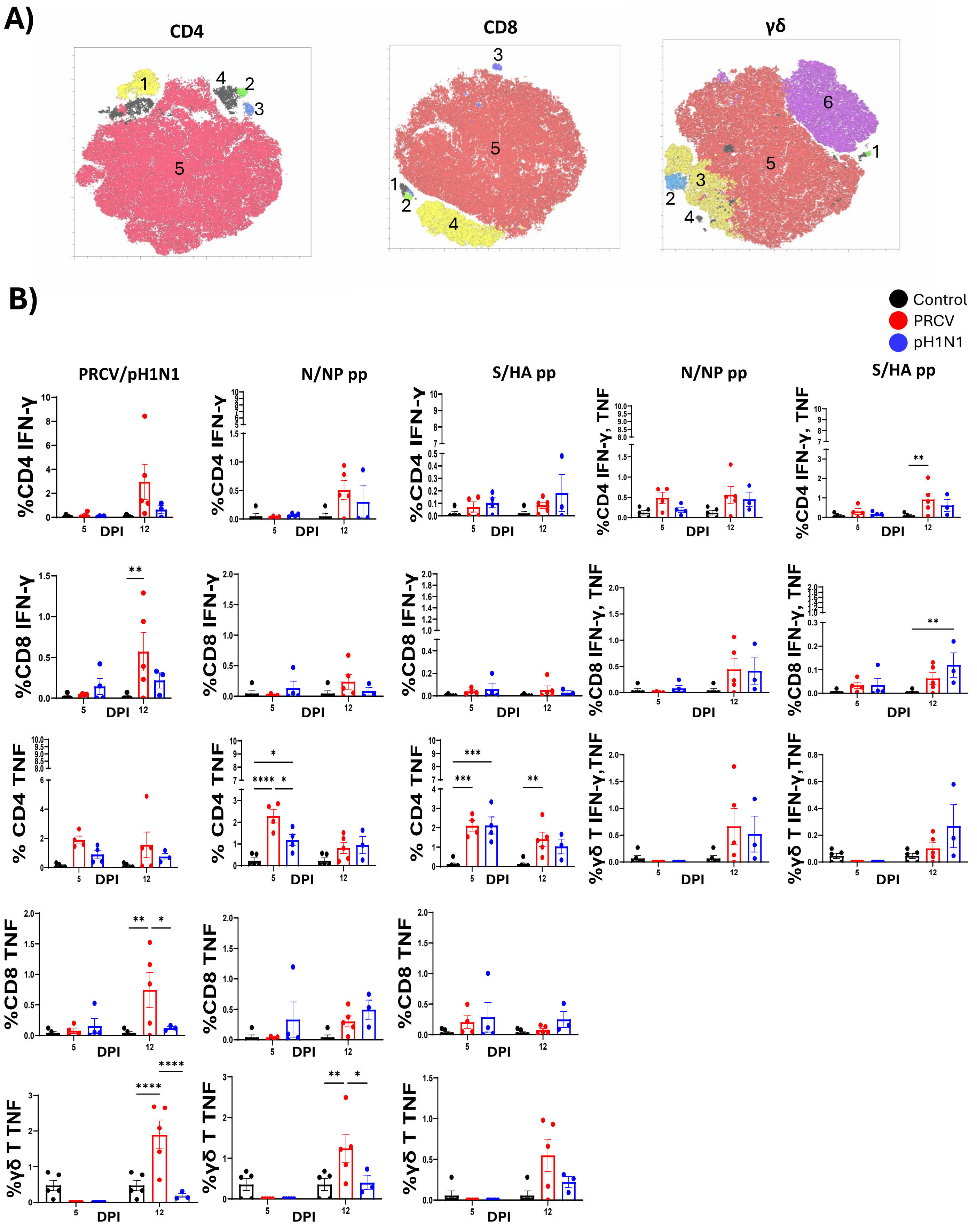

A)
CD4
CD8
γδ
B)
Control
PRCV
pH1N1
PRCV/pH1N1
N/NP pp
S/HA pp
N/NP pp
S/HA pp
%CD4 IFN-γ
%CD8 IFN-γ
% CD4 TNF
%CD8 TNF
%γδ T TNF
%CD4 IFN-γ, TNF
%CD8 IFN-γ, TNF
%γδ T IFN-γ,TNF
DPI

Supplementary Figure 5. Phenotype of cytokine-secreting T cell responses in BAL following PRCV or pH1N1 infection. Cryopreserved BAL cells were stimulated *ex vivo* with either live virus (PRCV or pH1N1), PRCV nucleocapsid (N)/pH1N1 nucleoprotein (NP), or PRCV spike (S)/pH1N1 hemagglutinin (HA) peptide pools. Following stimulation, cells were stained with antibodies against CD3, CD4, CD8β, CD8α, CCR7, CD27, CD45RA, IFN-γ, and TNF, and analyzed by flow cytometry to assess the phenotype of cytokine-secreting $CD4^+$, $CD8^+$, and $\gamma\delta^+$ T cells.**A)** t-SNE analysis followed by FlowSOM clustering was performed on concatenated *ex vivo*–stimulated $CD4^+$, $CD8^+$, and $\gamma\delta^+$ T cells, with color-coded clusters indicating distinct populations. **B)** Intracellular cytokine staining (ICS) analysis showing frequencies of IFN-γ^+, TNF^+, and IFN-γ^+TNF^+ double-producing $CD4^+$, $CD8^+$, and $\gamma\delta^+$ T cells in BAL from control (black), PRCV-infected (red), and pH1N1-infected (blue) animals. Each point represents an individual animal; bars indicate the mean ± SEM. Statistical analysis was performed using two-way ANOVA with Bonferroni's multiple comparisons test. Asterisks indicate significance: *$p < 0.05$, **$p < 0.01$, ***$p < 0.001$, ****$p < 0.0001$.

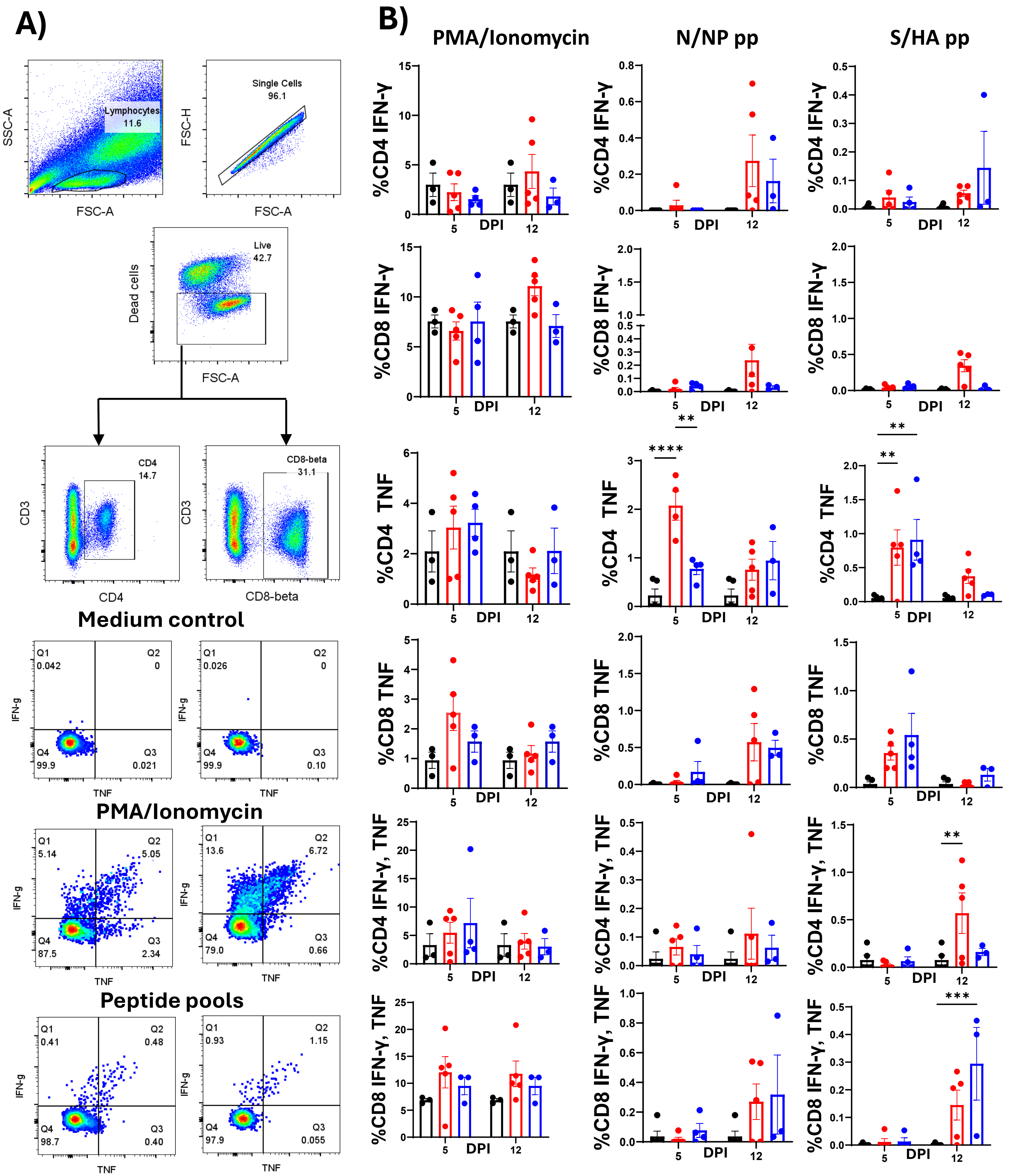

Supplementary Figure 6. CD4⁺ and CD8⁺ T cell responses in BAL following PRCV or pH1N1 infection. Cryopreserved BAL cells were stimulated *ex vivo* with PMA/Ionomycin, PRCV nucleocapsid (N)/pH1N1 nucleoprotein (NP), or PRCV spike (S)/pH1N1 hemagglutinin (HA) peptide pools. Following stimulation, cells were stained with antibodies against CD3, CD4, CD8β, IFN-γ, and TNF, and analyzed by flow cytometry to assess the proportion of cytokine-secreting CD4⁺ and CD8⁺T cells. **A)** The sequential gating strategy (from left to right) was used to identify IFNγ⁺, TNF⁺, and IFNγ⁺TNF⁺ CD4⁺ and CD8⁺T cells in BAL. **B)** Intracellular cytokine staining (ICS) analysis showing frequencies of IFN-γ⁺, TNF⁺, and IFN-γ⁺TNF⁺ CD4⁺ and CD8⁺ T cells in BAL from control (black), PRCV-infected (red), and pH1N1-infected (blue) pigs. Each point represents an individual animal; bars indicate the mean ± SEM. Statistical analysis was performed using two-way ANOVA with Bonferroni's multiple comparisons test. Asterisks indicate significance: *p < 0.05, **p < 0.01, ***p < 0.001.

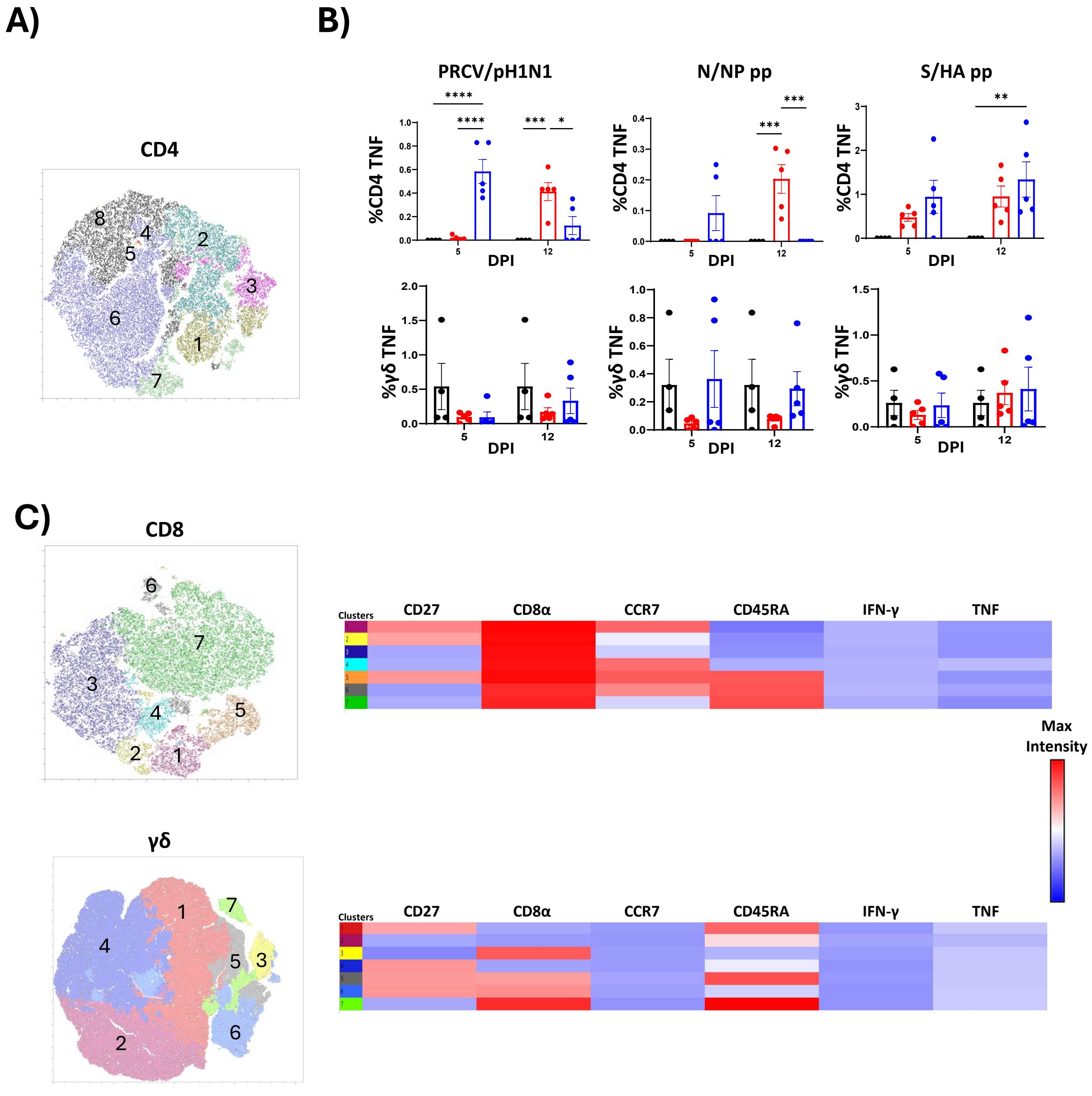

Supplementary Figure 7. Phenotype of cytokine-secreting T cells in PBMC following PRCV or pH1N1 infection. Cryopreserved PBMC cells were *ex vivo* stimulated with either live virus (PRCV or pH1N1), PRCV nucleocapsid (N)/pH1N1 nucleoprotein (NP), or PRCV spike (S)/pH1N1 hemagglutinin (HA) peptide pools. Following stimulation, cells were stained with antibodies against CD3, CD4, CD8β, CD8α, TCRδ, CCR7, CD27, CD45RA, IFN-γ, and TNF, and analyzed by flow cytometry to assess the phenotype of cytokine-secreting $CD4^+$, $CD8^+$, and $\gamma\delta^+$ T cells. **A)** t-SNE analysis followed by FlowSOM clustering was performed on concatenated *ex vivo*–stimulated $CD4^+$, $CD8^+$, and $\gamma\delta^+$ T cells, with color-coded clusters indicating distinct populations heatmap represents intensity of expression of the markers. **B)** Intracellular cytokine staining (ICS) analysis showing frequencies of IFN-γ^+, TNF^+, and IFN-γ^+TNF^+ double-producing $CD4^+$, $CD8^+$, and $\gamma\delta^+$ T cells in PBMC from control (black), PRCV-infected (red), and pH1N1-infected (blue) animals. **C)** t-SNE analysis followed by FlowSOM clustering was performed on concatenated ex vivo–stimulated $CD8^+$, and $\gamma\delta^+$ T cells, with color-coded clusters indicating distinct populations heatmap represents intensity of expression of the markers. Each point on bar chart represents an individual animal; bars indicate the mean ± SEM. Statistical analysis was performed using two-way ANOVA with Bonferroni's multiple comparisons test. Asterisks indicate significance: *$p < 0.05$, **$p < 0.01$, ***$p < 0.001$, ****$p < 0.0001$.

A)

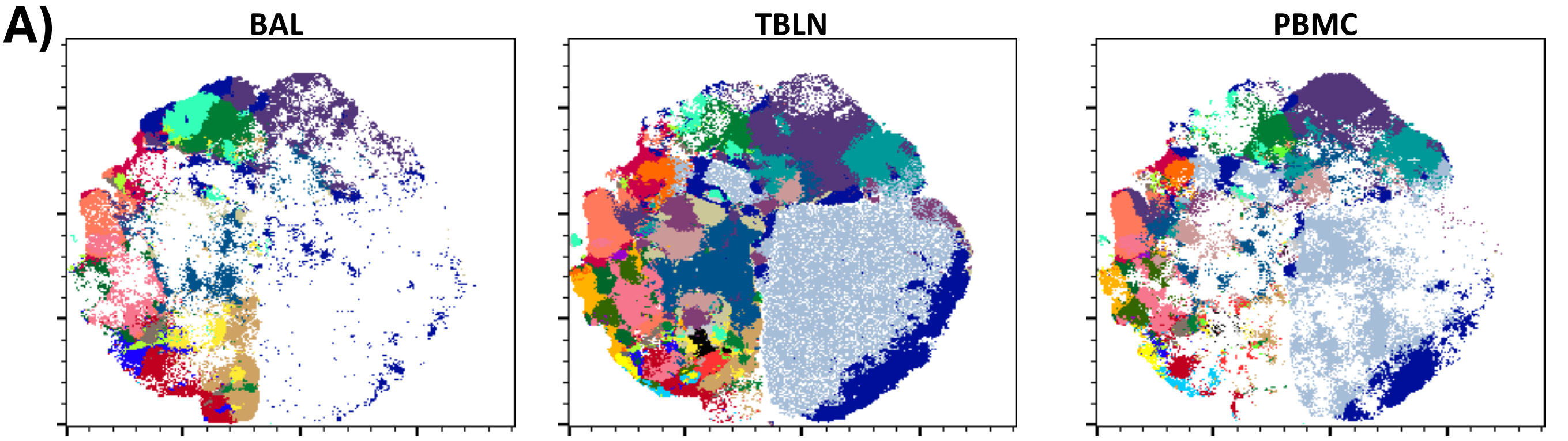

B)

Group	Subset Name (BAL)	Freq. of Parent (BAL)	Subset Name (TBLN)	Freq. of Parent (TBLN)	Subset Name (PBMC)	Freq. of Parent (PBMC)
TEMRA	CCR7-CD8Alpha-CD25-ICOS-Ki67-	4.69	CCR7-CD8Alpha-CD25-ICOS-Ki67-	4.41	CCR7-CD8Alpha-CD25-ICOS-Ki67-	4.47
	CCR7-CD8Alpha-CD25-ICOS-Ki67+	1.82	CCR7-CD8Alpha-CD25-ICOS-Ki67+	0.33	CCR7-CD8Alpha-CD25-ICOS-Ki67+	1.06
	CCR7-CD8Alpha-CD25-ICOS+Ki67-	0.28	CCR7-CD8Alpha-CD25-ICOS+Ki67-	1.47	CCR7-CD8Alpha-CD25-ICOS+Ki67-	0.035
	CCR7-CD8Alpha-CD25-ICOS+Ki67+	0.50	CCR7-CD8Alpha-CD25-ICOS+Ki67+	0.56	CCR7-CD8Alpha-CD25-ICOS+Ki67+	0.20
	CCR7-CD8Alpha-CD25+ICOS-Ki67-	11.0	CCR7-CD8Alpha-CD25+ICOS-Ki67-	0.25	CCR7-CD8Alpha-CD25+ICOS-Ki67-	0.27
	CCR7-CD8Alpha-CD25+ICOS-Ki67+	3.65	CCR7-CD8Alpha-CD25+ICOS-Ki67+	0.027	CCR7-CD8Alpha-CD25+ICOS-Ki67+	0.069
	CCR7-CD8Alpha-CD25+ICOS+Ki67-	2.94	CCR7-CD8Alpha-CD25+ICOS+Ki67-	0.25	CCR7-CD8Alpha-CD25+ICOS+Ki67-	0.015
	CCR7-CD8Alpha-CD25+ICOS+Ki67+	3.06	CCR7-CD8Alpha-CD25+ICOS+Ki67+	0.13	CCR7-CD8Alpha-CD25+ICOS+Ki67+	0.070
TEM	CCR7-CD8Alpha+CD25-ICOS-Ki67-	7.63	CCR7-CD8Alpha+CD25-ICOS-Ki67-	5.85	CCR7-CD8Alpha+CD25-ICOS-Ki67-	42.2
	CCR7-CD8Alpha+CD25-ICOS-Ki67+	2.52	CCR7-CD8Alpha+CD25-ICOS-Ki67+	0.81	CCR7-CD8Alpha+CD25-ICOS-Ki67+	7.70
	CCR7-CD8Alpha+CD25-ICOS+Ki67-	1.36	CCR7-CD8Alpha+CD25-ICOS+Ki67-	9.76	CCR7-CD8Alpha+CD25-ICOS+Ki67-	0.98
	CCR7-CD8Alpha+CD25-ICOS+Ki67+	1.83	CCR7-CD8Alpha+CD25-ICOS+Ki67+	2.93	CCR7-CD8Alpha+CD25-ICOS+Ki67+	2.37
	CCR7-CD8Alpha+CD25+ICOS-Ki67-	20.1	CCR7-CD8Alpha+CD25+ICOS-Ki67-	0.78	CCR7-CD8Alpha+CD25+ICOS-Ki67-	3.73
	CCR7-CD8Alpha+CD25+ICOS-Ki67+	5.45	CCR7-CD8Alpha+CD25+ICOS-Ki67+	0.13	CCR7-CD8Alpha+CD25+ICOS-Ki67+	0.98
	CCR7-CD8Alpha+CD25+ICOS+Ki67-	17.2	CCR7-CD8Alpha+CD25+ICOS+Ki67-	2.18	CCR7-CD8Alpha+CD25+ICOS+Ki67-	0.46
	CCR7-CD8Alpha+CD25+ICOS+Ki67+	15.9	CCR7-CD8Alpha+CD25+ICOS+Ki67+	1.24	CCR7-CD8Alpha+CD25+ICOS+Ki67+	1.31
Naïve	CCR7+CD8Alpha-CD25-ICOS-Ki67-	0	CCR7+CD8Alpha-CD25-ICOS-Ki67-	44.6	CCR7+CD8Alpha-CD25-ICOS-Ki67-	16.3
	CCR7+CD8Alpha-CD25-ICOS-Ki67+	2.31E-4	CCR7+CD8Alpha-CD25-ICOS-Ki67+	1.47	CCR7+CD8Alpha-CD25-ICOS-Ki67+	0.64
	CCR7+CD8Alpha-CD25-ICOS+Ki67-	2.31E-4	CCR7+CD8Alpha-CD25-ICOS+Ki67-	3.48	CCR7+CD8Alpha-CD25-ICOS+Ki67-	0.15
	CCR7+CD8Alpha-CD25-ICOS+Ki67+	2.31E-4	CCR7+CD8Alpha-CD25-ICOS+Ki67+	1.42	CCR7+CD8Alpha-CD25-ICOS+Ki67+	0.43
	CCR7+CD8Alpha-CD25+ICOS-Ki67-	6.93E-4	CCR7+CD8Alpha-CD25+ICOS-Ki67-	0.35	CCR7+CD8Alpha-CD25+ICOS-Ki67-	0.27
	CCR7+CD8Alpha-CD25+ICOS-Ki67+	2.31E-4	CCR7+CD8Alpha-CD25+ICOS-Ki67+	0.037	CCR7+CD8Alpha-CD25+ICOS-Ki67+	0.033
	CCR7+CD8Alpha-CD25+ICOS+Ki67-	2.31E-4	CCR7+CD8Alpha-CD25+ICOS+Ki67-	0.82	CCR7+CD8Alpha-CD25+ICOS+Ki67-	0.025
	CCR7+CD8Alpha-CD25+ICOS+Ki67+	4.62E-4	CCR7+CD8Alpha-CD25+ICOS+Ki67+	0.42	CCR7+CD8Alpha-CD25+ICOS+Ki67+	0.14
TCM	CCR7+CD8Alpha+CD25-ICOS-Ki67-	0	CCR7+CD8Alpha+CD25-ICOS-Ki67-	4.64	CCR7+CD8Alpha+CD25-ICOS-Ki67-	8.37
	CCR7+CD8Alpha+CD25-ICOS-Ki67+	0	CCR7+CD8Alpha+CD25-ICOS-Ki67+	0.22	CCR7+CD8Alpha+CD25-ICOS-Ki67+	0.53
	CCR7+CD8Alpha+CD25-ICOS+Ki67-	0	CCR7+CD8Alpha+CD25-ICOS+Ki67-	6.22	CCR7+CD8Alpha+CD25-ICOS+Ki67-	2.23
	CCR7+CD8Alpha+CD25-ICOS+Ki67+	0	CCR7+CD8Alpha+CD25-ICOS+Ki67+	2.42	CCR7+CD8Alpha+CD25-ICOS+Ki67+	2.31
	CCR7+CD8Alpha+CD25+ICOS-Ki67-	9.24E-4	CCR7+CD8Alpha+CD25+ICOS-Ki67-	0.25	CCR7+CD8Alpha+CD25+ICOS-Ki67-	1.26
	CCR7+CD8Alpha+CD25+ICOS-Ki67+	2.31E-4	CCR7+CD8Alpha+CD25+ICOS-Ki67+	0.024	CCR7+CD8Alpha+CD25+ICOS-Ki67+	0.12
	CCR7+CD8Alpha+CD25+ICOS+Ki67-	6.93E-4	CCR7+CD8Alpha+CD25+ICOS+Ki67-	1.59	CCR7+CD8Alpha+CD25+ICOS+Ki67-	0.36
	CCR7+CD8Alpha+CD25+ICOS+Ki67+	2.31E-3	CCR7+CD8Alpha+CD25+ICOS+Ki67+	0.94	CCR7+CD8Alpha+CD25+ICOS+Ki67+	0.95

C)

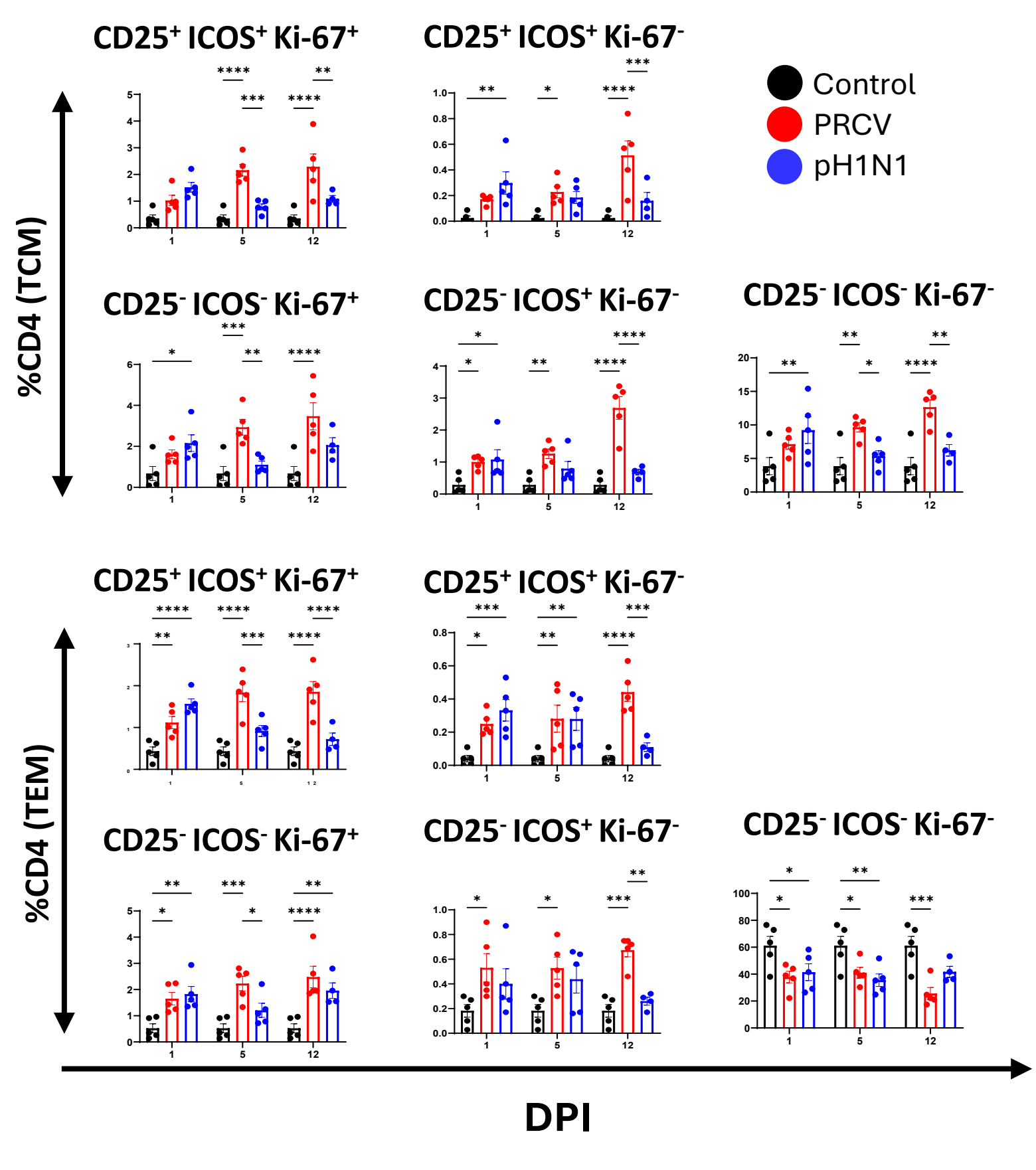

Supplementary Figure 8. Phenotypic analysis of CD4$^+$ T helper cells in BAL, PBMC, and TBLN following control, PRCV, and pH1N1 infection. Cryopreserved samples from BAL, PBMC, and TBLN were stained with antibodies against CD3, CD4, CD8α, CCR7, ICOS, CD25, and Ki-67 and analyzed by flow cytometry. Combinatorial gating was applied to identify CD4$^+$ T cell subsets based on the expression of these markers. **A)** t-SNE plots showing clustering of CD4$^+$ T cells across tissues. **B)** Table defining CD4$^+$ T cell subsets: TEMRA (CCR7$^-$ CD8α$^-$), TEM (CCR7$^-$ CD8α$^+$), naïve (CCR7$^+$ CD8α$^-$), and TCM (CCR7$^+$ CD8α$^+$), with further stratification based on CD25, ICOS, and Ki-67 expression.**(C)** Bar charts showing the frequency of CD4$^+$ TCM (CCR7$^+$ CD8α$^+$) and TEM (CCR7$^-$ CD8α$^+$) subpopulations in PBMCs from control (black), PRCV-infected (red), and pH1N1-infected (blue) pigs at 1, 5, and 12 DPI. Each point represents an individual animal; bars indicate the mean ± SEM. Statistical analysis was performed using two-way ANOVA with Bonferroni's multiple comparisons test. Asterisks indicate significance: *$p < 0.05$, **$p < 0.01$, ***$p < 0.001$, ****$p < 0.0001$.

A)

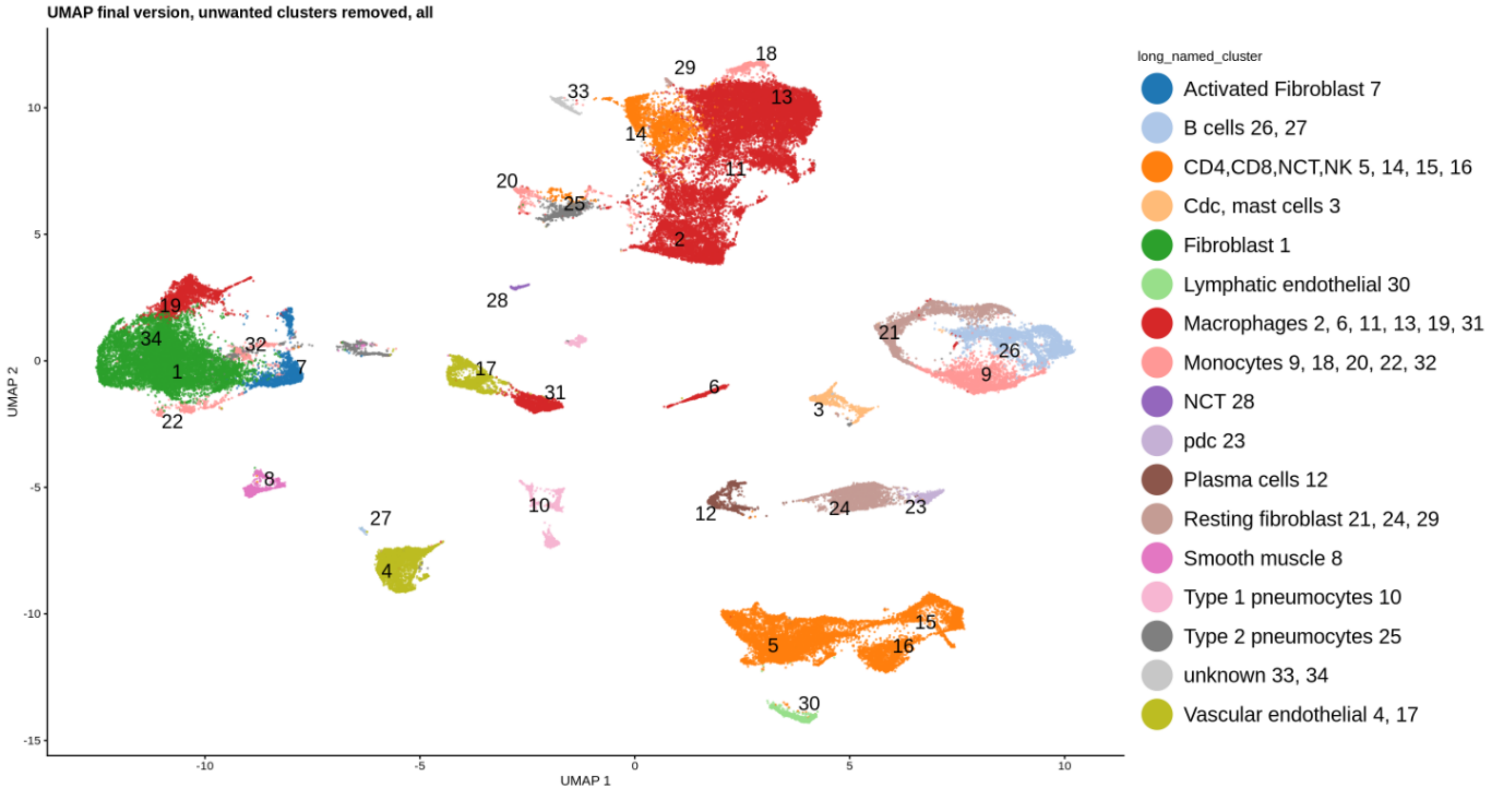

B)

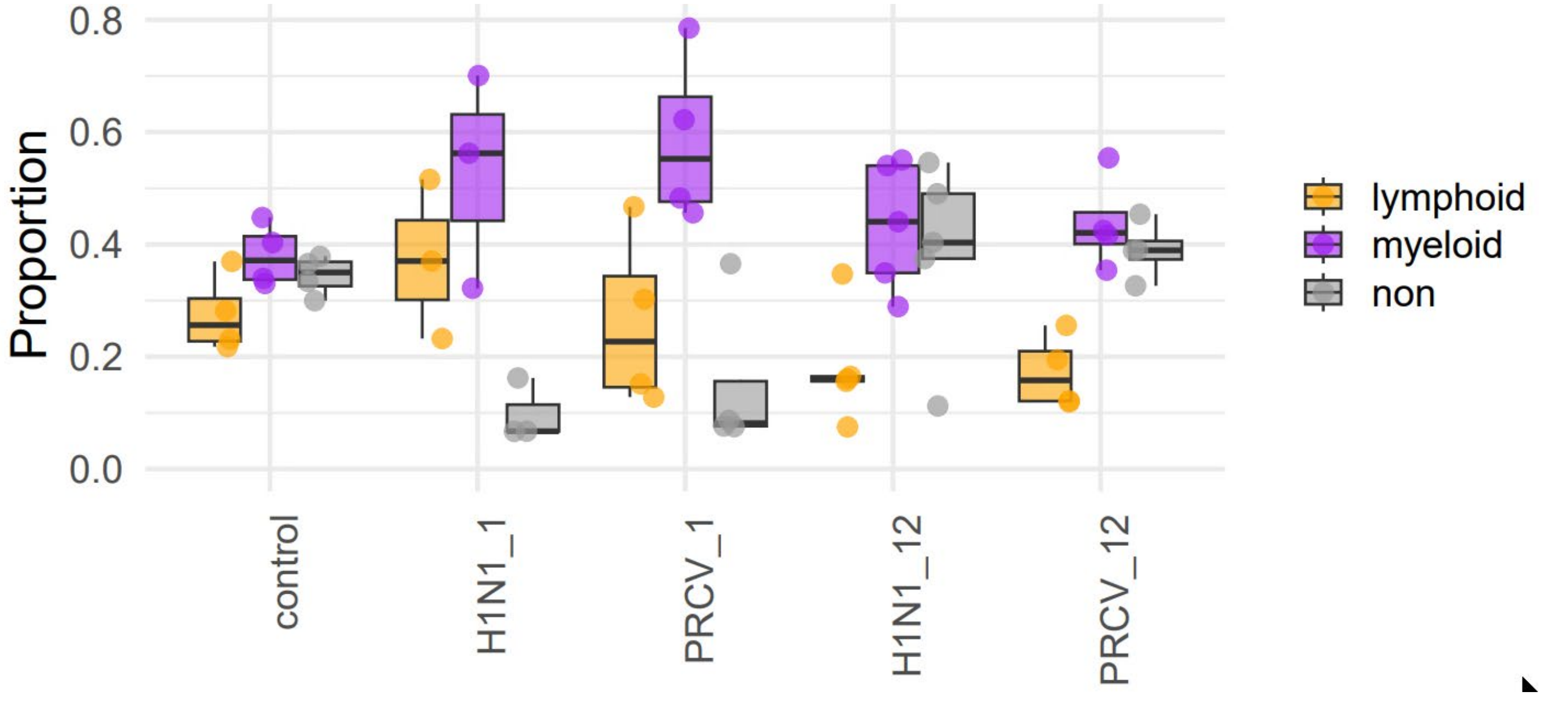

Supplementary Figure 9. Major lung-derived cells identified by scRNA-seq and their abundance in the time course. A) Uniform manifold approximation and projection (UMAP) of all cells that passed quality control from pig lung scRNA-seq analysis, coloured by cell-type and labelled with cluster numbers. **B)** Relative abundance of the three major cellular compartments identified by scRNA-seq (lymphoid, myeloid and non-immune cells) and their distribution across treatments and time points. Box plots show median (center line) and interquartile range. Whiskers indicate the range of the data excluding outliers; points beyond the whiskers are shown individually.

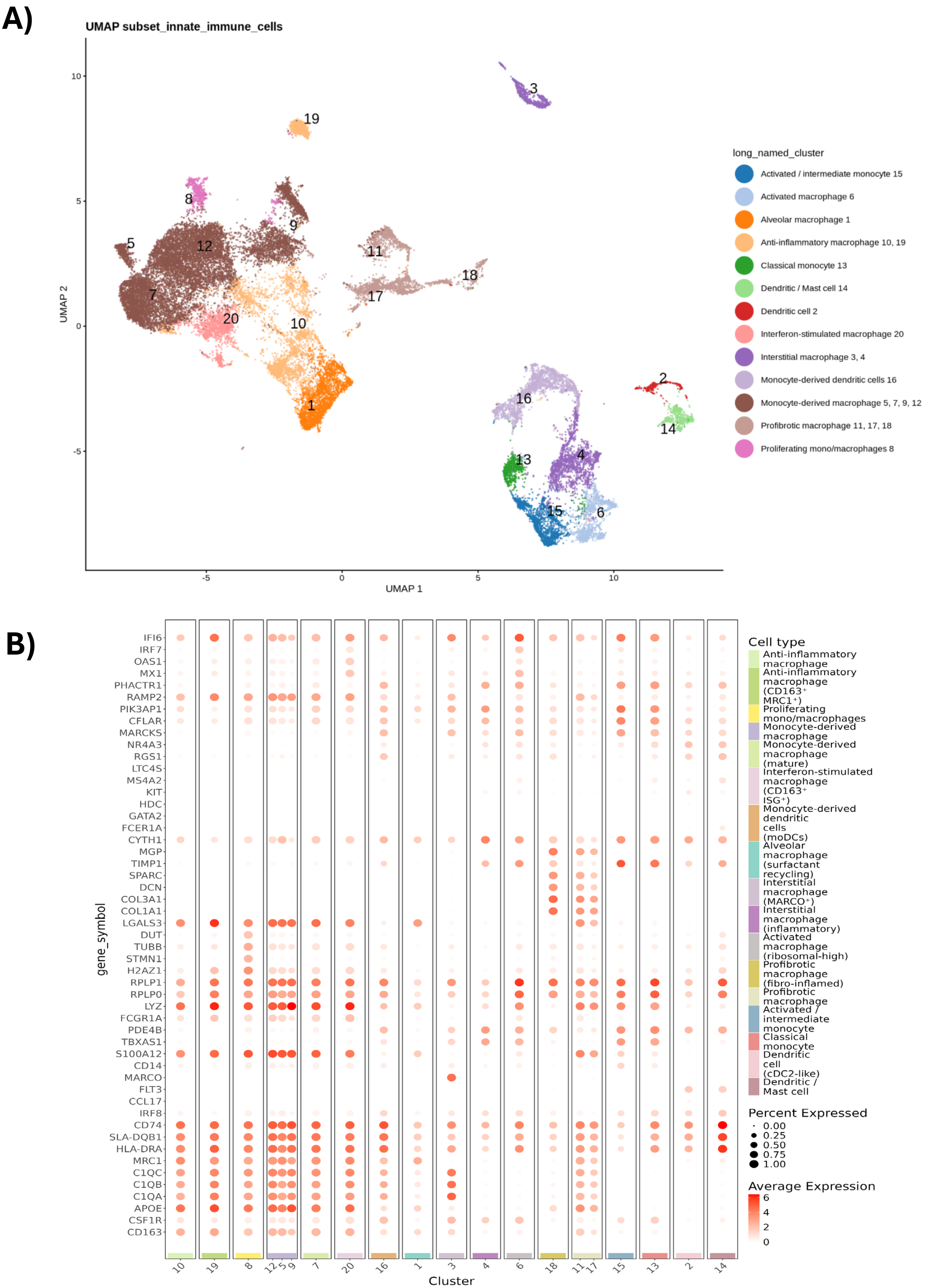

Supplementary Figure 10. Annotation of myeloid cells. A) UMAP of lung-derived myeloid cells coloured by cell-type and labelled with cluster numbers. **B)** Bubble plot showing marker genes and their average and percent expression used for cluster annotation.

A)

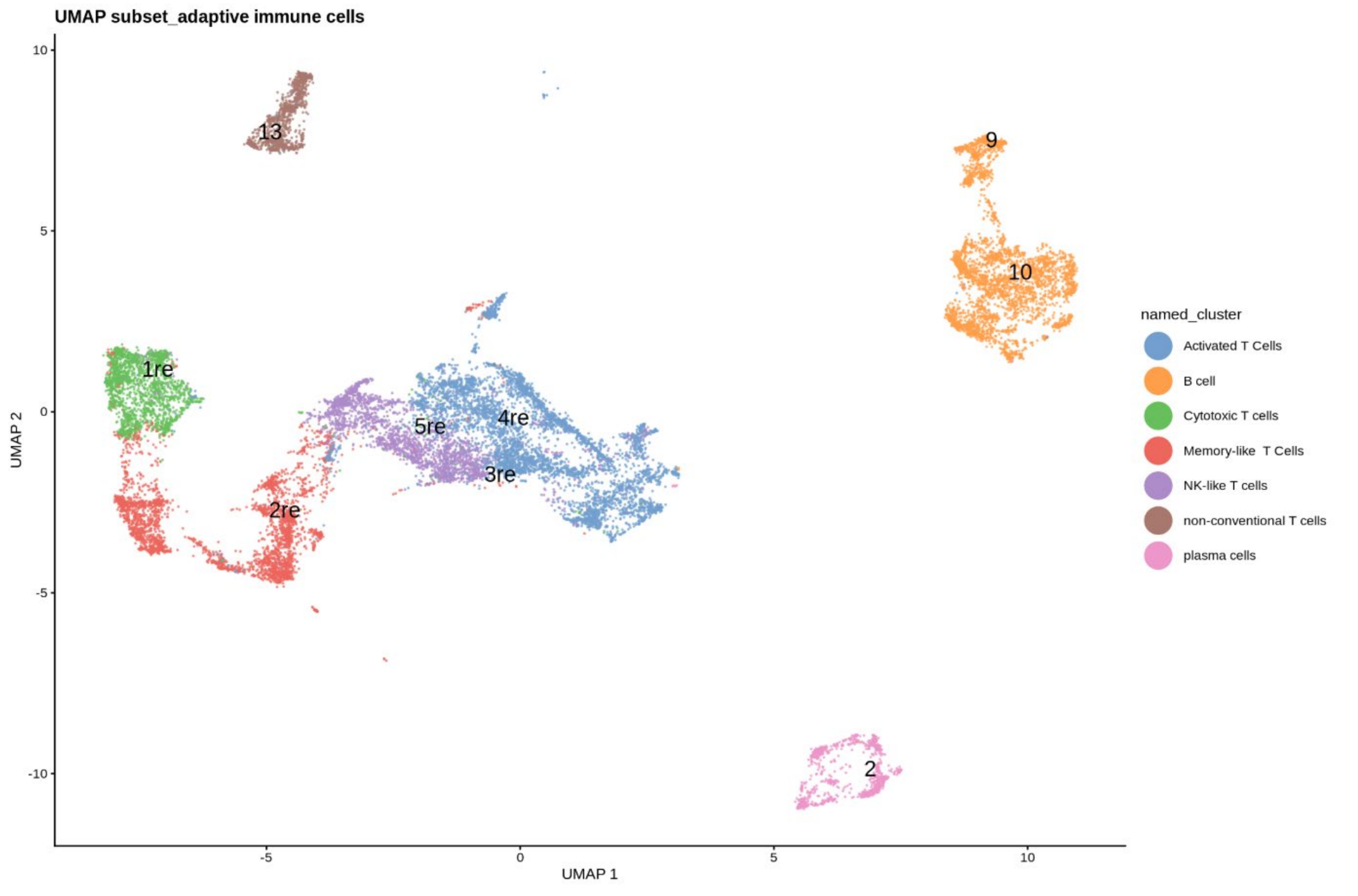

B)

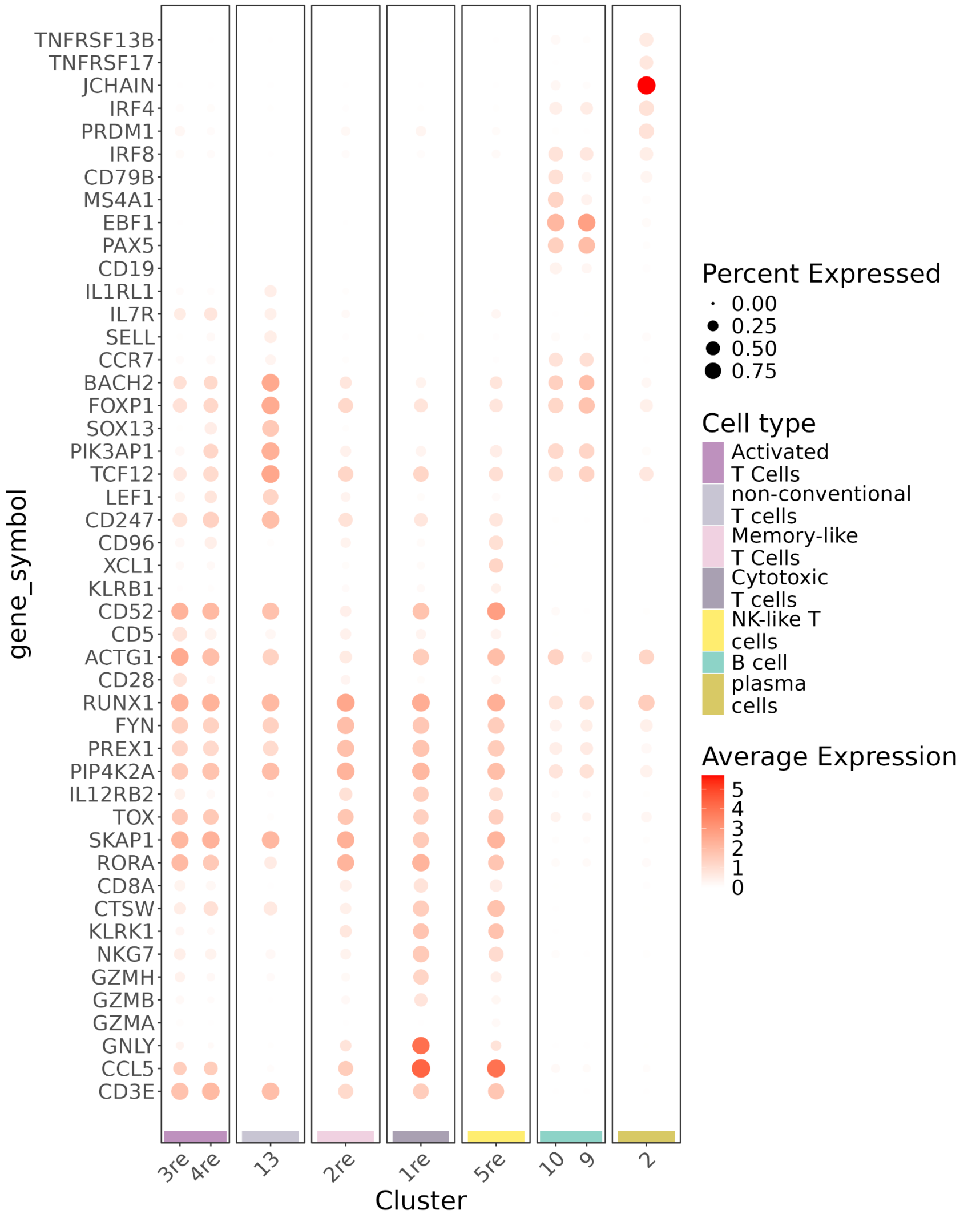

Supplementary Figure 11. Annotation of lymphoid cells. A) UMAP of lung-derived lymphocytes coloured by cell-type and labelled with cluster numbers. **B)** Bubble plot showing marker genes and their average and percent expression used for cluster annotation.

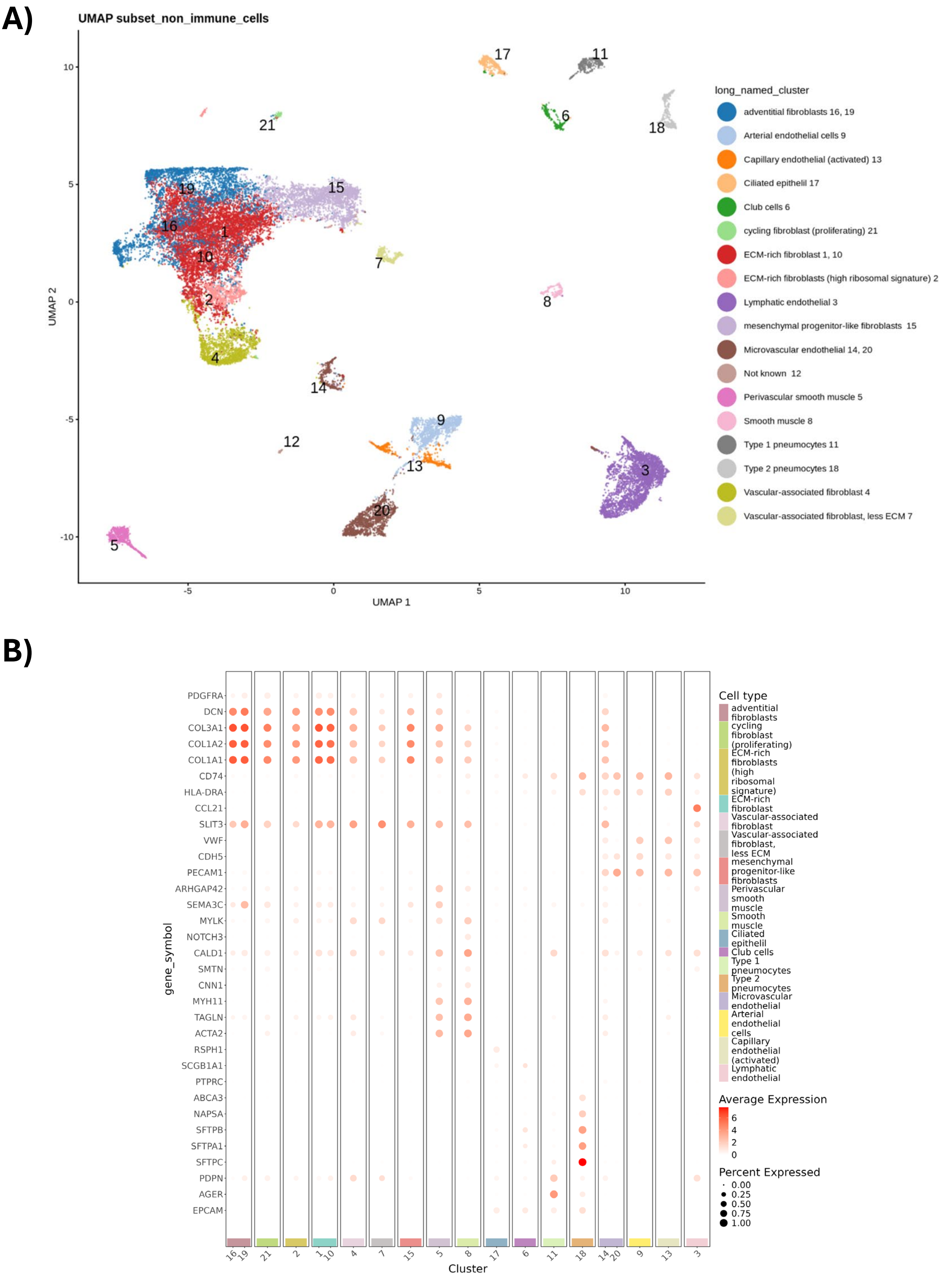

Supplementary Figure 12. Annotation of non-immune cells. A) UMAP of lung-derived non-immune cells coloured by cell-type and labelled with cluster numbers. **B)** Bubble plot showing marker genes and their average and percent expression used for cluster annotation.

A)

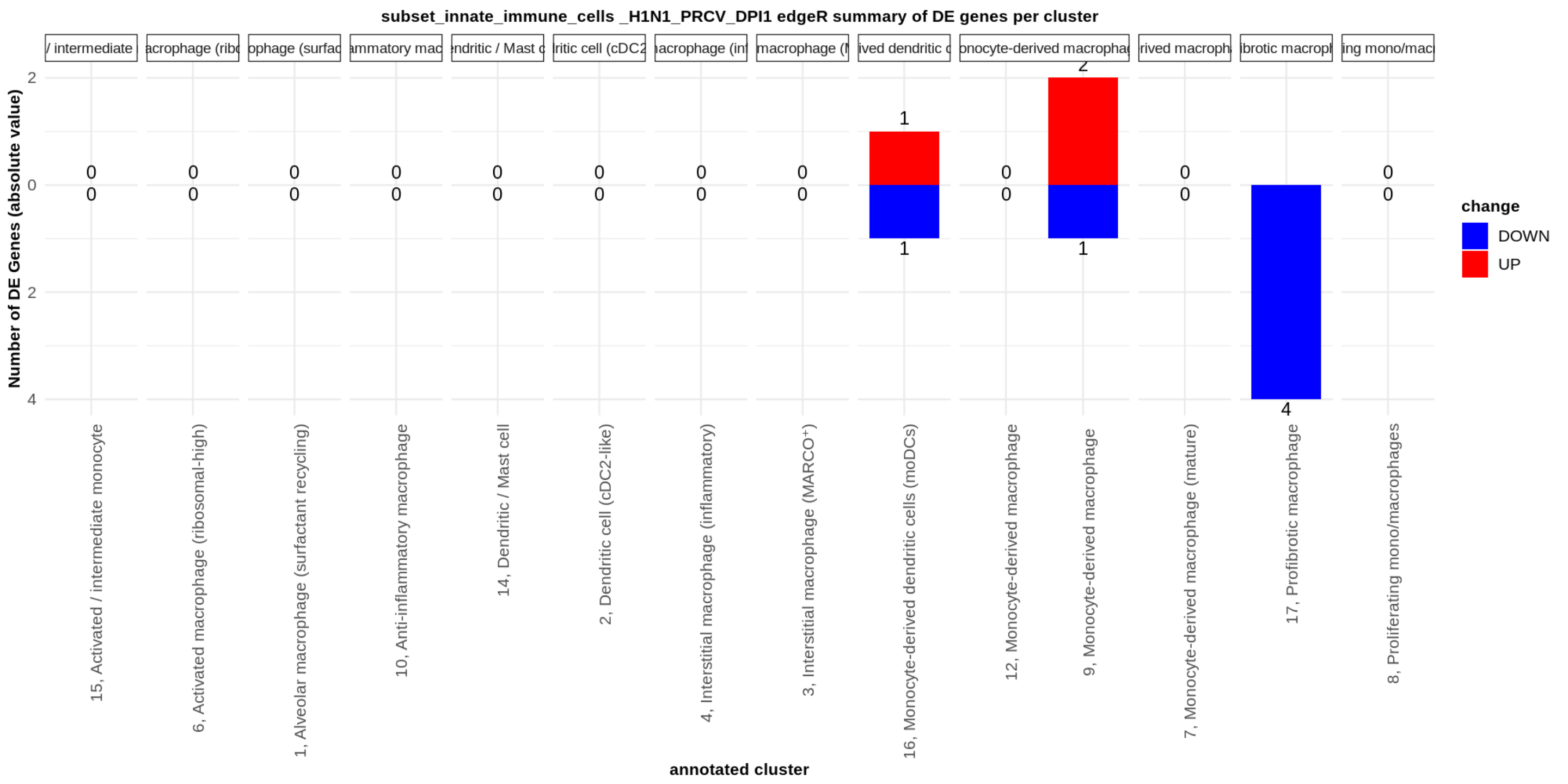

B)

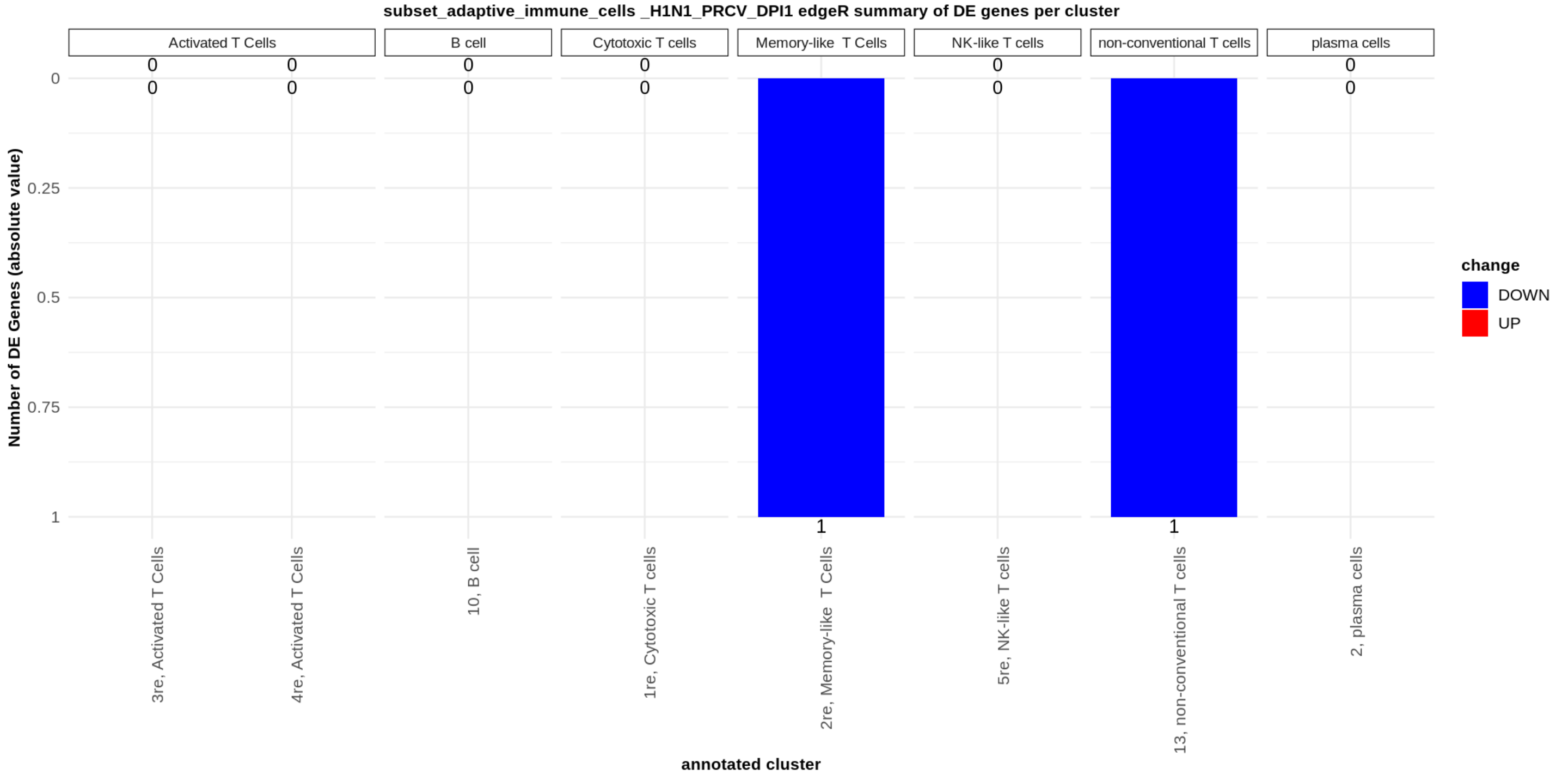

Supplementary Figure 13. Total DE genes between PRCV and pH1N1 infected pigs at 1 DPI. A) Myeloid cells. **B)** Lymphoid cells. There were no DEGs whose logFC pass our threshold and are considered as UP/DOWN in non-immune subset .

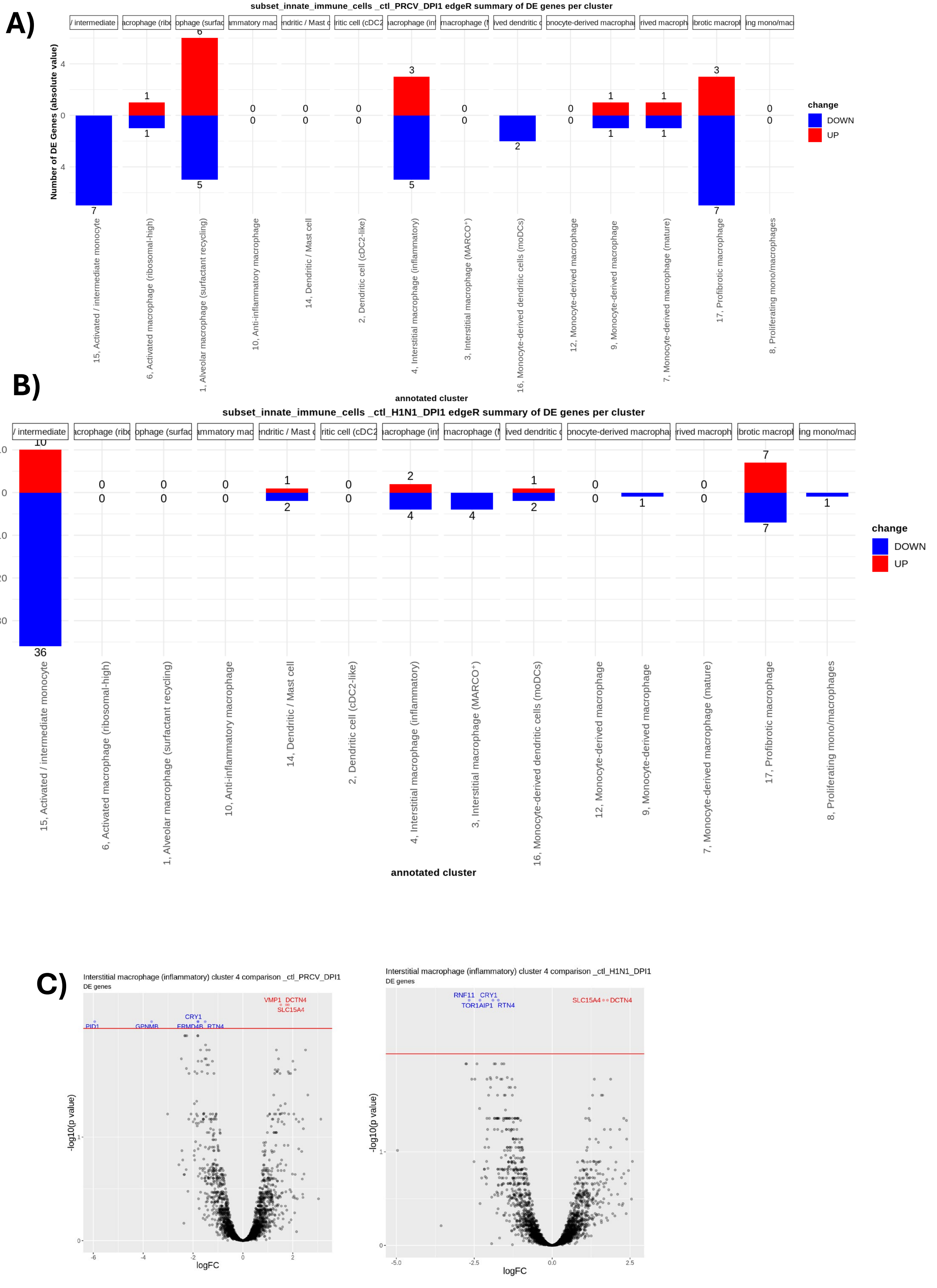

Supplementary Figure 14. DE genes in myeloid cells at 1 DPI. A) PRCV vs control **B)** pH1N1 vs control. **C)** Volcano plots of DE analysis for interstitial macrophages (cluster 4) showing PRCV vs control condition (left) and pH1N1 vs control (right).

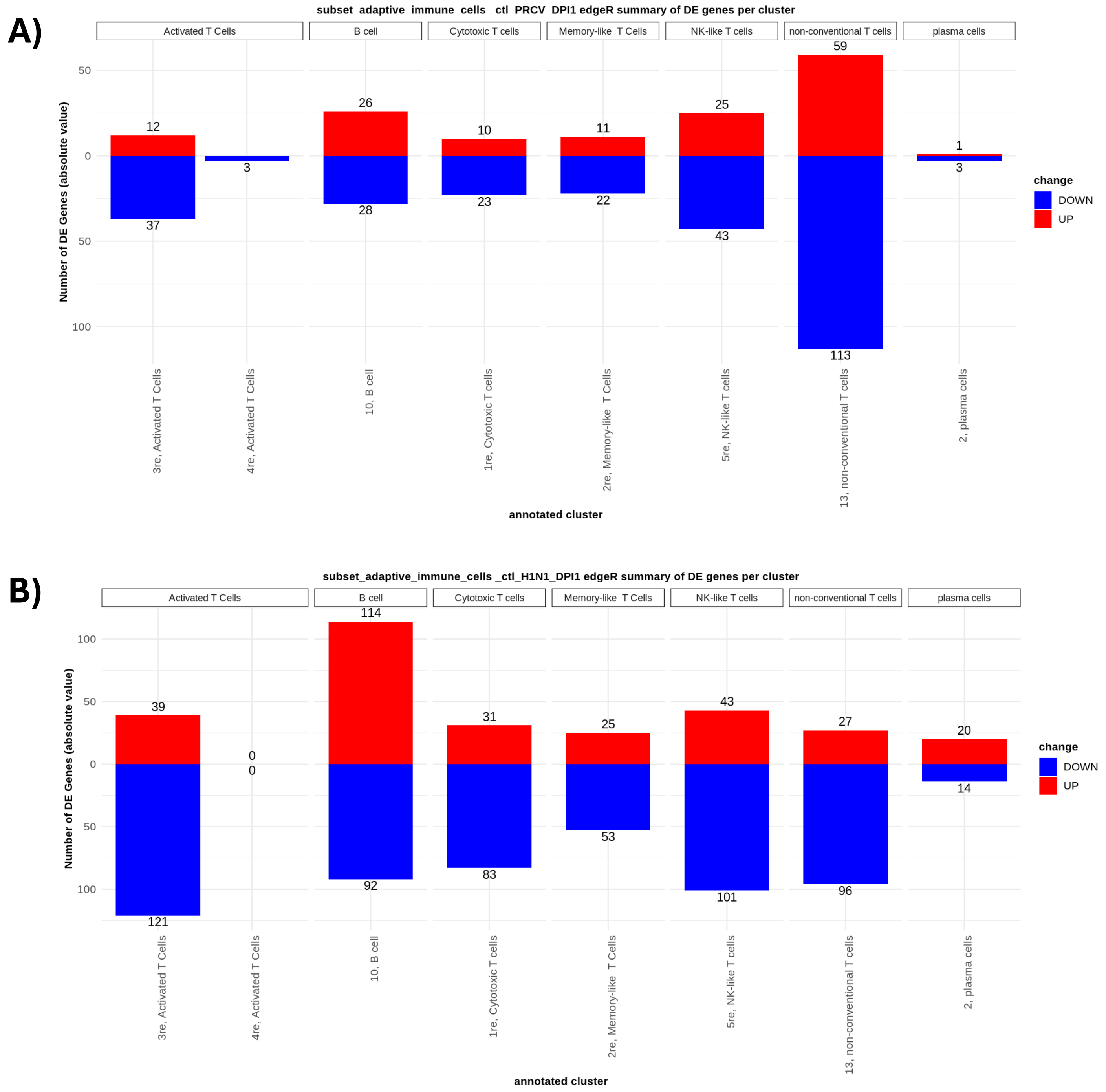

Supplementary Figure 15. DE genes in lymphoid cells at 1 DPI. A) PRCV vs control and **B)** **p**H1N1 vs control.

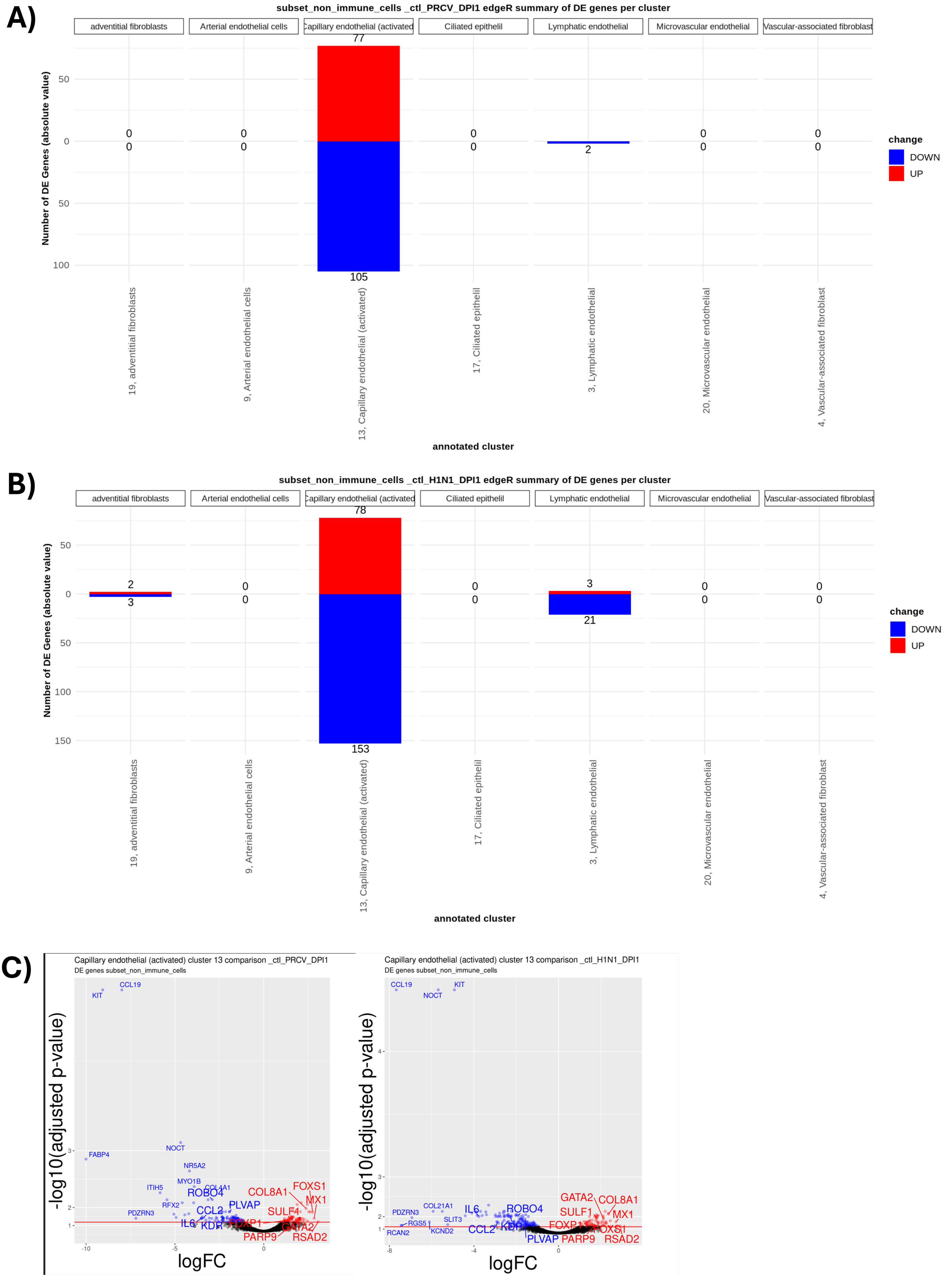

Supplementary Figure 16. DE genes in non-immune cells at 1 DPI. A) PRCV vs control **B)** pH1N1 vs control. **C)** Volcano plots of DE analysis for capillary endothelial cells (cluster 13) showing PRCV vs control condition (left) and pH1N1 vs control (right).

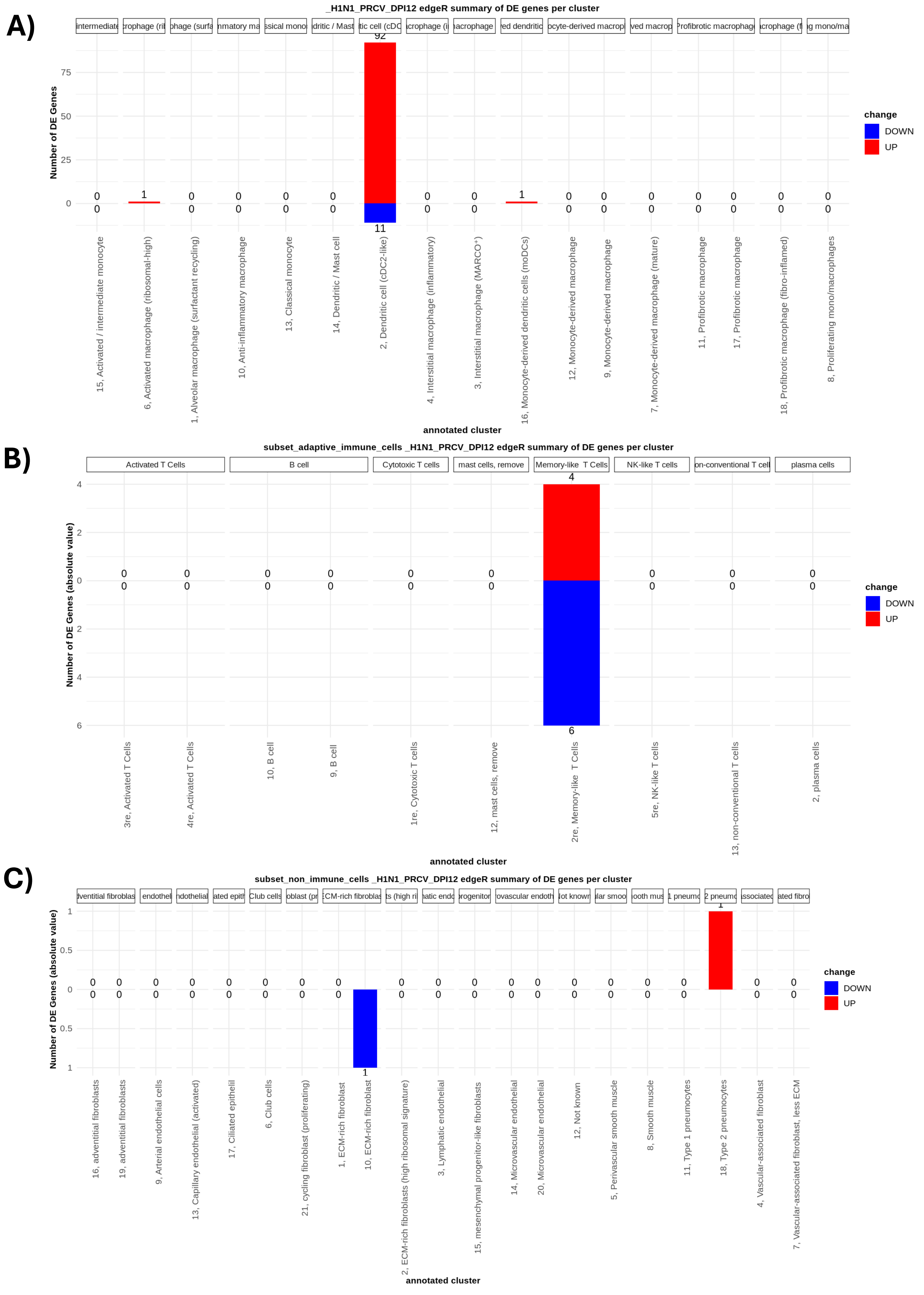

Supplementary Figure 17. Total DE genes between PRCV and pH1N1 infected pigs at 12 DPI. A) Myeloid cells. **B)** Lymphoid cells. **C)** Non-immune cells.

A)

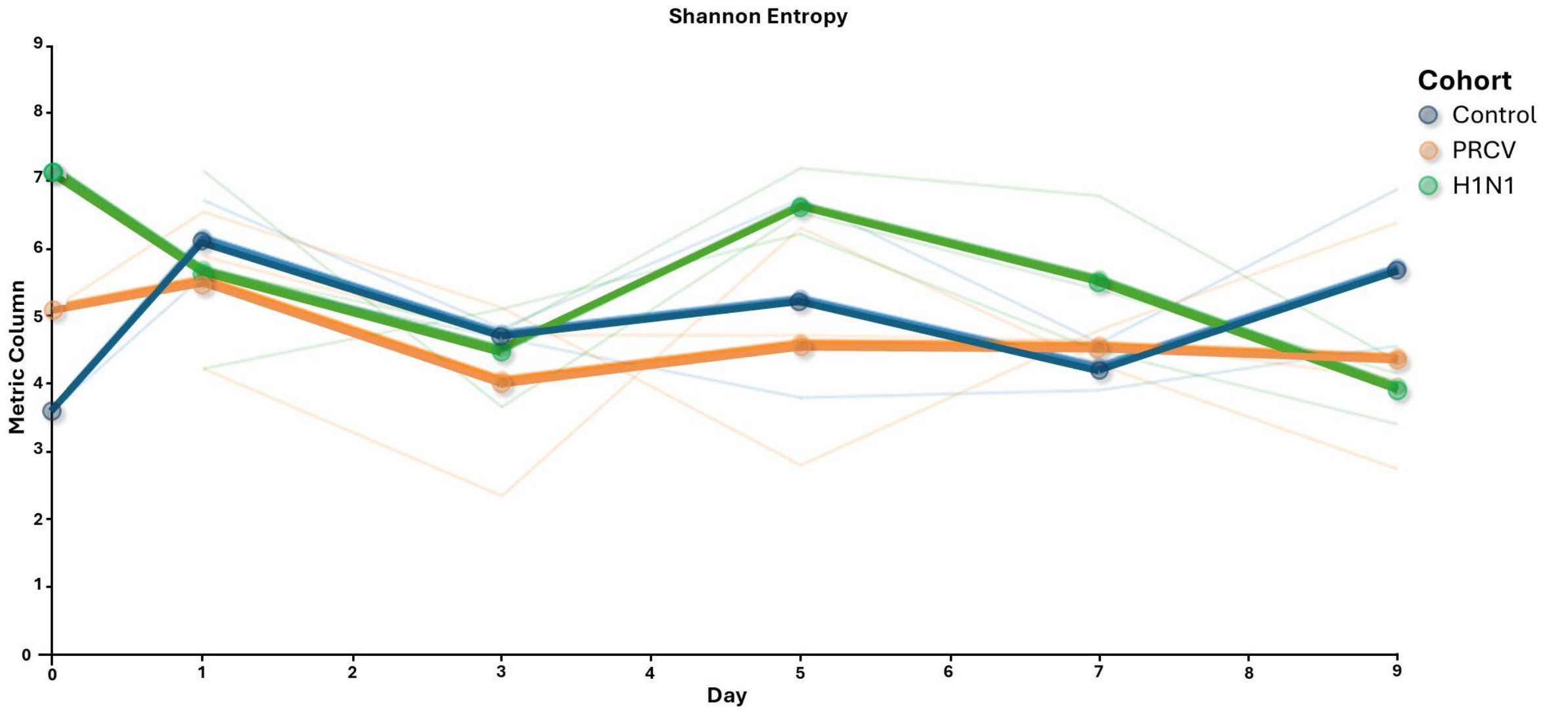

B)

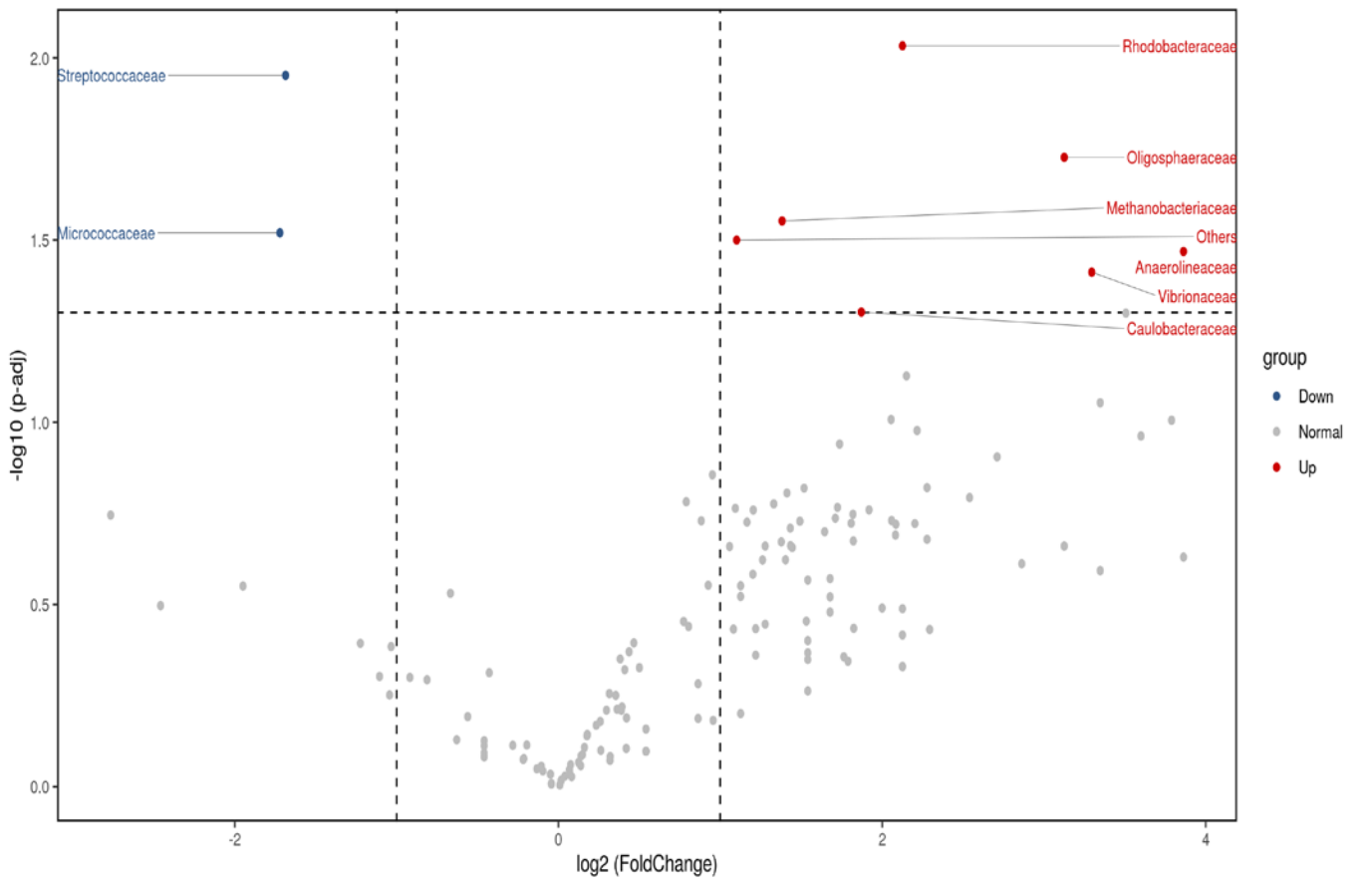

C)

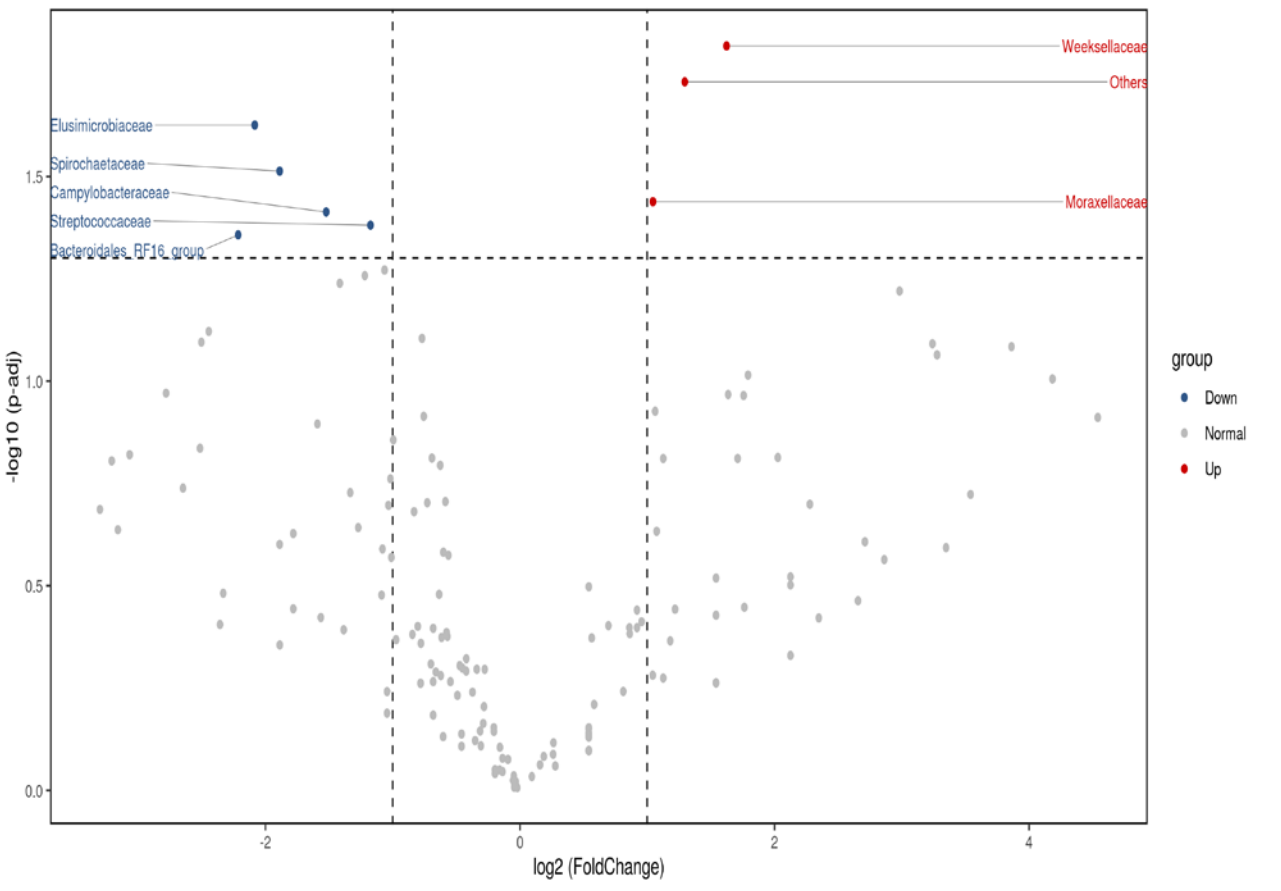

Supplementary Figure 18. A) Volatility plot illustrating the temporal stability of nasal microbiota of non-challenged (blue), PRCV-infected (orange), and pH1N1-infected (green) pigs, as indicated by the hannon diversity index over time. Faint coloued lines show the Shannon index of individual subjects, bold coloured lines show mean Shannon indices. **B)** Volcano plot indicating significantly up- and down-regulated bacterial families in the nasal microbiota of pigs infected with PRCV relative to non-challenged pigs. **C)** Volcano plot indicating significantly up- and down-regulated bacterial families in the nasal microbiota of pigs infected with pH1N1 relative to non-challenged pigs. The horizontal axis is the fold difference of the difference families , while the vertical axis is the p-value of the significant between-group difference test. Each point in the graph represents a differential family, where "Up" (red) represents the higher abundance of that differential family in the infected group than in the non-challenged group, while "Down" (blue) is the opposite.
