# Supplementary material for: Pathogen-specific immune responses might underlie divergent outcomes of coronavirus and influenza infection in the natural porcine host
Source: Commun Biol. 2026 May 30;9:1043. doi: 10.1038/s42003-026-10400-y (PMC13444145; doi:10.1038/s42003-026-10400-y)
Supplement: Supplementary file 2 [file 42003_2026_10400_MOESM2_ESM.pdf]

Description of Additional Supplementary Files

File name: Supplementary Data 1-5

Description: sc-RNA seq datasets

File name: Supplementary Data 6

Description: All numerical detaset
