# Supplementary material for: Pathogen-specific immune responses might underlie divergent outcomes of coronavirus and influenza infection in the natural porcine host
Source: Commun Biol. 2026 May 30;9:1043. doi: 10.1038/s42003-026-10400-y (PMC13444145; doi:10.1038/s42003-026-10400-y)
Supplement: Supplementary file 3 [file 42003_2026_10400_MOESM4_ESM.pdf]

nature portfolio

Corresponding author(s): COMMSBIO-25-11531A

Last updated by author(s): Mar 11, 2026

Reporting Summary

Nature Portfolio wishes to improve the reproducibility of the work that we publish. This form provides structure for consistency and transparency in reporting. For further information on Nature Portfolio policies, see our Editorial Policies and the Editorial Policy Checklist.

Statistics

For all statistical analyses, confirm that the following items are present in the figure legend, table legend, main text, or Methods section.

n/a	Confirmed	
☐	☒	The exact sample size (*n*) for each experimental group/condition, given as a discrete number and unit of measurement
☐	☒	A statement on whether measurements were taken from distinct samples or whether the same sample was measured repeatedly
☐	☒	The statistical test(s) used AND whether they are one- or two-sided<br>*Only common tests should be described solely by name; describe more complex techniques in the Methods section.*
☐	☒	A description of all covariates tested
☐	☒	A description of any assumptions or corrections, such as tests of normality and adjustment for multiple comparisons
☐	☒	A full description of the statistical parameters including central tendency (e.g. means) or other basic estimates (e.g. regression coefficient) AND variation (e.g. standard deviation) or associated estimates of uncertainty (e.g. confidence intervals)
☐	☒	For null hypothesis testing, the test statistic (e.g. *F*, *t*, *r*) with confidence intervals, effect sizes, degrees of freedom and *P* value noted<br>*Give P values as exact values whenever suitable.*
☒	☐	For Bayesian analysis, information on the choice of priors and Markov chain Monte Carlo settings
☒	☐	For hierarchical and complex designs, identification of the appropriate level for tests and full reporting of outcomes
☐	☒	Estimates of effect sizes (e.g. Cohen's *d*, Pearson's *r*), indicating how they were calculated

Our web collection on statistics for biologists contains articles on many of the points above.

Software and code

Policy information about availability of computer code

Data collection	*Provide a description of all commercial, open source and custom code used to collect the data in this study, specifying the version used OR state that no software was used.*
Data analysis	All R scripts used for processing and analysis of the scRNA-seq data are available at: https://github.com/LynnGoodnight/bsp248.

For manuscripts utilizing custom algorithms or software that are central to the research but not yet described in published literature, software must be made available to editors and reviewers. We strongly encourage code deposition in a community repository (e.g. GitHub). See the Nature Portfolio guidelines for submitting code & software for further information.

Data

Policy information about availability of data

All manuscripts must include a data availability statement. This statement should provide the following information, where applicable:

- Accession codes, unique identifiers, or web links for publicly available datasets
- A description of any restrictions on data availability
- For clinical datasets or third party data, please ensure that the statement adheres to our policy

Fastq and h5 files from sequencing are submitted to Gene Expression Omnibus (GEO), reference id will be release after publication. https://www.ncbi.nlm.nih.gov/geo.

Research involving human participants, their data, or biological material

Policy information about studies with human participants or human data. See also policy information about sex, gender (identity/presentation), and sexual orientation and race, ethnicity and racism.

Reporting on sex and gender	*Use the terms sex (biological attribute) and gender (shaped by social and cultural circumstances) carefully in order to avoid confusing both terms. Indicate if findings apply to only one sex or gender; describe whether sex and gender were considered in study design; whether sex and/or gender was determined based on self-reporting or assigned and methods used.<br>Provide in the source data disaggregated sex and gender data, where this information has been collected, and if consent has been obtained for sharing of individual-level data; provide overall numbers in this Reporting Summary. Please state if this information has not been collected.<br>Report sex- and gender-based analyses where performed, justify reasons for lack of sex- and gender-based analysis.*
Reporting on race, ethnicity, or other socially relevant groupings	*Please specify the socially constructed or socially relevant categorization variable(s) used in your manuscript and explain why they were used. Please note that such variables should not be used as proxies for other socially constructed/relevant variables (for example, race or ethnicity should not be used as a proxy for socioeconomic status).<br>Provide clear definitions of the relevant terms used, how they were provided (by the participants/respondents, the researchers, or third parties), and the method(s) used to classify people into the different categories (e.g. self-report, census or administrative data, social media data, etc.)<br>Please provide details about how you controlled for confounding variables in your analyses.*
Population characteristics	*Describe the covariate-relevant population characteristics of the human research participants (e.g. age, genotypic information, past and current diagnosis and treatment categories). If you filled out the behavioural & social sciences study design questions and have nothing to add here, write "See above."*
Recruitment	*Describe how participants were recruited. Outline any potential self-selection bias or other biases that may be present and how these are likely to impact results.*
Ethics oversight	*Identify the organization(s) that approved the study protocol.*

Note that full information on the approval of the study protocol must also be provided in the manuscript.

Field-specific reporting

Please select the one below that is the best fit for your research. If you are not sure, read the appropriate sections before making your selection.

☒ Life sciences ☐ Behavioural & social sciences ☐ Ecological, evolutionary & environmental sciences

For a reference copy of the document with all sections, see nature.com/documents/nr-reporting-summary-flat.pdf

Life sciences study design

All studies must disclose on these points even when the disclosure is negative.

Sample size	For studies to determine immune responses.Groups of 5 pigs will be required to obtain robust data , based on previous experience with reproducibility of virological, serological, immunological and histopathological parameters (Morgan et al J Immunol 2016 doi: 10.4049/jimmunol.1502632; Holzer et al J Immunol 2018 doi: 10.4049/jimmunol.1800142; Holzer et al Front Immunol 2019 doi: 10.3389/fimmu.2019.02625; Martini et al J Immunol 2021 doi: 10.4049/jimmunol.2001086, Keep et al Frontiers in Immunol 2022 doi: 10.3389/fimmu.2022.867707).
Data exclusions	No data were excluded from the analyses
Replication	To verify the reproducibility of the findings, 5 and 6 replicates were used per group. The ELISA, BAL flow cytometry, ELISPOT experiments and the plaque assays were performed twice, and the similar results were obtained both times, indicating that the findings are reproducible.
Randomization	The pigs were randomly assigned to groups at the beginning of the study.
Blinding	The pathologist was blinded to the samples when they were assessed for gross pathology during PM and subsequently during the assessment of histopathology. The laboratory workers only knew the pig number, but not to which group the pigs are assigned.

Reporting for specific materials, systems and methods

We require information from authors about some types of materials, experimental systems and methods used in many studies. Here, indicate whether each material, system or method listed is relevant to your study. If you are not sure if a list item applies to your research, read the appropriate section before selecting a response.

Materials & experimental systems

n/a	Involved in the study
☐	☒ Antibodies
☐	☐ Eukaryotic cell lines
☒	☐ Palaeontology and archaeology
☐	☒ Animals and other organisms
☒	☐ Clinical data
☒	☐ Dual use research of concern
☒	☐ Plants

Methods

n/a	Involved in the study
☒	☐ ChIP-seq
☐	☒ Flow cytometry
☒	☐ MRI-based neuroimaging

Antibodies

Antibodies used

The antibodies used for flow cytometry were the following:
Marker Clone Provider Dilution Fluorochrome
CD4 74-12-4 BD Biosciences 1/40 PE-Cy7
CD8β PPT23 Bio-Rad 1/20 PE
CD8α MIL12 Bio-Rad 1/10 StarBright Violet 515
CD3ε PPT3 Bio-Rad 1/80 StarBright Violet 570
CD27 b30c7 Bio-Rad 1/10 APC
CCR7 3D12 BD Biosciences 1/40 BV711
CD45RA MIL13 Bio-Rad 1/100 FITC
IFN-γ P2G10 BD Biosciences 1/400 PerCP-Cy 5.5
TNF Mab11 Biolegend 1/100 BV421
TCRδ PGBL22A Cambridge Bioscience 1/400 Unconjugated
Anti-IgG1 A85-1 BD Biosciences 1/400 BUV395
CD79α HM47 Biolegend 1/40 PerCP-Cy5.5
CD11c 3A8 Bio-Rad 1/20 FITC
Blimp1 3H2E8 Santa Cruz 1/10 AF647
IRF4 3E4 Fisher Scientific 1/5 PE
PAX5 1H9 BD Biosciences 1/10 BV421
BCL6 K112-91 BD Biosciences 1/20 BUV494
Ki67 B56 BD Biosciences 1/40 BUV395
Streptavidin BD Biosciences 1/500 BUV615
Tbet (eBio4B10 (4B10)) Fisher Scientific 1/20 PECy5
CD4 74-12-4 BD Biosciences 1/100 PerCP-Cy 5.5
CD3ε PPT3 Bio-Rad 1/80 StarBright Violet 570
CD8α 76-2-11 BD Biosciences 1/10 AF647
CD25 b30c7 Bio-Rad 1/20 FITC
ICOS C398.4A Biolegend 1/100 BV605
BCL6 K112-91 BD Biosciences 1/20 BUV494
FOXP3 FJK-16s Fisher Scientific 1/20 PE
KI67 B56 BD Biosciences 1/40 BUV395
CCR7 3D12 BD Biosciences 1/40 BV711

Antibodies used For IHC were as follows:
Anti-PRCV Monoclonal (clone DA-3) In house 1:10000 ER2 20 min1 Opal 690 (red) 1:300
nucleoprotein
Anti-Influenza Polyclonal Thermo Fisher Scientific 1:4000 ER2 20 min1 Opal 690 (red) 1:150
nucleoprotein
Pan-Cytokeratin Monoclonal (clone PCK-26) Novus Biotechne, Oxford, UK 1:100 ER1 20 min2 Opal 620 (yellow) 1:100
Iba1 Polyclonal FujiFilm Wako, Neuss, Germany 1:100 ER1 20 min2 Opal 520 (green) 1:300
- - - - ER2 20 min1 DAPI (blue) 1:10

Validation

The antibodies used were either pig specific or cross reactive for pig, as mentioned on the manufacturer's website or data sheet. All antibodies were titrated for the identification of the optimal concentration prior to the flow cytometry experiments.

Eukaryotic cell lines

Policy information about cell lines and Sex and Gender in Research

Cell line source(s)

MDCK (Madin-Darby Canine Kidney, CCL34) and ST (Swine Testi,CRL-1746) cells obtained from ATCC were used for plaque assay

Authentication

Describe the authentication procedures for each cell line used OR declare that none of the cell lines used were authenticated.

Mycoplasma contamination	*Confirm that all cell lines tested negative for mycoplasma contamination OR describe the results of the testing for mycoplasma contamination OR declare that the cell lines were not tested for mycoplasma contamination.*
Commonly misidentified lines (See ICLAC register)	*Name any commonly misidentified cell lines used in the study and provide a rationale for their use.*

Animals and other research organisms

Policy information about studies involving animals; ARRIVE guidelines recommended for reporting animal research, and Sex and Gender in Research

Laboratory animals	A total of 36 outbred female pigs, aged between 5 to 7 weeks and genetically composed of one-quarter Large White, one-quarter Landrace, and one-half Hampshire breeds
Wild animals	The study did not involve wild animals.
Reporting on sex	Finds apply to one one sex (Female)
Field-collected samples	The study did not involve samples collected from the field
Ethics oversight	Animal experiments were approved by the ethical review processes of The Pirbright Institute and the Animal and Plant Health Agency (APHA) according to the UK Animals (Scientific Procedures) Act 1986 under project licenses PP7764821 and PP2064443

Note that full information on the approval of the study protocol must also be provided in the manuscript.

Plants

Seed stocks	*Report on the source of all seed stocks or other plant material used. If applicable, state the seed stock centre and catalogue number. If plant specimens were collected from the field, describe the collection location, date and sampling procedures.*
Novel plant genotypes	*Describe the methods by which all novel plant genotypes were produced. This includes those generated by transgenic approaches, gene editing, chemical/radiation-based mutagenesis and hybridization. For transgenic lines, describe the transformation method, the number of independent lines analyzed and the generation upon which experiments were performed. For gene-edited lines, describe the editor used, the endogenous sequence targeted for editing, the targeting guide RNA sequence (if applicable) and how the editor was applied.*
Authentication	*Describe any authentication procedures for each seed stock used or novel genotype generated. Describe any experiments used to assess the effect of a mutation and, where applicable, how potential secondary effects (e.g. second site T-DNA insertions, mosiacism, off-target gene editing) were examined.*

Flow Cytometry

Plots

Confirm that:

☐ The axis labels state the marker and fluorochrome used (e.g. CD4-FITC).

☐ The axis scales are clearly visible. Include numbers along axes only for bottom left plot of group (a 'group' is an analysis of identical markers).

☒ All plots are contour plots with outliers or pseudocolor plots.

☒ A numerical value for number of cells or percentage (with statistics) is provided.

Methodology

Sample preparation	Cryopreserved BAL, TBLN and PBMC were thawed and 106 cells per well were plated in duplicates in U-bottom 96-well plates. Cells were stimulated overnight with PRCV and pH1N1 live virus at a MOI of 1, or for 5 hours with overlapping peptide pools covering the S/HA or N/NP proteins for PRCV/pH1N1-infected animals and 3 control animals for each group. One well per animal was incubated with media alone (RPMI1640 with GlutaMAX™, 100 IU/ml penicillin, 100 µg/ml streptomycin, and 10% FBS) as a negative control, and positive control wells were incubated with a 1/500 dilution of a phorbol 12-myristate 13-acetate (PMA) and ionomycin cocktail (BioLegend). A 1/1000 dilution of Brefeldin A (GolgiPlug™, BD Biosciences) was added to all wells 4 hours before harvest. All incubations were carried out at 37°C with 5% CO2.<br>Following harvest, cells were centrifuged at 500 × g for 4 minutes and washed twice with PBS. Unconjugated anti-swine TCRδ mAbs were added to all samples at 4°C for 20 minutes in the dark. Then, cells were washed twice with PBS and incubated with rat anti-mouse IgG1-BUV395 for 20 minutes at 4°C in the dark. Following two washes, a blocking step was performed using a 1/20 dilution of mouse sera for 20 minutes at 4°C in the dark. After blocking, a cocktail of surface antibodies was added. Fixation and permeabilization were performed using 100 µl of BD Cytofix/Cytoperm (BD Biosciences, Cat. no: 555028). Subsequently, cells were incubated with antibodies against IFNγ and TNF for 30 minutes at 4°C in the dark.<br>Samples were analysed on a Cytek Aurora full spectrum flow cytometer (Cytek Biosciences, Fremont, CA, United States), equipped with 5 lasers: UV 355 nm, violet 405 nm, blue 488 nm, yellow-green 561 nm, red 640 nm and 64 fluorescence detection channels (UV: 16, violet: 16, blue: 14, yellow-green: 10, red: 8). Spectral unmixing was performed by the SpectroFlo

software (version 3.2.1, Cytek Biosciences). t-SNE followed by FlowSOM analysis was performed on concatenated samples from stimulated cells using FlowJo software version 10.10.0 to explore heatmap of marker expression. Conventional gating strategies were then applied, and results from the stimulated wells were subtracted from those of the medium-treated wells.

Ex vivo phenotypic analysis of B and T cells by flow cytometry. Thawed cell suspensions from BAL, TBLN and PBMC were resuspended in 10ml of complete RPMI1640 medium and centrifuged at 500×g for 5minutes. Subsequently, 2×10^{6} cells per sample were transferred into 96-well U-bottom plates and washed twice with PBS. A cocktail of primary antibodies specific for either B cells or T cells was added to the wells and incubated for 20minutes at 4°C. Plates were then washed twice with PBS.

For intracellular staining, fixation and permeabilization were performed using the eBioscience Foxp3/Transcription Factor Fixation/Permeabilization concentrate and diluent (Invitrogen) according to manufacturer's instructions. After two PBS washes, antibodies specific for intracellular markers were added and incubated for 30minutes at 4°C.

For the detection of antigen-specific B cells, biotinylated recombinant PRCV spike or pH1N1 HA proteins were added to the appropriate wells and incubated for 40minutes at room temperature in the dark. Following two PBS washes, streptavidin-conjugates were added to detect the biotinylated mAbs. Finally, cells were resuspended in PBS.

Samples were acquired on the Cytek Aurora flow cytometer introduced above and data were analyzed using FlowJo software.

For T helper cell phenotyping analysis, $CD3^{+}CD4^{+}Foxp3^{-}Bcl6^{-}$ cells were concatenated across all samples. t-SNE analysis was performed followed by combinatorial gating using CCR7, CD8α, CD25, ICOS, and Ki-67 markers.

Instrument

Cytek Aurora full spectrum flow cytometer (Cytek Biosciences, Fremont, CA, United States), equipped with 5 lasers: UV 355 nm, violet 405nm, blue 488nm, yellow-green 561nm, red 640nm and 64 fluorescence detection channels (UV: 16, violet: 16, blue: 14, yellow-green: 10, red: 8)

Software

FlowJo V10 (BD)

Cell population abundance

Describe the abundance of the relevant cell populations within post-sort fractions, providing details on the purity of the samples and how it was determined.

Gating strategy

The gating was performed following spectral unmixing. For ICS, cells were first gated on lymphocytes (FSC-A, SSC-A), singlets (FSC-A, FSC-H), and live cells (FSC-A, NIR negative). From live singlet lymphocytes, gates were applied on either CD4 (CD4-PE-Cy7, CD3E-StarBright Violet 570), CD8-beta (CD8B-PE, CD3E-StarBright Violet 570), or γδ T cells (TCRδ-BUV395, CD3E-StarBright Violet 570). Samples from all animals were then concatenated, followed by t-SNE analysis and FlowSOM clustering to evaluate the phenotype of cytokine-secreting cells using the following markers: CD8α-StarBright Violet 515, CD27-APC, CCR7-BV711, CD45RA-FITC, IFNγ-PerCP-Cy5.5, and TNF-BV421, analysed in FlowJo v10.

For manual gating of ICS data, cells were gated on lymphocytes, singlets, live cells, then on CD4, CD8, or γδ T cells, followed by IFNγ and TNF. Quadrants were applied to identify single- and double-positive cytokine-producing cells.

For antigen-specific plasma cells and B cells, lymphocytes and singlets, live cells were first gated, followed by total B cells (PAX5-BV421, CD79a-PerCP-Cy5.5). Within total B cells, B cells were identified as IRF4-PE negative and Blimp-1-Alexa Fluor 647 negative, while plasma cells were IRF4 positive and Blimp-1 positive. In TBLN, germinal centre B cells were further gated using BCL6-BUV494 and Ki-67. In PBMC, after gating on B cells, ABC-like cells were identified as CD11c-FITC and T-bet-PE-Cy5 double positive.lastly, we gated on sterptavidin-BUV615 for spike/HA specific B cell and plasma cell population.

For Tregs, cells were gated on lymphocytes, singlets, live cells, then CD4-PerCP-Cy5.5 and CD3-StarBright Violet 570 double-positive cells. Tregs were identified as FOXP3-PE and CD25-FITC positive.

For Tfh cells, gating was performed on lymphocytes, singlets, live cells, CD4-PerCP-Cy5.5 and CD3-StarBright Violet 570 double positives, then ICOS-BV605 and BCL6-BUV494.

For CD4 memory phenotyping and activation markers, lymphocytes, singlets, live cells, and CD4-PerCP-Cy5.5/CD3-StarBright Violet 570 double-positive cells were gated. FOXP3-PE and BCL6-BUV494 positive cells were excluded. Combination gating was then applied using CCR7-BV711, CD8α-Alexa Fluor 647, CD25-FITC, ICOS-BV605, and Ki-67-BUV395.

☒ Tick this box to confirm that a figure exemplifying the gating strategy is provided in the Supplementary Information.
